# Interspecific diversity in the neuronal composition of the mammalian cortex arises from heterochrony in neurogenesis

**DOI:** 10.1038/s44318-026-00806-z

**Published:** 2026-05-22

**Authors:** Yuki Y Yamauchi, Xuanhao D Sheu, Rafat Tarfder, Takuma Kumamoto, Jun Hatakeyama, Haruka Sato, Pauline Rouillard, Merve Bilgic, Shuto Deguchi, Tomonori Nakamura, Yusuke Kishi, Kazuo Emoto, Ikuo K Suzuki

**Affiliations:** 1 Laboratory of functional genomics for human evolution, Graduate School of Frontier Biosciences, The University of Osaka, Osaka, Japan; 2 Department of Biological Sciences, Graduate School of Science, The University of Tokyo, Tokyo, Japan; 3 Developmental Neuroscience Project, Department of Basic Medical Sciences, Tokyo Metropolitan Institute of Medical Science, Tokyo, Japan; 4 Department of Brain Morphogenesis, Institute of Molecular Embryology and Genetics, Kumamoto University, Kumamoto, Japan; 5 Laboratory of Molecular Neurobiology, Institute for Quantitative Biosciences, The University of Tokyo, Tokyo, Japan; 6 Institute for the Advanced Study of Human Biology (WPI-ASHBi), Kyoto University, Kyoto, Japan; 7 Department of Anatomy and Cell Biology, Graduate School of Medicine, Kyoto University, Kyoto, Japan; 8 The Hakubi Center for Advanced Research, Kyoto University, Kyoto, Japan; 9 Laboratory of Molecular Biology, Graduate School of Pharmaceutical Sciences, The University of Tokyo, Tokyo, Japan; 10 Division of Aging Biology, Research Institute for Science and Technology, Tokyo University of Science, Chiba, Japan; 11 International Research Center for Neurointelligence, The University of Tokyo, Tokyo, Japan

**Keywords:** Development, Evolution & Ecology, Neuroscience

## Abstract

Mammals share a laminar cerebral cortex, with excitatory neuron subtypes organized in distinct layers. Although this framework is conserved, subtype balance varies markedly between species due to largely unknown mechanisms. Here, we show that species-specific neuronal composition arises from non-uniform scaling of the temporal dynamics of neurogenesis. Comparative histology of eight mammalian species reveals a significant, rat-specific expansion of the deep layer in the somatosensory cortex. This feature of the rat cortex results from a specific extension of the early neurogenetic phase of deep-layer neuron production before transitioning to the upper layer, as confirmed by neuronal birthdating and single-cell transcriptomics. The duration of deep-layer neuron production is regulated by a genetic program controlling neural progenitor cell aging, including canonical Wnt signaling. Comparative single-cell transcriptomics revealed that cortical progenitor cells in rats exhibit significantly elevated Wnt ligand expression. Therefore, while sequential cortical neurogenesis is conserved, its progression is non-uniformly scaled between species. Precise heterochronic fine-tuning allows evolutionary refinement of cellular configuration without drastic remodeling of the conserved corticogenesis program.

## Synopsis

Cortical organization is broadly conserved in mammalian brains, but the mechanisms underlying species-specific differences in neuron subtype composition remain unclear. This study shows that extended early neurogenesis in rats increases the abundance of deep-layer neurons via elevated Wnt signaling, revealing temporal scaling as a mechanism for evolutionary diversification.

- A comparison of eight mammalian species reveals a rat-specific expansion of deep-layer neurons.
- Extended early neurogenesis promotes the deep-layer expansion observed in rats.
- Neural progenitor cells in rats show elevated Wnt ligand expression.
- A conserved neurogenetic sequence with heterochronic scaling between species contributes to the diversification of the mammalian cortex.

## Abstract (web summary)

Increased activity of Wnt signaling contributes to a prolonged developmental phase of deep-layer neurogenesis in rats.

## Graphical abstract

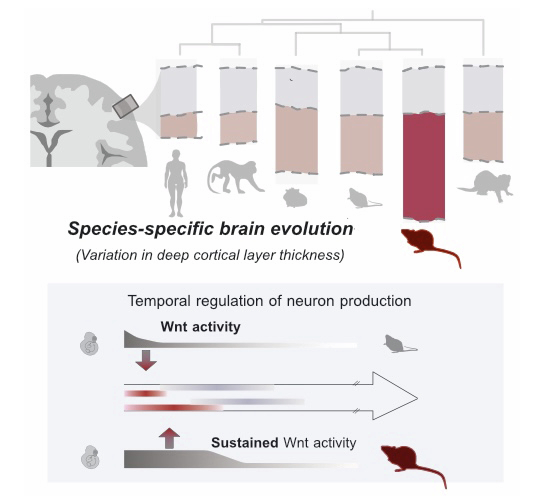

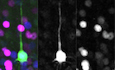

## Introduction

Animals adapt their neural circuits to navigate specific ecological niches (Kaas et al, 2016; Butler and Hodos, 2005), a process requiring the fine-tuning of brain architecture. In mammals, the cerebral cortex serves as the structural foundation for advanced cognitive functions, characterized by a highly organized repertoire of neuronal subtypes distributed across six tangential layers (Matho et al, 2021; Greig et al, 2013; Yuste et al, 2020). While the fundamental logic of laminar organization is evolutionarily conserved, significant quantitative variations emerge across species, presumably reflecting functional adaptations (Yuste et al, 2020; Hodge et al, 2019; Berg et al, 2021). Paradoxically, despite the importance of these variations, research has predominantly focused on the conserved aspects of cortical architecture. Consequently, the degree of evolutionary plasticity inherent in these circuits and the cell-intrinsic mechanisms driving their modification remain profoundly enigmatic.

The fundamental quest in cortical development lies in understanding how the dosage and balance of neuronal subtypes are autonomously regulated. During corticogenesis, radial glial (RG) progenitors in the ventricular zone (VZ) generate distinct excitatory lineages in a stereotyped temporal sequence (Tamamaki et al, 2001; Noctor et al, 2001; Malatesta et al, 2000), sequentially producing deep-layer (DL) neurons followed by upper-layer (UL) neurons (Vanderhaeghen and Polleux, 2023; Desai and McConnell, 2000; Rakic, 2009; Jabaudon, 2017). While this neurogenic program is robust and highly evolutionarily conserved, it is not immutable. For instance, the human cortex exhibits a massive expansion of UL neurons, attributed to an extended late-neurogenic phase (Berg et al, 2021; Bakken et al, 2016; Vanderhaeghen and Polleux, 2023; Hutsler et al, 2005; Suzuki, 2022, 2020; Stepien et al, 2021). However, it remains unclear whether evolutionary adaptations are limited to such “terminal expansions” or if the entire temporal coordinates of neurogenesis can be flexibly scaled to alter subtype composition.

Crucially, the fascination with human-specific traits has left other mammalian lineages (Suzuki, 2020, 2022; Vanderhaeghen and Polleux, 2023; Suzuki and Vanderhaeghen, 2015; Molnár et al, 2019), such as rodents, comparatively underexplored despite their remarkable diversity (Catzeflis et al, 1992; Fabre et al, 2012). Mice and rats, the gold-standard models of modern biology, diverged approximately 13.1 million years ago (Hedges et al, 2015) and exhibit distinct brain volumes and behaviors. Yet, the evolutionary divergence shaping their respective cortical landscapes has seldom been studied in depth at a mechanistic level (Bresee et al, 2023). This lack of comparative insight represents a significant gap in our understanding of how conserved developmental programs give rise to species-specific brain architectures.

In this study, we demonstrate that the cortical landscape is more plastic than previously envisioned. By comparing the primary somatosensory cortex across eight mammalian species, we identified a striking divergence: the rat possesses an exceptionally thick DL, even when compared to closely related rodents. Through single-cell resolution analysis and clonal lineage tracing, we show that rat RG progenitors are intrinsically programmed to generate a disproportionately higher number of DL neurons, while maintaining a conserved UL pool. Remarkably, our findings reveal that the temporal regulation of neurogenesis scales in a non-uniform manner between species. We propose that such species-specific “temporal scaling” of progenitor activity serves as a fundamental principle for the evolutionary diversification of the mammalian neocortex.

## Results

### Species-specific expansion of DL neurons in the rat somatosensory cortex

**Figure 1:**
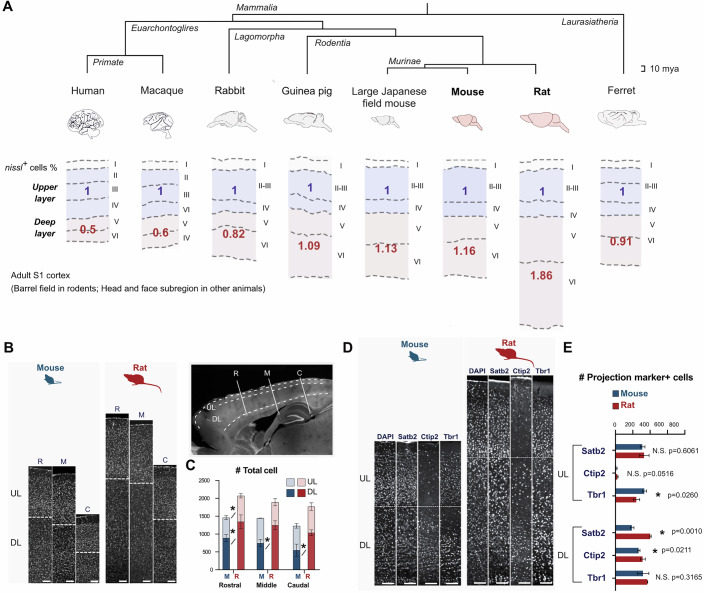
Histological comparison of the mammalian cerebral cortices highlighting the exceptional expansion of DL in rats. (**A**) Phylogenetic tree of eight mammalian species with the upper-to-deep layer neuron ratio in the somatosensory cortex. Neuronal numbers were quantified using Nissl-stained brain sections from the corresponding cortical region at the adult stage. Nissl-positive UL neurons were normalized across species to compare evolutionary variations in DL expansion. (**B**) DAPI nuclear staining of the adult mouse and rat cerebral cortex. Three rostral-to-caudal planes are defined relative to the hippocampus. White dotted lines delineate the boundary between UL and DL. (**C**) Total number of DAPI-labeled nuclei in the UL and DL across the three rostral-to-caudal levels in both species. Statistical significance (*P* < 0.05) is indicated by asterisks. (**D**) Immunohistochemical detection of projection neuron-specific markers (Satb2, Ctip2, and Tbr1). White dotted lines delineate the boundary between UL and DL. (**E**) Quantification of Satb2-, Ctip2-, and Tbr1-expressing neurons in the UL and DL of the mouse and rat primary somatosensory cortex (S1) at the adult stage. Data are presented as mean ± SEM (*n* = 3 samples per group); *P* values were calculated using Student’s *t* test. Scale bars: 500 μm (**A**), 100 μm (**B**, **D**).

To survey evolutionary variation in mammalian neocortical architecture, we first performed a histological comparison of adult somatosensory cortices across eight mammalian species (Fig. 1A; Appendix Fig. S1). A general pattern emerged: gyrencephalic species exhibited proportionally thicker UL, whereas lissencephalic rodents displayed a bias toward thicker DL, as reported previously (Fig. 1A; Appendix Fig. S1) (Charvet et al, 2017). Within the rodent lineage, however, we identified a notable divergence in the rat, which exhibits a significant bias toward DL thickening. This architecture stands in contrast to the human lineage, where cortical expansion is primarily attributed to enhanced UL production. While human cortical evolution has attracted intense interest, the specific adaptations in other clades remain underexplored. The DL expansion in rats thus provides a tractable and compelling model for studying the evolutionary plasticity of cortical structure.

To probe the developmental basis of these species differences, we performed detailed histological analyses in mice and rats. Nuclear staining revealed that while the overall laminar organization is conserved, rats possess a markedly thicker DL across all rostro-caudal regions (Fig. 1B; Appendix Fig. S3A,B). Quantitative assessments confirmed that rats harbor a higher absolute number of DL neurons than mice, whereas UL neuron numbers remain relatively conserved (Fig. 1C). We corroborated these findings using immunohistochemistry for subtype-specific markers: Satb2 (callosal projection neurons present in UL and a subset wev of DL; CPN), Ctip2 (corticospinal projection neurons, predominantly DL; CSPN), and Tbr1 (corticothalamic projection neurons, mainly DL; CThPN) (Fig. 1D,E). In the primary somatosensory cortex, delineated by the characteristic barrel structures (Appendix Fig. S3C,D), rats displayed significantly higher numbers of all three DL subtypes defined by Satb2, Ctip2, and Tbr1 than mice, while UL subtype counts were nearly identical between species or higher in mice. These DL-biased differences were already evident at an early postnatal stage P7 (Appendix Fig. S2A,B). The density of these neurons is highly comparable in the two species (Appendix Fig. S2C). Remarkably, consistent with this cellular expansion, the corticospinal tract exhibited a substantially greater cross-sectional difference between species than did the corpus callosum (Appendix Fig. S4). Together, these results demonstrate that species divergence between mice and rats is characterized chiefly by a robust expansion of DL neuron populations, a trait consistently observed throughout the cortical mantle.

### Individual cortical RG progenitors generate a species-specific number of DL neurons

**Figure EV1:**
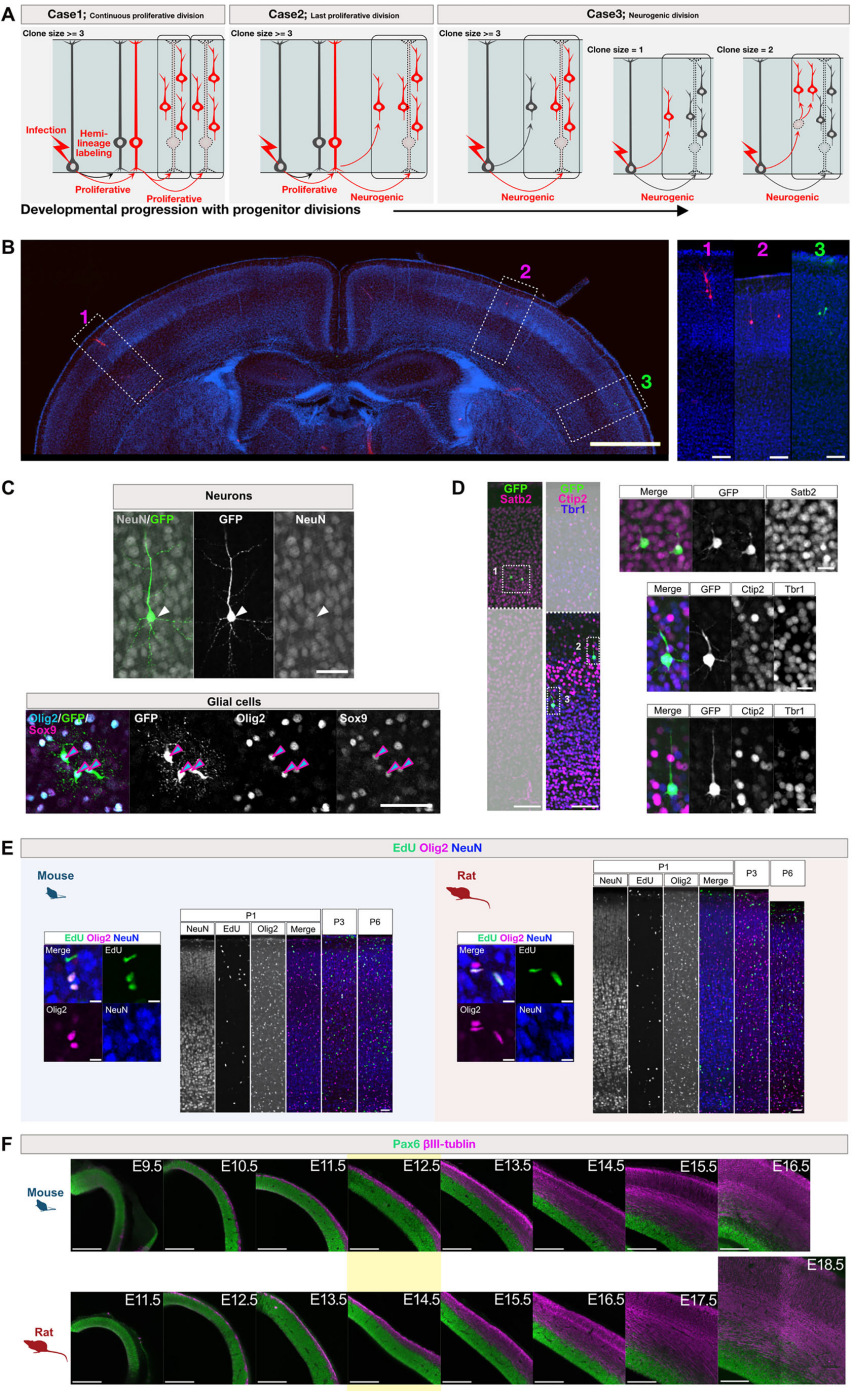
Clonal analysis of cortical RG progenitors in mice and rats from the onset to the end of excitatory neurogenesis, related to Fig. 2. (**A**) Schematic representation of hemi-lineage labeling via retroviral gene transfer, illustrating three scenarios of progenitor labeling. Cortical progenitors initially divide symmetrically to expand their population before transitioning to neurogenesis. Radial glial (RG) progenitors begin producing neurons by switching from symmetric proliferative divisions to asymmetric neurogenic divisions. Eventually, RG progenitors terminate producing neurons to begin gliogenesis. A clonal “unit” of neurons is defined as sister excitatory neurons that share a common progenitor origin and are produced during the first and subsequent neurogenic divisions of the mother progenitor (rounded boxes). Case 1: The retroviral vector infects a progenitor undergoing symmetric proliferative divisions. This results in multiple clonal units being labeled within the same cell cluster. Case 2: The retroviral vector infects an RG progenitor at its last proliferative division before initiating neurogenesis. In this ideal case, the entire clonal unit of sister neurons is labeled. Case 3: The retroviral vector infects an RG progenitor that has already started neurogenic divisions. In this scenario, some members of the clonal unit are missing from the labeled cohort, resulting in an incomplete clone. Alternatively, only one neuron (direct neurogenesis) or two neurons (indirect neurogenesis via intermediate progenitors) may be labeled. Given the heterogeneity in cortical progenitor division statuses, actual experimental data represent a mixture of these three cases. In Case 3, where neurogenic progenitors dominate, half of the clones are expected to consist of one or two neurons. Therefore, twice the proportion of one- or two-neuron clones serves as an estimate of the ratio of neurogenic progenitors at the time of retroviral injection. (**B**) Representative coronal section showing three labeled clones (RFP in magenta, GFP in green) with spatial separation and no overlap. (**C**) Representative images of neurons and glial cells. NeuN was used as a neuronal marker, whereas Olig2 and Sox9 were used to identify glial cells. Arrowheads denote GFP-positive cells co-expressing the indicated markers. (**D**) Representative clonal analysis following retroviral injection at E12.5 and tissue collection at P7. Cortical sections were immunostained for GFP together with the projection neuron markers Satb2, Ctip2, and Tbr1. Panels show the cortical location of GFP-labeled cells and their corresponding marker expression. (**E**) EdU labeling of postnatal (P1, P3, P6) mice and rats reveals co-labeling with Olig2 (glial progenitors) but not NeuN (neurons), suggesting no neurogenesis in the postnatal period in both species. (**F**) Neuron-progenitor ratio defined by Pax6 (green) and beta III tubulin (magenta) expression demonstrates developmental stage correspondence between species with a 2-day difference. E12.5 in mice and E14.5 in rats are the corresponding timing of onset of neurogenesis for the majority of cortical progenitors (yellow background). Scale bars: 1 mm (low magnification, (**B**), 100 μm (magnified images, (**B**), 50 μm (**C**), 200 μm (low magnification, (**D**), 20 μm (magnified images, (**D**), 50 μm (**E**), 200 μm (**F**).

**Figure 2:**
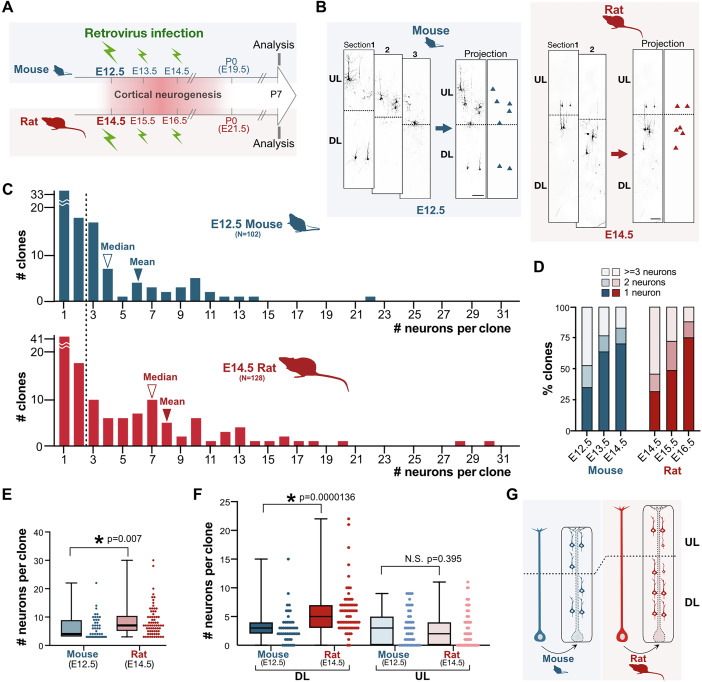
Clonal production of cortical excitatory neurons in mice and rats. (**A**) Time course of clonal analysis in the mouse and rat cerebral cortices. Retroviral injections were performed at the onset of neurogenesis (E12.5 in mice and E14.5 in rats), and animals were sacrificed at P7 for analysis. (**B**) Representative examples of cortical excitatory neuron clones in mice and rats. Scale bars: 100 μm. (**C**) Distribution of clone sizes when retroviruses were injected at the onset of neurogenesis. Clonal sizes represent the number of neurons generated by individual RG progenitors. Median and mean of clone size are indicated by open and filled arrowheads, respectively. (**D**) Percentage of clones containing one, two, or three or more neurons in mice and rats. (**E**) Comparison of clone size between species. Only clones with three or more neurons were analyzed. *n* mouse_E12.5 = 47 clones, *n* rat_E14.5 = 69 clones. (**F**) Distributions of upper layer (UL) and deep layer (DL) neurons per clone. Rats exhibit a marked increase in the number of DL neurons per clone compared to mice, while UL neuron numbers remain similar between the species, *n* mouse_E12.5 = 47 clones, *n* rat_E14.5 = 69 clones. (**G**) Schematic diagram summarizing the clonal analysis results (mean number of neurons per clone), highlighting species-specific differences in DL neuron production. For box plots in (**E**, **F**), the center line indicates the median (50th percentile), the bounds of the box indicate the 25th and 75th percentiles, and the whiskers extend to the minimum and maximum values. Statistical analysis was performed using Welch’s *t* test, with detailed results available in Dataset EV1. Asterisks indicate statistical significance (*P* < 0.05).

To investigate the developmental basis of the species-specific neuronal balance, we next scrutinized the neurogenic process at the single-progenitor level. In the neocortex, excitatory neurons are locally supplied by RG progenitors, and their clonally related progeny are typically organized as radial clusters within a columnar space. While previous studies using Mosaic analysis with double markers (MADM) have shown that individual mouse RG progenitors generate an average of 8–9 neurons, with 40–50% contributing to DL subtypes (Llorca et al, 2019; Gao et al, 2014), a direct quantitative comparison between species requires an equivalent experimental platform. To this end, we employed “hemi-lineage” labeling using retroviruses expressing fluorescent reporters (Llorca et al, 2019) (See details for hemi-lineage labeling in Fig. EV1A,B). This approach ensures that a single viral integration into the host genome permanently labels the progeny derived from one daughter cell. By optimizing retroviral titers for GFP and RFP, we performed sparse labeling at embryonic stages and analyzed the clones at postnatal day 7 (P7), when cortical neurogenesis is complete (Fig. EV1E). GFP- and RFP-positive cells were sparsely distributed across cortical layers without overlap between clones, confirming spatial separation of labeled clones (Figs. 2A,B and EV1). Labeled neurons were identified based on their characteristic morphology and laminar position, and their identity was further validated by molecular marker expression (Fig. EV1C,D). Only neurons are considered in the following clonal analyses.

To compare the entire neurogenic program between species, we first determined the precise timing of the transition from the proliferative to the neurogenic state. Given that the progenitor population is heterogeneous, we quantified the percentage of clones containing one or two neurons—an index of neurogenic progenitors at the time of infection (Fig. EV1A). In mice, retroviral injection at E12.5 revealed that nearly half of the clones were already neurogenic, consistent with previous reports (Llorca et al, 2019) (Fig. 2C,D). Remarkably, a highly comparable neurogenic onset was observed in rats when the injection was performed at E14.5 (Fig. 2D). In the rat, neurogenic division became dominant by E15.5–E16.5, with over 50% of clones comprising one or two neurons (Fig. 2D). These findings establish that E14.5 in rats chronologically corresponds to E12.5 in mice as the functional onset of cortical neurogenesis, a correspondence further corroborated by the conserved neuron-to-progenitor ratios observed in our histological analyses (Fig. EV1F).

Having established this temporal baseline, we compared the neuronal output of individual progenitors from the onset of neurogenesis through P7 (Fig. 2B,C). We focused our analysis on clones with three or more neurons to ensure the inclusion of robust lineage. In mice, the mean clonal size was 6.22 ± 3.88 (median 4), aligning with previous data (Llorca et al, 2019) (Fig. 2C,E). In striking contrast, the clonal distribution in rats was skewed toward larger sizes, with a significantly higher mean of 8.05 ± 5.44 (median 7) (Fig. 2C,E). When categorized by somal position, rats exhibited a disproportionately larger number of DL neurons per clone (5.90 ± 4.01 in rats vs. 3.04 ± 2.69 in mice), whereas the number of UL neurons remained remarkably similar between the two species (2.62 ± 2.98 in rats vs. 3.11 ± 2.95 in mice) (Fig. 2F). These results demonstrate that while both species utilize multipotent RG progenitors capable of generating both DL and UL subtypes, the intrinsic neurogenic activity of individual progenitors is specifically amplified in the rat lineage to favor DL expansion (Fig. 2G).

### Species-specific timeline of cortical neurogenesis: an extended early neurogenic phase in rats

**Figure 3:**
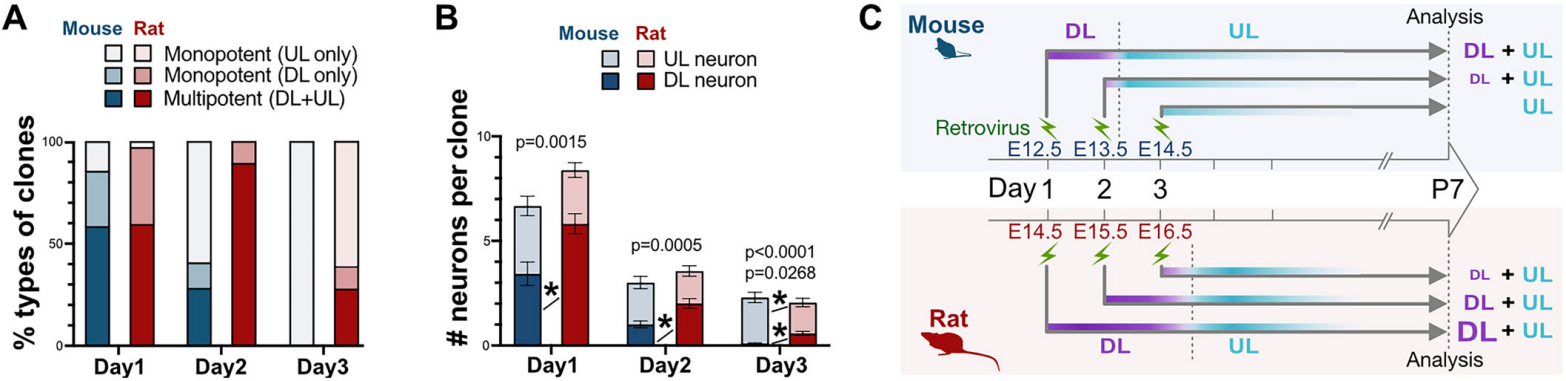
Temporal progression of cortical neurogenesis revealed by retroviral labeling of RG progenitors at distinct injection timings. (**A**) Clones were categorized into three types: multipotent (DL + UL), monopotent (DL only), and monopotent (UL only). By Day 3 of neurogenesis, all mouse clones were UL-only monopotent type, whereas a significant proportion of rat clones remained multipotent when retroviruses were injected at the corresponding stage. (**B**) Number of DL and UL neurons per clone after retrovirus injection at Days 1, 2, and 3 of neurogenesis in mice and rats. Rat clones contain DL neurons even at Day 3, unlike mouse clones, which transitioned entirely to UL neurons. Data are presented as mean ± SEM. *P* values in DL neuron: Pmouse-rat day 1 = 1.50 × 10^−3^, Pmouse-rat day 2 = 5.01 × 10^−4^, Pmouse-rat day 3 = 9.80 × 10^−5^; *P* values in UL neuron: Pmouse-rat day 3 = 2.68 × 10^−2^. Number of clones: *n* mouse_Day1 = 47 clones, *n* mouse_Day2 = 68 clones, *n* mouse_Day3 = 70 clones, *n* rat_Day1 = 69 clones, *n* rat_Day2 = 50 clones, *n* rat_Day3 = 75 clones. (**C**) Summary illustration of clonal analysis with retroviral injections at different time points. Vertical dotted lines indicate the expected timing of the DL-to-UL subtype transition for the majority of RG progenitors in mice and rats. Data are presented as the mean ± standard error of the mean (SEM). Statistical analysis was performed using Welch’s *t* test, with detailed results available in Dataset EV1. Asterisks indicate statistical significance (*P* < 0.05).

To elucidate the mechanism underlying the species-specific dosage of DL neuron production, we examined the temporal progression of neurogenesis through sequential clonal labeling (Figs. 2A and 3; Appendix Fig. S5). Retroviruses were injected at the onset of neurogenesis (“Day 1”) and at 24-hour (“Day 2”) and 48-hour (“Day 3”) intervals, with progeny analyzed at P7 (Fig. 3A). While the overall pattern of neurogenesis remained consistent between species, a profound divergence emerged in their temporal dynamics. In both species, the proportion of monopotent clones containing only UL neurons increased progressively from Day 1 to Day 3, reflecting the canonical inside-out sequence of cortical neurogenesis. However, a clear species-specific difference was observed: mice exhibited a markedly higher percentage of UL-only clones in injections on Days 2 and 3, whereas rats retained a significantly greater proportion of multipotent clones capable of generating DL neurons at these same stages (Fig. 3A). This difference was further underscored by the mean number of DL neurons per clone; in rats, DL production persisted even in Day 3 injections, whereas in mice, DL neurons were virtually absent in the corresponding condition (Fig. 3A,B). These observations demonstrate that cortical neurogenesis follows distinct temporal programs in the two species. Specifically, in rats, the early neurogenic phase for DL production is intrinsically extended beyond Day 3, while mice complete the transition to UL production by this stage (Fig. 3C).

**Figure 4:**
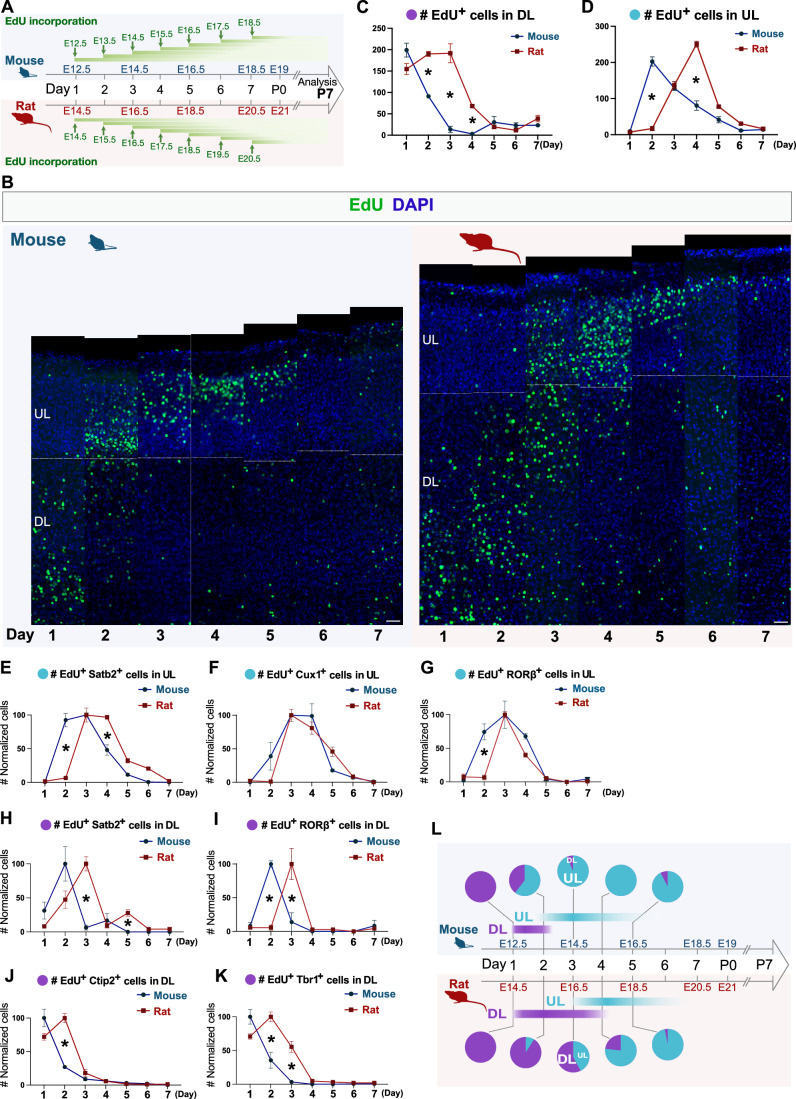
Birthdating of cortical excitatory neurons in mice and rats. (**A**) Experimental design for birthdating analysis in mice and rats, showing different timing of EdU injections from the onset until the end of neurogenesis. (**B**) Representative images of cortical columns in S1 with EdU-labeled neurons at the specified developmental stages. Scale bars: 50 μm. (**C**, **D**) Quantification of DL (**C**) and UL subtypes (**D**) labeled with EdU at different stages. Asterisks indicate statistical significance (*P* < 0.05). *P* values in DL: Pmouse-rat day 2 = 1.60 × 10^−5^, Pmouse-rat day 3 = 1.56 × 10^−3^, Pmouse-rat day 4 = 5.30 × 10^−5^, in UL: Pmouse-rat day 2 = 2.84 × 10^−3^, Pmouse-rat day 4 = 9.61 × 10^−4^. (**E**–**K**) Normalized counts of neurons co-labeled for EdU and projection-specific markers, relative to the peak of EdU+ and marker+ cells. (**E**) Satb2-positive UL neurons, Pmouse-rat day 2 = 8.29 × 10^−4^, Pmouse-rat day 4 = 4.03 × 10^−3^, (**F**) Cux1-positive UL neurons, (**G**) RORβ-positive neurons located in UL, Pmouse-rat day 2 = 2.09 × 10^−2^, (**H**) Satb2-positive DL neurons, Pmouse-rat day 3 = 6.87 × 10^−3^, Pmouse-rat day 5 = 2.76 × 10^−2^, (**I**) RORβ-positive DL neurons, Pmouse-rat day 2 = 1.48 × 10^−4^, Pmouse-rat day 3 = 4.35 × 10^−2^, (**J**) Ctip2-positive DL neurons, Pmouse-rat day 2 = 5.76 × 10^−3^ and (**K**) Tbr1-positive DL neurons, Pmouse-rat day 2 = 1.53 × 10^−2^, Pmouse-rat day 3 = 2.49 × 10^−2^. (**L**) Summary of neuronal birthdating in mice and rats, highlighting species-specific differences in the timing of DL and UL neuron production. Data are presented as the mean ± standard error of the mean (SEM). *n* = 3 biologically independent samples per group in (**C**, **D**, **E**–**K**). Asterisks indicate statistical significance (*P* < 0.05). Statistical analysis was performed using Welch’s *t* test, with detailed results available in Dataset EV1.

To directly examine the temporal dynamics of cortical neuron subtype generation, we performed a birthdating analysis using the nucleotide analog 5-ethynyl-2’-deoxyuridine (EdU) (Fig. 4A). EdU was administered at specific developmental stages, from the onset of neurogenesis (Day 1; E12.5 in mice, E14.5 in rats) through one day prior to delivery (Day 7; E18.5 in mice, E20.5 in rats). Analysis of layer positions and marker expression at P7 revealed a striking species-specific divergence in the neurogenic timeline. In mice, DL neurons were generated within a brief two-day window (Days 1–2) before the transition to UL production. In stark contrast, DL neuron production in rats was significantly prolonged, spanning a 4-day period (Days 1–4) (Fig. 4B–D). Remarkably, the subsequent duration of UL neurogenesis was comparable between the two species, lasting 4 days until termination. This temporal shift was further corroborated by the quantification of subtype-specific markers (Fig. 4E–K; Appendix Fig. S6). These results demonstrate that the conserved inside-out program of cortical neurogenesis is not uniformly scaled, but rather undergoes species-specific fine-tuning during the early neurogenic phase. This non-uniform adaptation is characterized by an extended duration of DL neuron production in the rat, which precedes the eventual transition to the UL program (Fig. 4L).

### Species-specific timing of DL to UL transition corroborated by axonal projection analysis

**Figure EV2:**
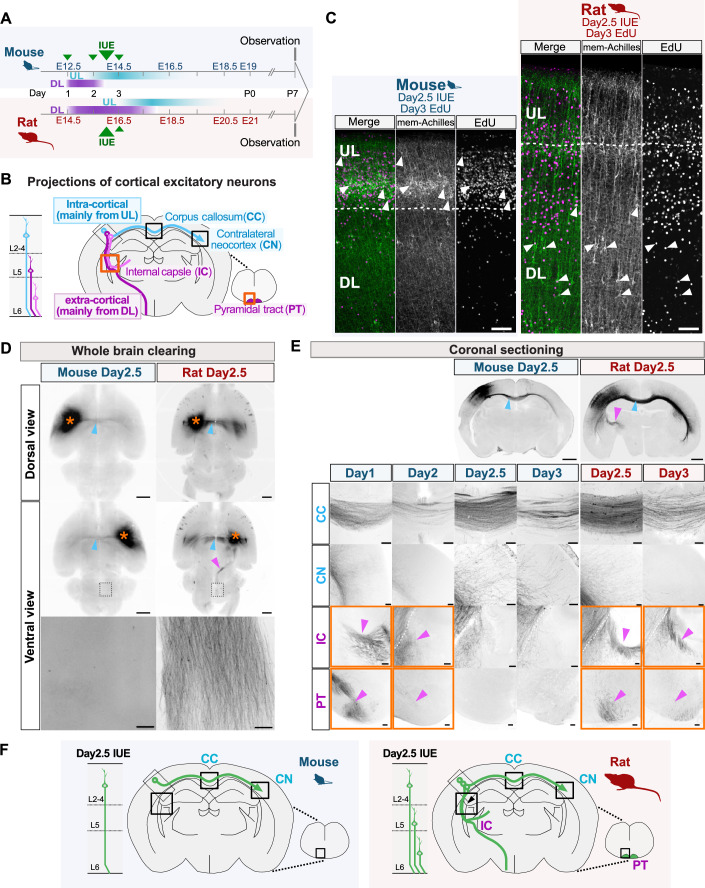
Projection identity of neurons generated at Day 2.5–3 of neurogenesis in mice and rats, related to Fig. 4. (**A**) Schematic of IUE timing and sacrifice for axon projection analysis. (**B**) Major projection targets of cortical excitatory neurons: corpus callosum (CC), contralateral neocortex (CN), internal capsule (IC), and pyramidal tract (PT). (**C**) The neurons electroporated at Day 2.5 are co-labeled with EdU injected at Day 3 in both species. (**D**) Tissue-cleared whole-brain images (dorsal and ventral views) show distinct projection patterns. In rats, prominent ipsilateral projections to the brainstem (magenta arrowheads) are unique. (**E**) Coronal sections confirm unique descending projections in rats and their absence in mice after Day 3 IUE. (**F**) Summary illustration of projection differences between mouse and rat neurons generated at Days 2.5–3. Scale bars: 1 mm (low magnification, (**C**, **D**), 100 μm (magnified images, (**D**, **E**).

To further confirm the species-specific timing of the DL to UL subtype switch, we examined the axonal projection patterns of neurons generated during the mid-neurogenic phase (Days 2.5 and 3). Given that axonal trajectories represent the defining feature of these neuronal subtypes, we sought to verify whether the temporal shifts in somal location and marker expression (Figs. 3 and  4) are indeed reflected in their connectivity. We analyzed the projections of neurons generated at specific timings using in utero electroporation (IUE) of a plasmid encoding the membrane-anchored fluorescent protein Achilles, which allowed for precise trajectory tracing at P7 (Fig. EV2A,B). The laminar distribution of neurons labeled at Day 2.5 closely matched the results of EdU pulse-labeling at Day 3, the stage exhibiting the most pronounced species-specific divergence (Fig. EV2C). By combining tissue clearing for whole-brain visualization (Fig. EV2D) with immunostaining in slices (Fig. EV2E), we examined the axonal targets. In mice, neurons labeled at this mid-neurogenic stage predominantly projected to the corpus callosum. In striking contrast, neurons in rats generated at the same stage projected to both intra- and extracortical targets, including the corpus callosum, thalamus, and descending brainstem structures such as the ipsilateral pyramidal tract (Fig. EV2D–F). Consistent with previous studies (Greig et al, 2013), neurons generated at Days 1–1.5 in mice projected to brainstem targets, indicating that the DL to UL transition occurs between Days 1.5 and 2 in this species (Fig. EV2E). These findings align with our birthdating results, where mid-neurogenic neurons (Day 3) in mice are predominantly Satb2-positive CPN in layers 2/3, whereas in rats, they include a significant population of Tbr1-positive CThPN and Ctip2-positive CSPN in layers 5–6. These observations demonstrate that the mid-neurogenic phase produces distinct neuronal ensembles in mice and rats, confirming that the species-specific timing of the DL to UL subtype switch is fundamentally encoded in their axonal targeting programs (Fig. EV2F).

### Conserved progenitor kinetics and a modulated contribution of indirect neurogenesis

**Figure 5:**
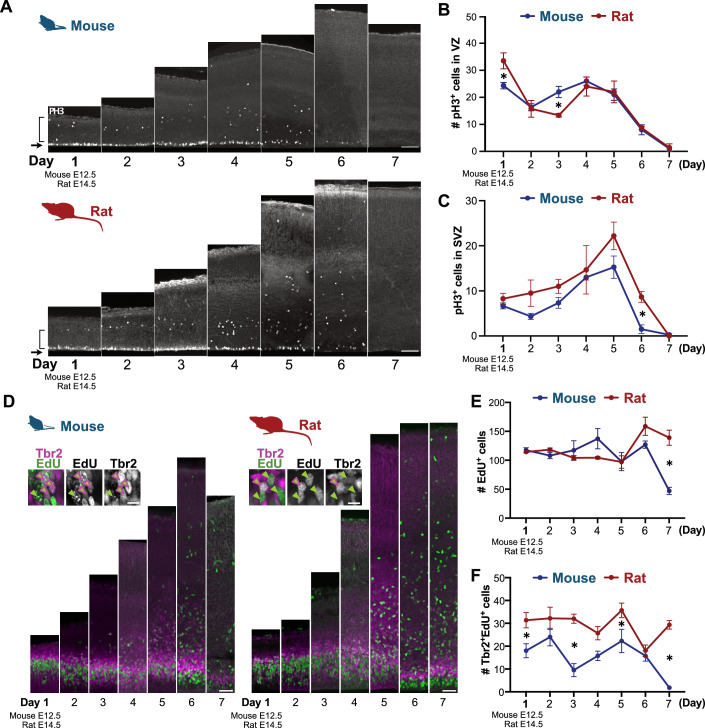
Dynamics of progenitor cell cycle and indirect neurogenesis activity in mouse and rat cortical neurogenesis. (**A**–**C**) Mitotic progenitors in the cortical neurogenesis of mice and rats (Days 1–7) are immunolabeled with phospho-histone H3 antibody. Mitotic RG and IP are separately quantified in the ventricular zone (VZ, arrows in (**A**); quantification in (**B**) and in the subventricular zone (SVZ, parentheses in (**A**); quantification in (**C**). *P* values in (**B**): Pmouse-rat day 1 = 4.66 × 10^−2^, Pmouse-rat day 3 = 4.25 × 10^−2^; *P* values in (**C**): Pmouse-rat day 6 = 8.43 × 10^−3^. (**D**) Cells in the G2/S phase are labeled with EdU administered one hour before sacrifice. (**E**) Total number of EdU-labeled cells during the neurogenic period is quantified, Pmouse-rat day 7 = 8.02 × 10^−3^. (**F**) Double-labeled EdU and Tbr2-positive cells, identified as IP, are quantified across neurogenic stages, Pmouse-rat day 1 = 4.66 × 10^−2^, Pmouse-rat day 3 = 4.25 × 10^−2^, Pmouse-rat day 1 = 4.66 × 10^−2^, Pmouse-rat day 3 = 4.25 × 10^−2^. Data are presented as the mean ± standard error of the mean (SEM). *n *= 3–6 biologically independent samples per group in (**B**, **C**, **E**, **F**). Asterisks indicate statistical significance (*P* < 0.05). Statistical analysis was performed using Welch’s *t* test, with detailed results available in Dataset EV1.

We next examined whether the enhanced production of DL neurons in rats stems from a transient acceleration of progenitor cycling or an increased contribution of indirect neurogenesis (Mihalas et al, 2016; Hevner, 2019; Hatanaka et al, 2024; Kowalczyk et al, 2009). To explore these possibilities, we first analyzed the density of mitotic progenitors at the ventricular surface and in the adventricular region of the future primary somatosensory field (Fig. 5A–C). While the temporal dynamics of mitotic density were conserved between the two species, subtle yet discernible species-specific signatures emerged. Specifically, rats exhibited a higher ratio of mitotic cells in the VZ at the onset of neurogenesis, the precise period when DL subtypes are generated in both species. Further assessment of G2/S-phase cells via pulse-EdU labeling (Fig. 5D,E) and direct measurement of cell cycle length through time-lapse imaging of primary RG progenitors (Appendix Fig. S7) revealed that progenitor kinetics are highly conserved between species. These findings indicate that the fundamental pace of the cell cycle remains largely equivalent in mice and rats, consistent with previous reports (Miller and Kuhn, 1995; Takahashi et al, 1996).

Despite this overall conservation, we noted a minor increase in SVZ mitosis in rats compared to mice (Fig. 5C). While the total abundance of Tbr2-positive cells was nearly indistinguishable between species (Appendix Fig. S8), rats displayed a consistently higher proportion of IPs in the G2/S phase (EdU/Tbr2 double-positive) throughout the neurogenic period (Fig. 5F). This observation suggests that indirect neurogenesis plays a more prominent role in the rat lineage, extending its influence across the entire neurogenic program. Together, these results demonstrate that in addition to the extended duration of the DL production window (Figs. 3 and  4), subtle modulations in cell cycle kinetics and the amplification of indirect neurogenesis collaboratively contribute to the species-specific dosage of DL neurons.

### Non-uniform temporal progression of cortical RG progenitors revealed by scRNAseq

**Figure 6:**
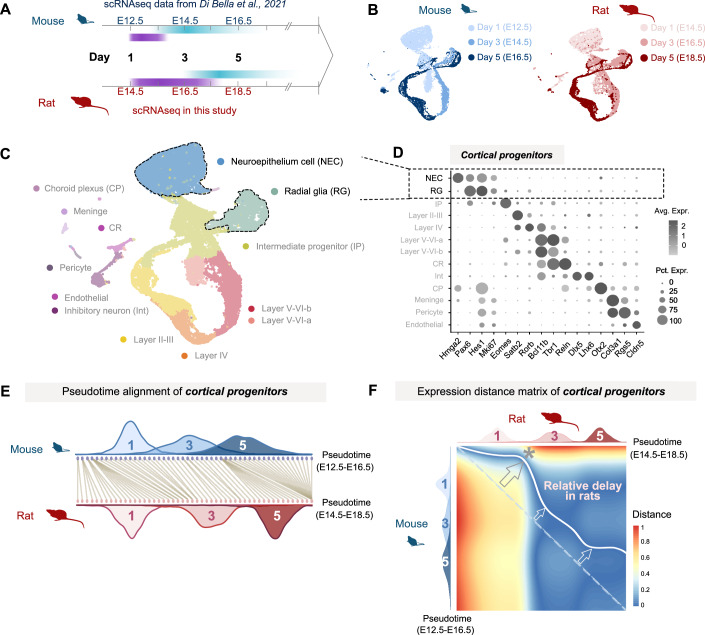
Temporal progression of progenitor aging in the mouse and rat cortical neurogenesis revealed by scRNAseq. (**A**) scRNAseq analysis of rat cortical cells collected at three neurogenic stages (Days 1, 3, and 5) was performed and compared with previously published mouse datasets from corresponding stages. (**B**) UMAP visualization of cortical cells from three developmental stages in mice and rats. (**C**) UMAP representation of cortical cells from both species, illustrating the clustering of major cell types. (**D**) Cell type-specific marker genes used for annotation, ensuring consistent identification across species. (**E**) Alignment of mouse and rat progenitor cells based on their pseudotime scores. Trajectories in each species were divided into 50 blocks (“Pseudocells”) for pairwise distance calculations using dynamic time warping. Histograms display the distribution of cells from sampling stages (Days 1, 3, and 5) along the trajectories. (**F**) Distance matrix of mouse and rat progenitor cells based on “progenitor age gene” expression. Cells were aligned by pseudotime scores along species-specific trajectories. The optimal alignment is indicated by the solid white line, which deviates from the diagonal dotted line, reflecting delayed developmental progression in rats compared to mice. Inflection points (open arrows) highlight significant changes in relative developmental tempo, particularly in the early phase (asterisk).

To investigate the molecular basis of the species-specific temporal dynamics, particularly the protracted DL production in rats, we performed a comparative scRNAseq analysis of neurogenic progenitors. Utilizing the Chromium X system, we obtained high-quality transcriptomic profiles for 14,290 cells from rat cortices at Days 1 (E14.5), 3 (E16.5), and 5 (E18.5) of neurogenesis (Fig. 6A). After quality control, we integrated these profiles with previously published mouse cortical cell data from corresponding neurogenic stages, which is prepared using the consistent platform with ours (Di Bella et al, 2021) (Appendix Fig. S9). Uniform Manifold Approximation and Projection (UMAP) (McInnes et al, 2018; Becht et al, 2018) identified 16 distinct cell clusters, each annotated with canonical marker genes (Fig. 6B,C; Appendix Fig. S9C,D). All major cortical cell types were represented, with no cluster dominated by a single species or developmental stage, indicating that clustering was driven by biological cell-type identity rather than technical artifacts (Fig. 6C,D).

We next examined the temporal gene expression changes within the neuroepithelial cells (NEC) and RG progenitors, as neuronal laminar fate is fundamentally determined by the progenitor’s developmental age. Using a machine learning-based ordinal regression model (Telley et al, 2019), we identified stage-specific “progenitor age genes” (Dataset EV2 and Appendix Fig. S10A–F), which showed significant overlap between species and with previously identified markers of cortical neurogenesis (Telley et al, 2016; Nowakowski et al, 2017). Gene ontology (GO) analysis enriched for terms such as “positive regulation of Wnt signaling pathway” (Appendix Fig. S10G). To quantitatively compare the temporal trajectories between species, we aligned progenitors by developmental age using a pseudotime analysis based on these age-related expression profiles (cellAlign; Fig. 6E). By minimizing expression distance, we identified corresponding pairs of mouse and rat progenitors. Notably, this analysis revealed that most rat progenitors at Day 1 correspond to a smaller subset of mouse progenitors with the youngest pseudotime scores. This observation indicates that rat progenitors retain early-age expression profiles longer than their mouse counterparts, which progress through developmental aging more rapidly.

In contrast, the subsequent neurogenic phase progressed in parallel (Days 1 to 3 in mice; Days 3 to 5 in rats), with some divergence toward the end of neurogenesis. A quantitative measurement of expression distance revealed a non-linear correspondence in pseudotime, with the most significant delay in rats occurring where mouse Day 1 and rat Day 3 progenitors overlapped (asterisk in Fig. 6F). Importantly, while this analysis identified stage pairs with the highest similarity, the correspondence at the edges of the temporal sampling window is inherently less certain than at the center. Consequently, we focus on the notable reflection point at the center of our dataset. This protracted progression in rats was further validated using an independent mouse scRNAseq dataset (43) (Appendix Fig. S11A). In summary, single-cell transcriptomics demonstrates that the temporal progression of cortical progenitors undergoes species-specific, non-uniform scaling, fundamentally shaping the neuronal output.

### Wnt ligand genes exhibit protracted expression in rat progenitor cells

**Figure 7:**
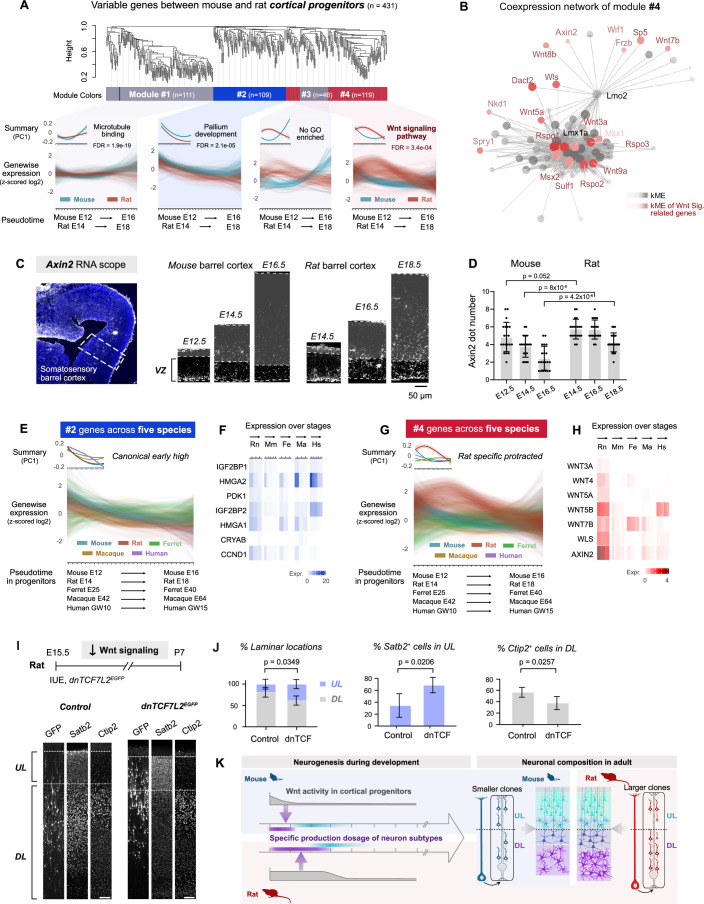
Protracted expression of Wnt ligand genes in rat cortical progenitors. (**A**) Weighted gene co-expression network analysis (WGCNA) of 431 highly variable genes in mouse and rat RG progenitors identifies four co-expression modules. Gene expression values were log-normalized and z-scored across pseudotime to show temporal dynamics (see Methods). Weighted expression summary (first principal component) and the top Gene Ontology (GO) term (lowest FDR-adjusted *P* value) are indicated in the upper left and right of each plot, respectively. (**B**) Co-expression network for module 4. The top 300 connections are shown, with edge shading representing module membership (kME) values. Genes associated with the Wnt signaling pathway are highlighted in pink to red based on kME. (**C**, **D**) RNAscope in situ hybridization for *Axin2* on coronal sections of the somatosensory cortex barrel field in mice (E12.5, E14.5, and E16.5) and rats (E14.5, E16.5, and E18.5). *Axin2* expression quantified as dots per cell in the ventricular zone (*n* = 20 cells). Welch’s corrected *t* test. Data are presented as the mean ± standard error of the mean (SEM). (**E**, **F**) Expression dynamics of the module #2 “canonical” early-high module genes (*n* = 109) across developing somatosensory cortex progenitors in five mammalian species. (**E**) Log-normalized and z-scored expression along the pseudotime axis. (**F**) Absolute expression values across actual developmental stages in each species. (**G**, **H**) Expression dynamics of the module #4 “rat-protracted” early-high module genes (*n* = 111) across five mammalian species, as in (**C**, **D**). (**I**, **J**) In utero electroporation of rat E15.5 cortices with dominant-negative TCF7L2-IRES-EGFP (dnTCF) or EGFP control to perturb Wnt signaling in vivo. Rats sacrificed at P7 (*n* = 4 control, *n* = 3 dnTCF) with laminar distribution of labeled cells and projection neuron markers (Satb2+ and Ctip2 + ) examined and quantified. Welch’s corrected *t* test. Data are presented as the mean ± standard error of the mean (SEM). (**K**) Schematic illustration summarizing this study.

To elucidate the molecular logic underlying the protracted DL production in rats, we performed an unbiased re-analysis of our scRNAseq dataset. Weighted Gene Co-expression Network Analysis (WGCNA) of 431 highly variable genes in RG resolved four distinct temporal modules (Fig. 7A; Dataset EV2). While Modules 1 and 2 exhibited conserved trajectories across species, Modules 3 and 4 displayed striking divergence. Notably, Module 4 genes, which showed a steady decline in mice, remained elevated during the early neurogenic phase in rats, mirroring the species-specific timing of DL production. GO enrichment analysis associated Module 4 with the Wnt signaling pathway, while Module 2 genes, which were conserved, were linked to pallial development. These findings suggest that while the fundamental pace of progenitor aging is conserved (Modules 1 and 2), the rat-specific extension of the early neurogenic window is uniquely driven by the sustained activity of Wnt-related genes.

Canonical Wnt signaling is a key regulator of the temporal program of corticogenesis and is elevated early, declining as development proceeds in both mouse and human (Nowakowski et al, 2017; Telley et al, 2019). Experimentally increasing Wnt signaling in RG biases production toward DL subtypes, whereas reducing it shifts fate toward UL subtypes in mice (Munji et al, 2011; Vitali et al, 2018; Oberst et al, 2019; Wrobel et al, 2007). Given the necessity and sufficiency of Wnt signaling for time-dependent subtype specification, we interrogated module 4 in detail. Its co-expression network contained transcription factors, multiple Wnt ligands, and pathway components such as *Axin2* and *Wls*, but notably lacked canonical Wnt receptor genes (Fig. 7B). Fluorescent in situ hybridization using RNAscope confirmed significantly elevated *Axin2* expression in rat RG throughout neurogenesis compared with mouse, consistent with our scRNAseq results (Fig. 7C,D). Because *Axin2*, a standard readout of Wnt activity, displayed divergent dynamics between species, we hypothesized that species-specific Wnt kinetics underlie the different durations of DL neurogenesis.

Canonical Wnt signaling is a pivotal regulator of cortical temporal identity, with its early elevation being essential for DL specification (Munji et al, 2011; Vitali et al, 2018; Oberst et al, 2019; Wrobel et al, 2007). We therefore investigated the Module 4 network, identifying multiple Wnt ligands and downstream components such as *Axin2* and *Wls* (Fig. 7B). Consistent with our transcriptomic data, quantitative RNAscope analysis confirmed significantly elevated *Axin2* expression in rat RG throughout the neurogenic period (Fig. 7C,D). To determine whether this rat-like pattern is ancestral, we integrated single-cell datasets from human, macaque, and ferret (Appendix Fig. S11B) (Wang et al, 2025; Micali et al, 2023; Bilgic et al, 2023). Remarkably, while Module 2 genes (e.g., *Hmga2*, *Igf2bp2*) declined consistently across all five species (Fig. 7E,F), the sustained expression of Wnt ligands and *Axin2* was observed exclusively in the rat (Fig. 7G,H; Appendix Fig. S12). This suggests that the rat lineage underwent a unique evolutionary shift, deviating from the ancestral program of sharp Wnt decline.

**Figure EV3:**
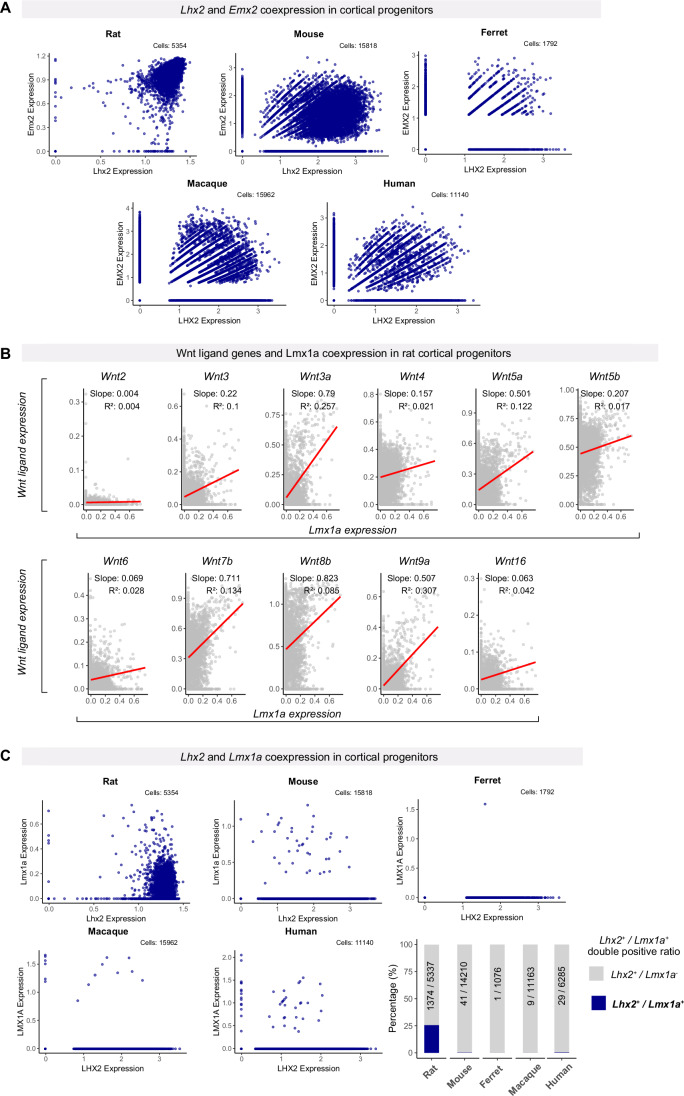
Gene co-expression patterns in cortical progenitors across five mammalian species, related to Fig. 7. (**A**) Co-expression patterns of *Emx2* and *Lhx2* in cortical progenitors across five mammalian species (rat, mouse, ferret, macaque, and human). Expression values were retrieved from the SCT assay in each regressed object. (**B**) Co-expression patterns of *Lmx1a* and *Lhx2* in cortical progenitors across five mammalian species (rat, mouse, ferret, macaque, and human). Expression values were retrieved from the SCT assay in each regressed object. The right bottom panel is the double-positive ratio of *Lhx2* and *Lmx1a* in cortical progenitors across five mammalian species. Cells with gene expression values above 0.1 for a given gene were defined as positive for that gene. (**C**) Co-expression of Wnt ligand genes (*n* = 11) with Lmx1a in rat cortical progenitors. Gene expression data retrieved from SCT assay.

**Figure EV4:**
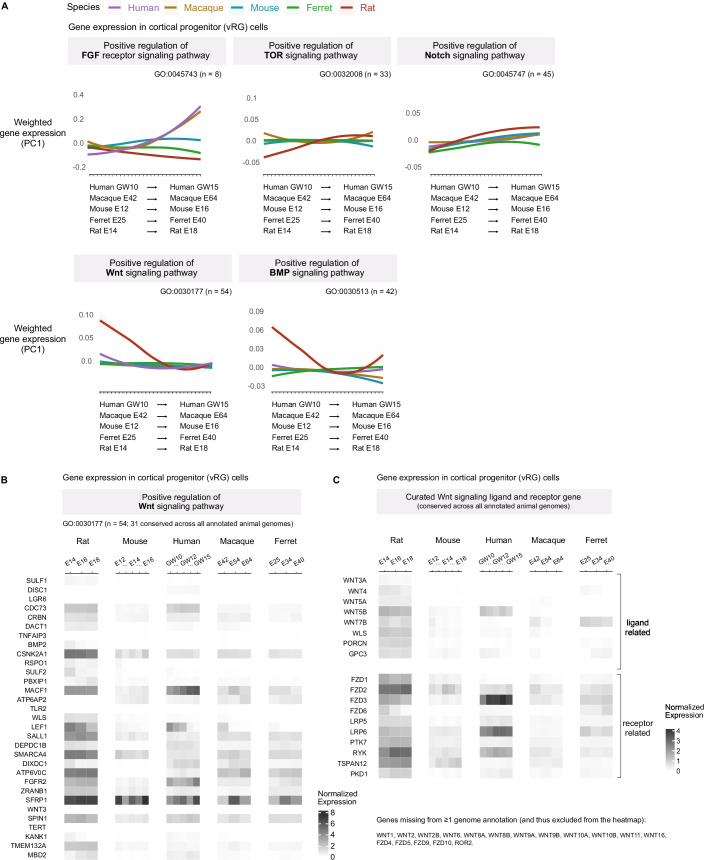
Major signaling pathways regulating brain development in cortical progenitors of five mammalian species, related to Fig. 7. (**A**) Weighted summary (PC1) of gene expression for involved genes, retrieved from the corresponding Gene Ontology term (positive regulation only). Ventricular radial glia (vRG) from equivalent developmental stages in human (GW10–GW15), macaque (E42–E64), mouse (E12–E16), ferret (E25–E40), and rat (E14–E18) were analyzed. (**B**) Heatmap of gene expression in cortical progenitors, highlighting genes from “Positive regulation of Wnt signaling pathway” (GO:0030177). (C) Heatmap of gene expression in cortical progenitors of curated Wnt signaling-related ligand and receptor genes. Genes not conserved across all annotated animal genomes were excluded from the heatmap and are listed below it.

**Figure EV5:**
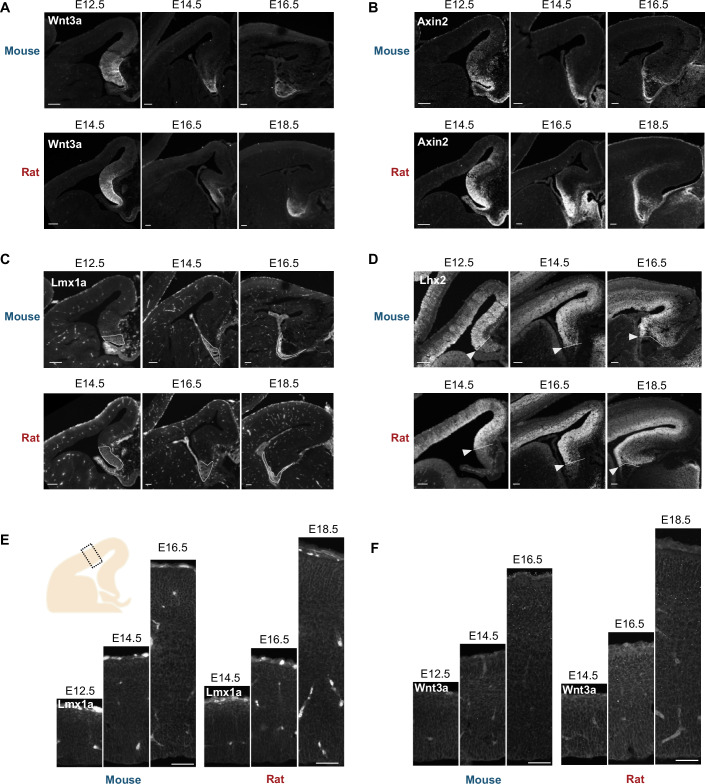
Species-specific temporal dynamics of cortical hem-associated Wnt signaling during corticogenesis, related to Fig. 7. (**A**) Immunostaining for Wnt3a in coronal sections of developing mouse and rat cortices at the indicated embryonic stages. (**B**) In situ hybridization of Axin2, a canonical Wnt signaling target gene, in mouse and rat cortices across developmental stages. (**C**) In situ hybridization of Lmx1a, a cortical hem marker, in mouse and rat embryos at corresponding stages. Outlined regions indicate the cortical hem. (**D**) Immunostaining for Lhx2, a transcription factor expressed in cortical neuroepithelium and excluded from the cortical hem. Arrowheads indicate the hem–cortex boundary. (**E**, **F**) Representative images of Lmx1a (**E**) and Wnt3a (**F**) expression in the dorsomedial cortex of mouse and rat embryos at the indicated developmental stages. Comparable cortical regions were analyzed. Scale bars: 100 μm (**A**–**D**), 50 μm (**E**, **F**).

We further examined the regulatory mechanisms sustaining this rat-specific Wnt activity. First, we found that Wnt receptor genes exhibited no consistent temporal changes across any of the species examined (Appendix Fig. S13A). Second, the elevated expression of Wnt ligands in rats was uniquely confined to RG and was absent in other cell types, including IP (Appendix Fig. S13B). Third, we identified *Lmx1a*, a master regulator of the cortical hem, a signaling center that secretes multiple Wnt ligands, to be expressed weakly but significantly in rat RG. This expression was observed exclusively in the rat lineage among the five species compared (Fig. EV3A). Notably, we found a clear positive correlation between *Lmx1a* and Wnt ligand expression in rat RG, suggesting a co-option of the hem-specific *Lmx1a*-Wnt cascade (Fig. EV3B). Intriguingly, a significant proportion of rat RG progenitors co-expressed *Lmx1a* and *Lhx2*, whereas these genes showed minimal co-expression in other species (Fig. EV3C). Given that *Lmx1a* and *Lhx2* function as mutually exclusive selector genes for the hem and the neocortex, respectively, in mice, their convergence in rat RG suggests a novel mechanism for the ectopic induction of Wnt ligands in neocortical progenitors. Fourth, among the five major signaling pathways (FGF, mTOR, Notch, Wnt, and BMP), genes related to BMP signaling—another hallmark of the cortical hem—were also uniquely upregulated in rats (Figs. EV4 and  EV5). These findings collectively support the hypothesis that a gene regulatory network intrinsically characteristic of the cortical hem is ectopically induced in rat RG, maintaining population-wide Wnt signaling through auto- and paracrine mechanisms to extend the DL neurogenic window (Figs. 1A and  7K).

Finally, to validate the functional significance of this sustained Wnt signaling, we attenuated the pathway by introducing a dominant-negative form of TCF7L2 (dnTCF) via in utero electroporation at E15.5. This manipulation significantly shifted the neuronal balance toward UL fates by E20.5 (Appendix Fig. S14) or P7 (Fig. 7I,J). Specifically, the proportion of DL neurons decreased from 81.2% in controls to 61.2% following dnTCF expression, a trend corroborated by Satb2 and Ctip2 immunostaining. These results demonstrate that the sustained production of DL subtypes in the rat is fundamentally dependent on the elevated Wnt signaling intrinsically maintained in RG during the mid-neurogenic period.

## Discussion

In this study, we performed a comparative anatomical analysis of layer architecture within the primary somatosensory cortices of mammals. In contrast to the prominent expansion of UL in primates, we found that rodents exhibit a characteristic bias toward thicker DL (Fig. 1A). Rats, in particular, exhibit an exceptionally high abundance of DL subtypes, even when scrutinized against their close evolutionary relatives, mice. Histological assessments revealed clear species-specific variations in cortical excitatory neuron composition: rats exhibit significantly more DL neurons per cortical column than mice, while UL neuron numbers remain comparable between the two species (Fig. 1). Clonal analysis demonstrated that individual rat RG progenitors produce a greater number of DL neurons than those in mice, suggesting that distinct progenitor activity drives these species-specific differences (Fig. 2). Birthdating assays further indicated an extended phase of DL neurogenesis in rats, followed by a conserved UL generation phase (Figs. 3 and  4). Our scRNAseq analysis revealed that rats maintain the early progenitor state associated with DL neuron production for a longer period than mice, as evidenced by the sustained expression of early-biased progenitor age genes (Fig. 6). More detailed analysis revealed that rat RG progenitors exhibit significantly elevated and sustained expression of Wnt-related genes, most notably those encoding Wnt ligands, compared to the counterparts in mice. The integrated analysis with transcriptomic data from other mammalian species demonstrated that this molecular signature in rats is exceptional (Fig. 7). These findings demonstrate that rats extend the DL neurogenetic phase by prolonging the Wnt-high period, reflecting a species-specific, non-uniform scaling of an evolutionarily conserved neurogenic program (Fig. 7K).

### Evolutionary adaptations in DL neuron composition

Our study demonstrates that mice and rats have undergone distinct evolutionary adaptations in the abundance of DL neurons. This pattern contrasts with the human lineage, where cortical thickening is characterized primarily by an increase in UL neurons (Fig. 1A). In rats, all DL subtypes examined—including Tbr1-, Ctip2-, and Satb2-expressing neurons—were more abundant than in mice, whereas UL neuron numbers remained largely conserved (Fig. 1). This DL-biased thickening in rats was evident in the primary somatosensory area, but is consistently observed throughout the rostral-caudal cortical regions. This finding is consistent with anatomical observations: the rat corticospinal tract, derived from DL neurons, is considerably larger than its counterpart in mice, despite only minor differences in the thickness of the corpus callosum, which originates from UL neurons (Appendix Fig. S4). We speculate that an increase in Ctip2-expressing CSPNs in layer 5 may be linked to the larger body size of rats and their greater demands for motor control. Furthermore, although UL neuron abundance was comparable between species, rats exhibited a higher number of Satb2-positive neurons within the DL. This suggests an expansion of descending outputs from cortico-cortical circuits. Notably, Satb2-positive DL neurons have dual projections to both callosal and brainstem targets, in contrast to UL neurons, which project exclusively to callosal pathways (Sinopoulou et al, 2022; Harb et al, 2016). Finally, we note that the cortical surface is substantially more expanded in rats compared with mice. This expansion likely reflects a greater degree of progenitor amplification prior to the onset of neurogenesis. While the overall DL-to-UL ratio remains unchanged, this early amplification increases the total number of cortical neurons that contribute to axonal projections, thereby enhancing the output capacity of rat cortical circuits.

We posit that further studies on species-specific DL neuron subtypes could clarify how anatomical differences influence functional and cognitive capabilities. The diversity of DL projection targets is also of interest. Our axon labeling experiments primarily classified projections as intracortical or extracortical (Fig. EV2). More detailed mapping of DL subtype projection patterns in rats (Sinopoulou et al, 2022), as has been done in mice (Gao et al, 2022; Muñoz-Castañeda et al, 2021), could elucidate interspecies differences in the extracortical projectome. The potential behavioral consequences of varying DL neuron abundance and complexity remain to be explored. While comparative behavioral studies in mice and rats are important, a more precise understanding could be gained from genetically modified mice with specific alterations in DL abundance (Zhang et al, 2020). This raises the broader question of whether cortical neuronal composition reflects evolutionary adaptations to distinct ecological niches. Rodent species occupy diverse habitats, and their brain architectures likely facilitate survival in these environments (Verde Arregoitia et al, 2017; Verde Arregoitia and D’Elía, 2021). Investigating how specific cortical features enhance evolutionary fitness would provide valuable insights.

### Temporal regulation of cortical neurogenesis by the canonical Wnt signaling pathway

Canonical Wnt signaling serves as a key temporal regulator of cortical neurogenesis (Munji et al, 2011; Vitali et al, 2018; Oberst et al, 2019). In both mice and humans, pathway activity is intrinsically high during early neurogenesis and diminishes as development proceeds (Nowakowski et al, 2017; Telley et al, 2019). Experimental perturbations in mouse RG progenitors demonstrate causality: elevating Wnt signaling favors the production of DL neurons, whereas reducing it promotes UL identities (Munji et al, 2011; Vitali et al, 2018; Wrobel et al, 2007). Heterochronic transplantation further supports this Wnt-dependent temporal control (Oberst et al, 2019). Remarkably, late-stage RG placed into an early Wnt-rich VZ environment reacquires DL potential, and this “rejuvenation” requires intact Wnt signaling.

Our data, together with prior work (Oberst et al, 2019), indicate that the Wnt signaling level in RG is governed primarily by the developmental dynamics of Wnt ligands rather than by their receptors (Figs. 7 and EV4C; Appendix Fig. S13A). Across stages, Wnt receptor expression is comparatively stable, preserving the intrinsic ability of RG to sense Wnt inputs, whereas ligand expression changes markedly. Consequently, when late-stage RGs encounter an early environment with abundant Wnt ligands, their constant receptor repertoire permits robust pathway activation and restoration of early-born DL output (Oberst et al, 2019). Based on these findings, the existing model (Oberst et al, 2019) posits that the concentration of Wnt ligands within the ventricular progenitor niche—initially high and subsequently decreasing—establishes the temporal window for RG neurogenic competence. Crucially, building upon this established framework, our comparative analysis reveals that species-specific differences in the temporal availability of Wnt ligands are a key determinant of interspecies variation in neuronal subtype balance.

Among the five mammals studied, the rat stands out as a clear exception. Despite species-specific expression changes in some Wnt-related genes, such as the human-specific upregulation of *Fzd8* (Boyd et al, 2015; Liu et al, 2025), rat RGs exhibit an unusually prolonged, high expression of a significant number of Wnt ligand genes and the Wnt readout gene *Axin2* (Fig. 7). Our perturbation experiment revealed that this sustained ligand milieu likely contributes to the extended production of DL neurons in rats. It is tempting to speculate what sustains high Wnt levels specifically in rats. Known sources of cortical Wnt ligands include the cortical hem and postmitotic neurons (Caronia-Brown et al, 2014; Grove et al, 1998; Subramanian and Tole, 2009; Ozair et al, 2018; Qian et al, 2020). The hem, the dorsomedial telencephalic organizer, expresses multiple Wnt ligands and is under the control of the master regulator *Lmx1a*: inhibiting *Lmx1a* suppresses the expression of several Wnt ligands, while forced *Lmx1a* expression in the future hippocampal region can induce *Wnt3a (*Iskusnykh et al, 2022*)*. In the midbrain, *Lmx1a* and *Wnt1* engage in a positive regulatory loop (Chung et al, 2009). Consistent with a strong medial Wnt output, the pathway readout *Axin2* is highest medially and tapers laterally (Bonnefont et al, 2019; Sun et al, 2023). In parallel, early-born subplate and DL neurons express ligands such as *Wnt7b*, which can bias newly generated neurons toward DL fates (Qian et al, 2020; Ozair et al, 2018).

Extending these observations, our scRNAseq analysis shows that rat RG themselves express multiple Wnt ligands together with the essential secretion factor *Wls* (Fig. 7). In rats, although the absolute level of *Lmx1a* expression in cortical RGs is even lower than in the cortical hem, as revealed by RNAscope-based in situ hybridization, a co-expression module that remains elevated comprises Wnt ligands, *Wls*, and *Lmx1a*, recapitulating a hem-like regulatory architecture in RG progenitors. This implicates *Lmx1a* as a potential upstream driver of Wnt ligand expression within RG. Notably, only in rats did a substantial subset of cortical RG co-express *Lmx1a* and *Lhx2* (Fig. EV3), even though in mice the cortical fate selector *Lhx2* suppresses *Lmx1a*, a master regulator of the cortical hem (Mangale et al, 2008). In our comparative transcriptome, the other mammals examined showed virtually no RGs with this co-expression, suggesting a rat-specific co-option of an *Lmx1a*–Wnt ligand cascade in cortical RG, potentially via relief of *Lhx2*-mediated repression of *Lmx1a*. We anticipate that dissecting the regulatory logic that induces *Lmx1a* in rat cortical RG, and testing the requirement of *Lmx1a* for extending the DL production window, will be informative. Together, these findings support a model in which a sustained, RG-intrinsic and niche-derived Wnt ligand environment, potentially orchestrated by *Lmx1a*, prolongs DL neurogenesis in the rat and helps explain species-specific differences in cortical neuron subtype composition.

### Evolvability of the mammalian cortical neurogenesis

Our study highlights a distinction between conserved and evolved components of the mammalian neurogenetic program. The early-to-late pattern of cortical neurogenesis, regulated chiefly by canonical Wnt signaling, is highly conserved across mammals (Jabaudon, 2017; Vanderhaeghen and Polleux, 2023; Rakic, 2009; Lui et al, 2011; Taverna et al, 2014) and beyond (Suzuki et al, 2012; Suzuki and Hirata, 2013; Furlan et al, 2017), suggesting that this developmental process has been maintained due to robust constraints (Carroll, 2008; Hall, 2012). Despite its apparent rigidity, our comparative analysis of closely related species revealed that subtle adjustments of heterochronic regulation can drive species-specific cortical neuron compositions without fundamentally altering the overall developmental framework. Specifically, the genetic system activating Wnt ligand expression, presumably driven by *Lmx1a*, was co-opted in early RG progenitors during the evolutionary specification of rats. Consequently, the temporal dynamics of Wnt signaling activity was fine-tuned to prolong the DL subtype-producing period. This non-uniform scaling of temporal progression mirrors differences observed in other developmental processes, such as cell differentiation trajectories during gastrulation in mice and rabbits (Mayshar et al, 2023) and brain development in eutherians and marsupials (Paolino et al, 2023). However, it contrasts with the relatively uniform elongation of developmental processes observed in humans compared to mice, as evidenced by studies on cortical neuron maturation (Iwata et al, 2023; Ciceri et al, 2024; Charrier et al, 2012), spinal motoneuron generation (Rayon et al, 2020), and somitogenesis (Matsuda et al, 2020; Lázaro et al, 2023; Diaz-Cuadros et al, 2023). Future comparative studies across a broader range of species could help clarify the balance between highly constrained components of the neurogenetic program and its more adaptable elements. Such insights would illuminate how evolutionary diversification optimized cortical architectures for species-specific functional and ecological needs.

### Limitations of the study

This study focuses on the generation of cortical excitatory neuron subtypes in mice and rats, leaving several critical areas unexplored. For instance, we did not examine progenitor amplification preceding neurogenesis in detail, despite notable differences in cortical neuroepithelial sheet size at the onset of neurogenesis. Investigating how neuroepithelial expansion varies between species is crucial for understanding the determinants of species-specific cortical volume and surface area. In addition, our study did not address other cell types, such as inhibitory neurons and glia, in depth. For example, rats exhibit a higher number of proliferating Olig2-positive glial progenitors during early postnatal periods compared to mice (Fig. EV1E). These differences in postnatal cell division events may align with the large deviations in developmental tempo observed in progenitors during the late phase of neurogenesis, as revealed by scRNAseq (Fig. 6F). Such findings suggest that rats may possess an increased abundance of glial progenitors, potentially resulting in greater diversity and quantity of glial cells.

## Methods

**Table:** Reagents and tools table

Reagent/resource	Reference or source	Identifier or catalog number
**Experimental models**		
Mouse: ICR (CD1)	Japan SLC, Inc.	N/A
Rat: Wistar ST	Japan SLC, Inc.	N/A
**Recombinant DNA**		
pLPGK-H2B-mCherry	In this study	N/A
pLC-mCherry	In this study	N/A
pCAG-EGFP	In this study	N/A
pCMV-VSV-G	Addgene	8454
pUMVC	Addgene	8449
pRC-2A-EGFP	Kind gift from Dr. Pierre Vanderhaeghen and Ryohei Iwata	N/C
pRC-mCherry	In this study	N/C
pCAG-IRES-GFP	GenScript Inc.	N/C
pLPGK-H2B-mCherry	In this study	N/C
pHes7-AchillesHes7	Addgene	153528
pLC-dnTCF7L2-flag-ires-EGFP	In this study	N/C
**Antibodies**		
Chicken anti-GFP	Abeam	ab13970
Rabbit anti-RFP	Rockland	600-401-379
Rat anti-Vglut2	Synaptic Systems	135403
Rat anti-Ctip2	Abcam	ab18465
Mouse anti-Satb2	Abcam	ab51502
Rabbit anti-Satb2	Abcam	ab34735
Goat anti-Olig2	R&D Systems	AF2418-SP
Rabbit anti-Tbr1	Abcam	ab183032
Rabbit anti-Pax6	MBL	PD022
Mouse anti-β3tublin	Covance	MMS-435P
Mouse anti-NeuN	Sigma-Aldrich	MAB377
Rat anti-PH3	Abcam	ab10543
Rabbit anti-Tbr2	Atlas Antibodies	HPA028896
Rat anti-Tbr2	Thermo Fisher	14-4875-82
Rabbit anti-Cux1	Proteintech	11733-1-AP
Mouse anti- RORbeta	Perseus proteomics	PP-N7927-00
Rabbit anti-Wnt3a	Abcam	ab219412
Rabbit anti-Lhx2	Abcam	ab184337
Chicken IgY (H + L)-Alexa fluor 488 conjugated	Jackson immunoresearch	703-545-155
Mouse IgG (H + L)-Alexa fluor 405 conjugated	Abcam	ab175659
Mouse IgG (H + L)-Alexa fluor 555 conjugated	Invitrogen	A31570
Rat IgG (H + L) -Cy3 conjugated	Jackson immunoresearch	712-165-153
Rat IgG (H + L)-Alexa fluor 647 conjugated	Invitrogen	A48272
Goat IgG (H + L)-Alexa fluor 647 conjugated	Invitrogen	A21447
Rabbit IgG (H + L)-Alexa fluor 488 conjugated	Invitrogen	A21206
Rabbit IgG (H + L)-Alexa fluor 555 conjugated	Invitrogen	A31572
Rabbit IgG (H + L)-Alexa fluor 647 conjugated	Invitrogen	A31573
**Oligonucleotides and other sequence-based reagents**		
RNAscope™ Probe- Rn-Axin2-C2	ADC	463881-C2
RNAscopeTM Target Probe - Mm-Lmx1a	ADC	493131
RNAscopeTM Probe - Rn-Lmx1a-O1-C1	ADC	1860131-C1
**Chemicals, enzymes, and other reagents**		
Opti-MEM	gibco	31985-062
D-PBS	Nakarai	14249-95
HBSS	Sigma	H6648-500ML
Sucrose	Fujifilm	196-00015
glycerol mounting medium	DAKO	C0563
Normal donkey serum	Jackson ImmunoResearch	017-000-121
Bovine Serum Albumin FractionⅤ	Merck	10735086001
BSA Fraction V 7.5%	Gibco	15260-037
5-ethynyl-2’-deoxyuridine	Fujifilm	052-08843
Papain	Sigma	76216-50MG
DNase	Sigma	D4513
EDTA	Sigma	D4513-1VL
isoflurane	Viatris Inc.	114133403
Click-iT™ Plus EdU Cell Proliferation Kit for Imaging, Alexa Fluor™ 488 dye	Thermo Fisher Scientific	C10637
X-tremeGENE	Merck	6366236001
CUBIC trial kit	Tokyo Chemical Industry Co.	C3942
RNAscopeTM Multiplex Fluorescent Detection Kit V2 (Kit Components)	ADC	323110
RNAscope TSA Buffer Pack 3BOTTLE	ADC	322810
TSA Vivid Fluorophore 520	ACD	323271
TSA Vivid Fluorophore 570	ACD	323272
TSA Vivid Fluorophore 650	ACD	323273
Paraformaldehyde (powder, 500 g)	Nakarai	26126-25
Agarose S (1000 g)	NIPPON GENE	313-90231
LB-Medium Capsule	MP Biomedicals	3002021
QIAfilter Plasmid Maxi Kit (25)	Qiagen	12263
NEB Stable Competent E. coli (High Efficiency)	NEB	Cat# C3040H
**Software**		
Fiji (ImageJ)	Fiji (ImageJ); National Institutes of Health	Version 1.0
GraphPad Prism	GraphPad Prism; GraphPad Software	Version 10.2.3
Microsoft Excel	Microsoft Excel; Microsoft Corporation	Version 16.82
**Other**		
Scaning fluorescent microscope	Keyence	BZ-X800
Confocal microscope	Olympus	FV3000
Electroporator NEPA21 TypeⅡ	Nepa Gene	N/A
Tweezer electrodes	Nepa Gene	CUY650P5
Vibratome	Leica	VT1200S
0.45 µm filter	sartorius	17598k
Grass capillary	Harvard apparatus	300066
µ-Dish 35 mm, high Glass Bottom	Ibidi	81158
Coverslips (24 × 50 mm, NEO, 200 sheets × 5 boxes)	Matsunami Glass Ind., Ltd.	C024501
MAS-coated glass slides (Glass Pink)	Matsunami Glass Ind., Ltd.	MAS-03
Glass slides, Fine Frost (White)	Matsunami Glass Ind., Ltd.	FF-001
Superfrost glass slides (White, S2441)	Matsunami Glass Ind.	S2441
OCT compound	Sakura Finetek Japan Co., Ltd.	45833
Peristaltic pump	ATTA	AC-2110Ⅱ
Nylon round brush (tip width: 4 mm)	MONOTORO	08471803
Nylon round brush (tip width: 6 mm)	MONOTORO	08471812
Tissue culture dish 10 cm	Falcon	353003
Centrifuge tubes (15 mL, bulk pack, 400 tubes)	VioLamo (AS ONE),	4-3632-01
Centrifuge tubes (50 mL, bulk pack, 200 tubes)	VioLamo (AS ONE),	4-3632-02

### Methods and protocols

#### Animals

All animal procedures were approved by the Institutional Safety Committee on Recombinant DNA Experiments and the Animal Research Committee of the University of Tokyo. ICR mice and Wistar ST rats were purchased from SLC Japan. Mice and rats were housed in cages with bedding (Avidity Science, TEK-FRESH) and provided constant access to food (Nippon Crea, Rodent Diet CE-2) and water. The animal facility was maintained at a temperature of 23 ± 2 °C, a humidity of 50 ± 10%, and a 12-h light/dark cycle. Plug day was defined as embryonic day (E)0.5, and the day of birth as postnatal day (P)0. Data from all embryos were pooled without discrimination of sex, given the difficulty of determining sex identity at embryonic stages. Rabbit and the large Japanese field mouse were obtained in the previous project in Tokyo Metropolitan Institute of Medical Science (Tsurugizawa et al, 2025). Ferrets were provided by SLC Japan and Marshall Farms (North Rose, NY), and Hartley guinea pigs were obtained from SLC Japan in the previous project in Kumamoto University (Hatakeyama et al, 2017).

#### Nissl staining and neuronal cell number count

We performed Nissl staining following the protocol provided with the kit (Thermofisher, ＃N21483). Briefly, cryosections were rehydrated for ≥40 min in 0.1 M PBS (pH 7.2), permeabilized for 10 min in PBS with 0.1% Triton X-100, and rinsed. NeuroTrace was diluted in PBS (100×), applied to cover the tissue (800 µL/slide) for 20 min, removed, and sections were washed in PBS/0.1% Triton X-100 (10 min), PBS (2 × 5 min), then PBS for 2 h at room temperature or overnight at 4 °C. Sections were mounted with DAKO glycerol mounting medium (Cat# C0563) and stored in the dark at 4 °C. Human and macaque (primary somatosensory cortex, face- and neck-related subregions) as well as adult mouse (somatosensory barrel field) brain sections were obtained from the Allen Brain Reference Atlas, with detailed image identifiers listed in the corresponding supplementary figures. Mouse, rat, ferret, the large Japanese field mouse, guinea pig, and rabbit brain sections (adult somatosensory barrel field, *n* = 1 per species) were prepared in-house.

Cortical layers were delineated based on established cytoarchitectonic criteria (García-Cabezas et al, 2020; Guillery, 2000). Specifically, Layer I (molecular) is sparsely cellular; Layer II (external granular) contains small, densely packed granular neurons; Layer III (external pyramidal) features medium-sized pyramidal neurons; Layer IV (internal granular) is characterized by small, densely packed granular neurons; Layer V (internal pyramidal) includes large pyramidal neurons; and Layer VI (multiform) comprises a mix of neuronal morphologies with lower density near the white matter boundary.

Neurons were identified by their prominent Nissl-positive soma containing basophilic granular material, distinguishing them from glia (smaller, with scant cytoplasm) and other non-neuronal cells. Using Fiji/ImageJ software (Schindelin et al, 2015), regions of interest spanning the full cortical depth were outlined, and neurons were manually counted within standardized sampling frames (100 µm × 100 µm) at multiple sites per layer to ensure representative coverage.

#### Preparation of retroviruses

HEK293T cells were seeded to reach 80–90% confluency on a 10 cm dish. After 24 h, the cells were transfected with a mixture containing: 0.85 µg pCMV-VSV-G (Addgene #8454), 9.90 µg pUMVC (Addgene #8449), 8.25 µg pRC-2A-EGFP (kind gift from Dr. Pierre Vanderhaeghen and Ryohei Iwata) or pRC-mCherry (prepared in this study). The transfection was performed in 1200 µL of Opti-MEM supplemented with 60 µL of X-tremeGENE. After 24 h, the medium was replaced, and the cells were cultured for another 24 h. The supernatant was then filtered through a 0.45-µm filter, and retroviral particles were concentrated by centrifugation at 25,000 rpm (82,700 g) at 4 °C for 2 h using a 20% sucrose/PBS cushion. The viral pellet was resuspended in 200 µL of cold DPBS, aliquoted in 20 µL, and stored at −80 °C. Viral titers were checked using HEK293T cells for each batch.

#### Retroviral injection to animals in utero for clonal tracing

Pregnant females were anesthetized with isoflurane (Viatris, Cat# 114133403), and the uterine horns were exposed under sterile conditions. One microliter of retroviral solution mixed with 1% Fast Green was injected into the fetal lateral ventricles using a heat-pulled capillary. After injection, the uterine horns were returned to the abdominal cavity, and the incisions were sutured. Mice were kept on a heating plate until fully recovered.

#### In utero electroporation

In utero electroporation was performed with modifications from established protocols (Tabata and Nakajima, 2001; Suzuki et al, 2018). Timed-pregnant rats (E16.5) or mice were anesthetized with isoflurane. Plasmid solutions (1–1.5 mg/mL DNA) were injected into the embryonic lateral ventricles using heat-pulled capillaries. Electroporation was conducted using tweezer electrodes (Nepa Gene, Cat# CUY650P5) connected to a NEPA21 Type II electroporator (Nepa Gene) with the following settings: Rats: Voltage 50 V, pulse duration 50 ms, interval 950 ms, 4 pulses. Mice: Voltage 50 V, pulse duration 50 ms, interval 950 ms, 6 pulses. Embryos were returned to the abdominal cavity, and the mothers were sutured and placed on a heating plate until recovery. Brains were collected from electroporated pups at P7. Coronal sections (100 μm thickness) were prepared, and sections corresponding to the level where the hippocampus first becomes visible were selected. Images were acquired from the primary somatosensory cortex (S1) within these sections.

#### DNA constructs

Membrane-anchored Achilles was amplified by PCR using primers with a membrane targeting signal sequence from the template plasmid (Addgene #153528) and subcloned into a pCAG plasmid.

#### Histology and immunostaining

Immunostaining was performed as described previously (Suzuki et al, 2012, 2018). Mouse embryos were collected after electroporation and perfused transcardially with ice-cold 4% paraformaldehyde (PFA) in PBS. Dissected brains were soaked in 4% PFA overnight at 4 °C and sectioned into 100 µm slices using a vibratome (Leica, Cat# VT1000S). Slices were washed with PBST (PBS with 0.1% Triton X-100) three times and incubated in blocking solution (PBS with 0.3% Triton X-100 and 3% donkey serum) for 30 min. Brain slices were then incubated overnight at 4 °C with primary antibodies (Reagents and tools table). After three PBST washes, slices were incubated with secondary antibodies (Appendix Table S1) for 2 h at room temperature. After additional PBST washes, slices were mounted on slide glasses using DAKO glycerol mounting medium (Cat# C0563). Imaging was performed with a confocal microscope (Olympus FV3000), and images were processed using Fiji/ImageJ software (Schindelin et al, 2015).

#### Clonal analysis

For clonal analysis, cortical sections were immunostained with anti-GFP and anti-RFP antibodies and imaged sequentially from rostral to caudal using a Slide Scanning Microscope (Keyence, Cat# BZ-X800). Clones were identified as sparse, spatially isolated cell clusters, typically separated by at least 500 µm. Cortical layers (UL vs. DL) were delineated using DAPI staining to define laminar boundaries, and the positions of labeled cells were recorded. Clones were categorized into UL-only, DL-only, or Mixed (UL and DL) types based on the laminar positions of their constituent cells. Clonal lineages with fewer than three cells or more than 30 cells were excluded from quantitative analyses.

#### Neuronal birthdating

Birthdating analysis was performed as described previously (Hirata et al, 2021). Pregnant animals were injected intraperitoneally with a thymidine analog, 5-ethynyl-2’-deoxyuridine (EdU, Fujifilm; Cat# 052-08843), at 45 mg/kg body weight at designated time points. Brains were collected at P7, and 100 µm coronal sections were prepared using a Vibratome (Leica, Cat# VT1200S). Sections were immunostained with anti-NeuN and anti-Olig2 antibodies to visualize neurons and glial progenitors. EdU incorporation was detected following the Invitrogen EdU detection kit protocol (Click-iT EdU Cell Proliferation Kit, Cat# C10637). EdU-positive cells were counted in cortical sections of 3–4 pups imaged with a confocal microscope (Olympus FV3000).

#### Tissue clearing and axonal tracing

Tissue clearing was performed using the CUBIC trial kit (Tokyo Chemical Industry Co., Cat# C3942). Fixed samples were washed in PBS for 2 h (repeated three times) and serially incubated in: 50% CUBIC-L solution for 12 h at room temperature (RT) 100% CUBIC-L solution for 72 h at 37 °C on a shaker. Samples were washed again in PBS for 2 h (repeated three times), pre-treated in 50% CUBIC-R+ solution for 24 h at RT on a shaker, and incubated in 100% CUBIC-R+ solution for 24 h at RT on a shaker. Cleared brains were preserved in CUBIC-R + , and imaging was conducted with a confocal microscope (Olympus FV3000) in a 3.5-mm dish. Images were processed using Fiji/ImageJ software (Schindelin et al, 2015).

#### Mouse and rat progenitor cell cycle length

To measure the cell cycle lengths of mouse and rat progenitor cells and assess their longitudinal changes, progenitor cells were dissociated from rat E13.5 cortices and mouse E11.5 cortices and cultured in vitro. Embryonic brains were collected at the corresponding developmental stages and dissociated in ice-cold HBSS (Cat# H6648-500ML, Sigma) containing 0.1% BSA (Cat# 15260-037, Gibco). Tissues were digested in a 25 U/mL papain solution (Cat# 76216-50MG, Sigma) supplemented with 55 U/mL DNase (Cat# D4513, Sigma) for 30 min. Dissociated cells were then electroporated with cytosolic GFP for cell tracking, following the manufacturer’s protocol for the Lonza P3 Primary Cell Nucleofection Kit (Catalog #: V4SP-3096). Briefly, 1 × 10⁶ cells per condition were transferred to a 15 mL conical tube, centrifuged at 160×*g* for 5 min, and the supernatant was discarded. The cell pellet was resuspended in 90 µL of Lonza P3 Primary Cell Nucleofection Solution (4:1 ratio with Supplement 1, including 5 µM ROCK inhibitor) and electroporated with cytosolic GFP vector.

GFP-labeled cells were seeded in µ-Dish 35 mm Quad dishes (ibidi, Cat. No. 80416) for time-lapse cell tracking experiments. Cells were cultured in DMEM/F-12 supplemented with N2 and B27 (without Vitamin A) and placed in a CO₂ incubator under microscopy. A custom-built fluorescence microscope, housed within a cell culture incubator to maintain optimal temperature and humidity, was used for imaging. Images were acquired every 30 min and reconstructed into time-series data for cell tracking and cell cycle length analysis. Cell cycle length was defined as the duration between two consecutive cell divisions observed in the time-lapse data. The occurrence time of each cell cycle length was defined as the midpoint between such consecutive division events.

#### Single-cell transcriptomics

*Sample preparation and sequencing*: Rat embryonic brains were collected at E14.5, E16.5, and E18.5 and dissociated in ice-cold HBSS (Cat# H6648-500ML, Sigma) containing 0.1% BSA (Cat# 15260-037, Gibco). Tissues were digested in a 25 U/mL papain solution (Cat# 76216-50MG, Sigma) with 55 U/mL DNase (Cat# D4513, Sigma) and 0.5 mM EDTA for 20 min. After dissociation and concentration, cells were resuspended in HBSS with 0.04% BSA and processed immediately for single-cell GEM formation, cDNA amplification, and library construction using the Chromium Next GEM Single Cell 3’ Kit v3.1 (10x Genomics, PN-1000269) according to the manufacturer’s protocol. Libraries were quantified using an Agilent Bioanalyzer and sequenced on the NovaSeq6000 platform (Illumina) with an average read depth of 45,238 (E14.5), 42,669 (E16.5), and 44,504 (E18.5) reads per cell. Sequenced reads were aligned to the rat genome (mRatBN7.2) and processed with CellRanger v6.0.1. Noise reduction was performed using RECODE software (Imoto et al, 2022).

*scRNAseq analysis*: Downstream analysis was conducted using Seurat v5 (Satija et al, 2015). For quality control, cells were filtered using thresholds of nUMI >15,000, nGene >15,000, log10GenesPerUMI > 0.9, and mitoRatio <0.25 to retain high-quality cells. To integrate rat scRNA-seq data with previously published mouse datasets (Di Bella et al, 2021; Ruan et al, 2021), we normalized and extracted the 3000 most variable genes using SCTransform and aligned cells between species with canonical correlation analysis (Stuart et al, 2019). PCA dimensionality reduction was performed on integrated data, retaining the first 50 PCs. Cell clustering was conducted using the Louvain algorithm (Blondel et al, 2008) with a resolution of 1.4. Cluster identities were assigned based on the top 50 differentially expressed genes (ranked by −log10 adjusted *P* value, Wilcoxon Rank-Sum test with Bonferroni correction), ensuring >30% cluster expression and a > 0.5 log-fold change threshold, alongside canonical markers (Fig. 6C).

*Pseudotime age prediction and progenitor age genes*: Pseudotime analysis followed established methods (Telley et al, 2019; Macnair et al, 2022). L1-regularized ordinal regression was used to predict progenitor cell ages (neuroepithelial and radial glial cells). Analysis was restricted to the 3000 integration genes to minimize batch effect and sequencing depth variations in the integrated datasets of rat and mouse cells (Di Bella et al, 2021). Highly variable genes were identified using the *scran* R package (Grün et al, 2016). We performed the *L1* penalized ordinal regression following the manual from the *psupertime* R package (Macnair et al, 2022). After the cross-validation (*n_folds* = 10), the weight of the optimized model was used to rank the genes according to their ability to predict each progenitor cell age. Genes with non-zero coefficients in both mouse and rat models were classified as “progenitor age genes,” reflecting developmental temporal processes (Dataset EV2).

*Dynamic time warping-based alignment of pseudotime trajectories in mouse and rat*: Dynamic time warping (DTW) of pseudotime trajectories was performed using CellAlign (Alpert et al, 2018). Pseudotemporal trajectories for each species were equally spaced into 500 points. Pairwise Euclidean distances between ordered points were calculated using the shared progenitor temporal genes. A fixed-start/fixed-end constraint was applied, identifying the optimal alignment path to minimize overall distance. Gaussian weighting (winSz = 0.1) was applied to reduce noise.

*WGCNA analysis and module identification*: Weighted gene co-expression network analysis (WGCNA) (Langfelder and Horvath, 2008) was performed to identify co-expression modules associated with neurodevelopmental dynamics in radial glia progenitors. Highly variable genes (HVGs) were first identified within the ventricular radial glia (vRG) cell subset from mouse and rat scRNA-seq datasets using the FindVariableFeatures function (selection.method = “vst”, nfeatures = 2000) in Seurat (v5). The intersection of mouse and rat HVGs yielded 431 genes exhibiting consistent temporal dynamics in rodent progenitor cells. To focus on temporal patterns while mitigating interspecies magnitude differences, gene expression values were log-normalized from RNA assay and then z-scored across pseudotime trajectories (with start and end points aligned between species based on established developmental correspondence). WGCNA was implemented using the WGCNA R package (v1.73) with the following parameters: soft-thresholding power = 6 (selected based on scale-free topology fit), TOMType = “signed Nowick”, and minModuleSize = 30 via the blockwiseModules function. This analysis identified four modules based on co-expression patterns (designated as modules 1–4, Dataset EV2). Module eigengenes (defined as the first principal component of each module’s gene expression matrix) were visualized in the upper left of the corresponding plots. Gene Ontology (GO) enrichment analysis was conducted for genes in each module using clusterProfiler (v4.6.2), with the most significant GO term (lowest FDR-adjusted *P* value) displayed in the plot. Co-expression networks were visualized by selecting the top 300 connections ranked by module membership (kME) values.

*Gene expression dynamics across five mammalian species*: Building on scRNA-seq datasets from developing somatosensory cortex in rat and mouse (Di Bella et al, 2021), we incorporated three additional datasets from human (Wang et al, 2025), rhesus macaque (Micali et al, 2023), and ferret (Bilgic et al, 2023) to examine progenitor gene expression dynamics across species. Developmental stages were curated to span the equivalent period of mouse E12–E16 (rat E14–E18) using an established translational timing model (Workman et al, 2013), corresponding to gestational week (GW) 10–15 in human, embryonic day (E) 42–64 in macaque, and E25–E40 in ferret. Following subsetting to progenitor populations, pseudotime trajectories were inferred using an ordinal logistic regression model (Macnair et al, 2022). Gene expression was log-normalized from RNA assay and z-scored across the pseudotime axis to capture temporal dynamics. To assess absolute expression levels while accounting for technical variability, values were retrieved from Seurat’s SCT assay and regressed against covariates (“nCount_RNA”, “nFeature_RNA”, “percent.mt”, “CC.Difference”) using the glmGamPoi method.

#### Fluorescent in situ hybridization with RNAscope

Mouse and rat cortical tissues were cryosectioned at 20 µm thickness using a Cryostat (Cat# CM3050S, Leica) and mounted on slide glasses (Cat# MAS-05, Matsunami Glass Ind., Ltd) following the RNAscope protocol (UM323110, ACDBio). Sections were stored at −80 °C until use. Preprocessing, probe hybridization, signal detection, and quantification were performed using the RNAscope Multiplex Fluorescent Detection Kit V2 (Cat# 32310, ACDBio). Species-specific Axin2 and Lmx1a probes were used, each designed to target conserved orthologous sequences for optimal hybridization efficiency in mice and rats, respectively.

#### Quantification and statistical analysis

Statistical analyses were performed using GraphPad Prism 10 (GraphPad Software, Inc., USA) and Microsoft Excel (Microsoft Inc., USA). Experiments were repeated at least three times, and results are expressed as mean ± SEM. Student’s *t* test: Figs. 1C,E, 5B,C,E,F, 7J; Appendix Figs. S2B,C,  S8, and S14B–D. Welch’s *t* test: Figs. 2E,F, 3B, 4C–K, and 7H. Quantification of data (e.g., positive cell counts and signal dot numbers) was performed in a blinded manner. Samples were anonymized prior to analysis, and counting was conducted by independent researchers unaware of sample identity. Statistical significance thresholds and all quantitative results are summarized in Dataset EV1.

## Supplementary information

Appendix

Peer Review File

Dataset EV1

Dataset EV2

Expanded View Figures

## Data Availability

All source data, including microscopic images and quantitative data necessary to reproduce the results, have been deposited in the BioImage Archive and are available at https://www.ebi.ac.uk/biostudies/bioimages/studies/S-BIAD3170. Analysis codes used in this study are available at the following GitHub repository (https://github.com/XD-Sheu/Rodents-temporal-scaling_2026/). Single-cell RNA sequencing data of rat cortical cells are available in the NCBI Gene Expression Omnibus (GEO: GSE287210). All animals and unique/stable reagents generated in this study are available from the lead contact with a completed Materials Transfer Agreement. The source data of this paper are collected in the following database record: biostudies:S-SCDT-10_1038-S44318-026-00806-z.
