# Supplementary material for: Interspecific diversity in the neuronal composition of the mammalian cortex arises from heterochrony in neurogenesis
Source: EMBO J. 2026 May 22;45(15):5237–66. doi: 10.1038/s44318-026-00806-z (PMC13434014; doi:10.1038/s44318-026-00806-z)
Supplement: Supplementary file 1 [file 44318_2026_806_MOESM1_ESM.pdf]

Appendix for:

Interspecific diversity in the neuronal composition of the mammalian cortex arises from heterochrony in neurogenesis

Table of Contents

Appendix Figure S1: Comparative anatomy of somatosensory cortices of mammalian species, related to Figure 1 .... **2**

Appendix Figure S2: Projection neuron marker expression and quantification in mouse and rat primary somatosensory cortex at P7, related to Figure 1 .... **4**

Appendix Figure S3: Morphological and histological comparison of mouse and rat cerebral cortices, related to Figure 1 .... **5**

Appendix Figure S4: Intracortical and corticofugal projections as well as projection neuronal markers in mice and rats, related to Figure 1 .... **6**

Appendix Figure S5: Clonal analysis with different retroviral injection timings, related to Figure 3 ... **7**

Appendix Figure S6: Marker immunoreactivity of EdU-labeled neurons, related to Figure 4 .... **8**

Appendix Figure S7: Cell cycle length of mouse and rat cortical progenitors, related to Figure 5 .... **9**

Appendix Figure S8: Temporal changes in the number of Tbr2^{+} intermediate progenitor cells in mouse and rat developmental cortex, related to Figure 5 .... **10**

Appendix Figure S9: Single-cell RNA sequencing of mouse and rat cortical cells, related to Figure 6 .... **11**

Appendix Figure S10: Identification of progenitor age genes using the ordinal regression model, related to Figure 6 .... **12**

Appendix Figure S11: Gene expression distances in primate and rodent cortical progenitors, related to Figure 6 .... **13**

Appendix Figure S12: Significantly elevated expression of Axin2 in rat RG progenitors compared to other mammals, related to Figure 7 .... **14**

Appendix Figure S13: Wnt ligand and receptor genes among five mammalian species, related to Figure 7 .... **15**

Appendix Figure S14: Inhibition of canonical Wnt signaling by introduction of dnTCF to RG progenitors alters neuronal subtype specification at E20.5, related to Figure 7 .... **16**

A Human primary somatosensory cortex ***nissl staining***

Allen brain reference atlases PMID: 27418273

Adult human (Y34) primary somatosensory S1
(head and face region, S1HF, tissue index: 1476)

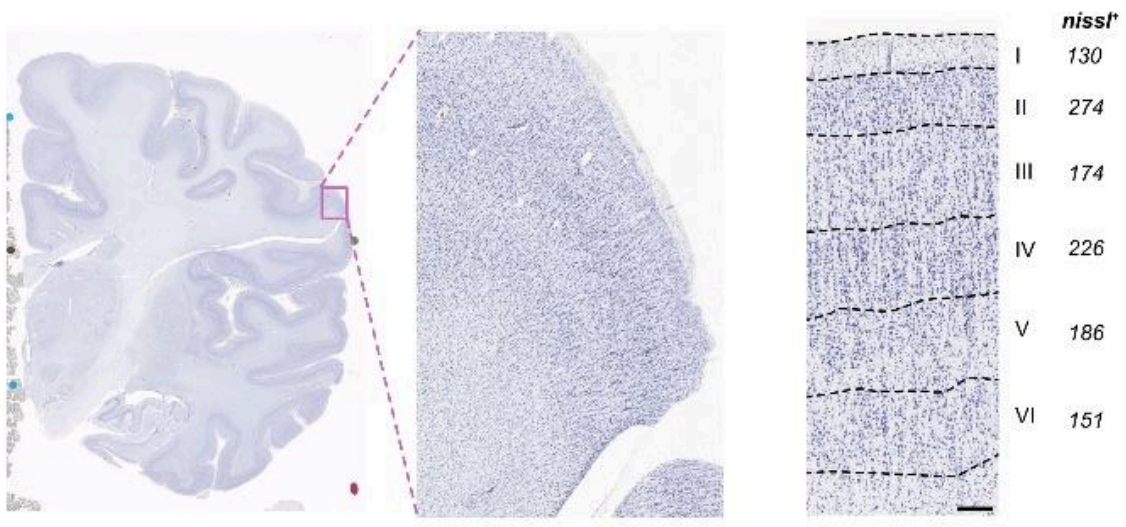

B Macaque primary somatosensory cortex ***nissl staining***

Allen brain reference, Macaca mulatta, NHP Atlas

Macaque (48 mo) primary somatosensory S1
(head and face region, S1HF, tissue index: 761)

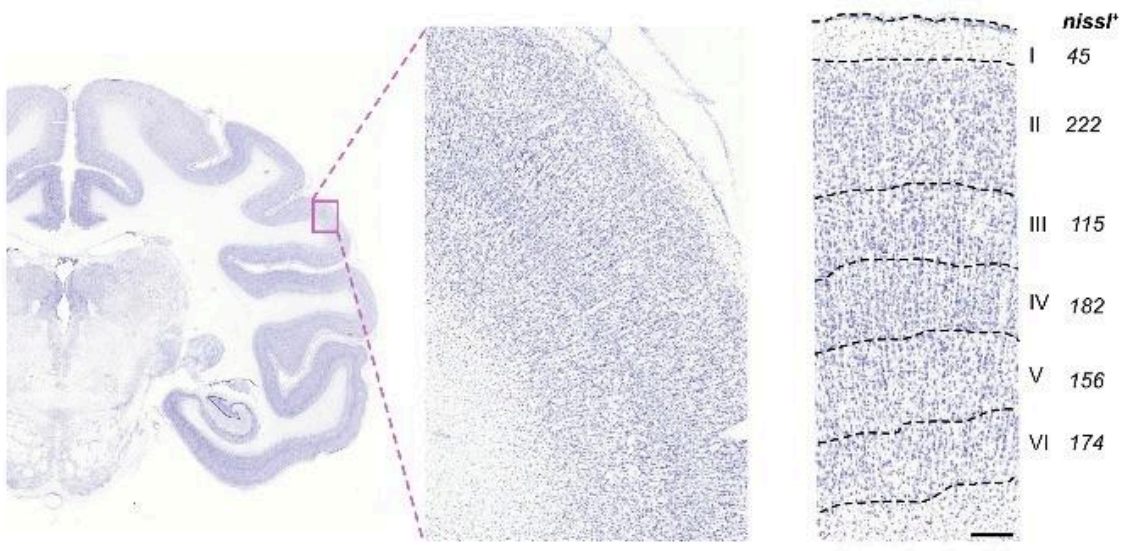

C Mouse primary somatosensory cortex ***nissl staining***

Allen brain reference atlases, Mouse, image 65 of 132, https://mouse.brain-map.org/experiment

Adult mouse (P56) somatosensory cortex S1 (Barrels field)

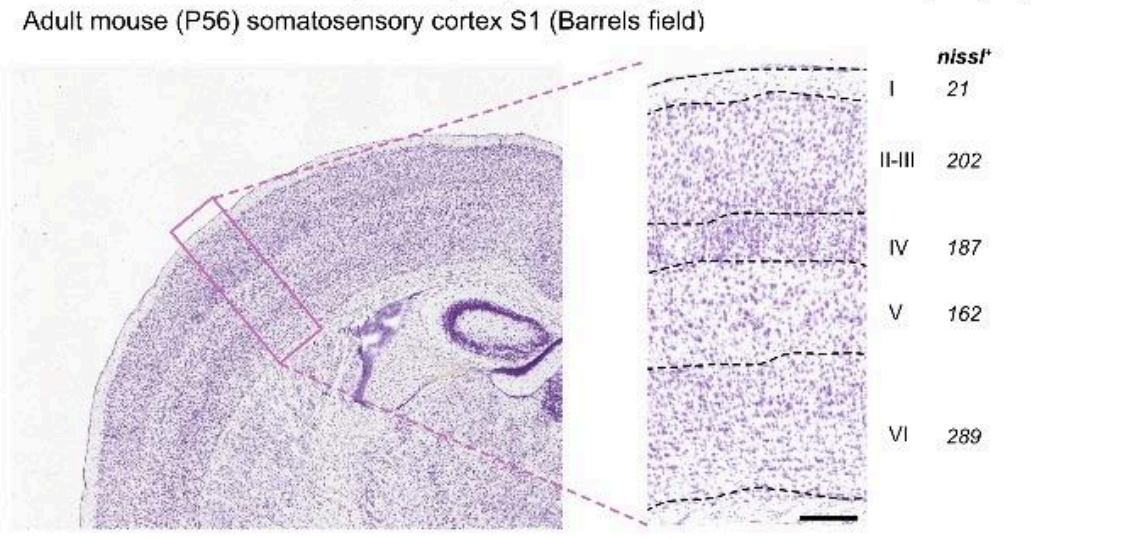

D Rat primary somatosensory cortex ***nissl staining***

Adult rat somatosensory cortex S1 (Barrels field)

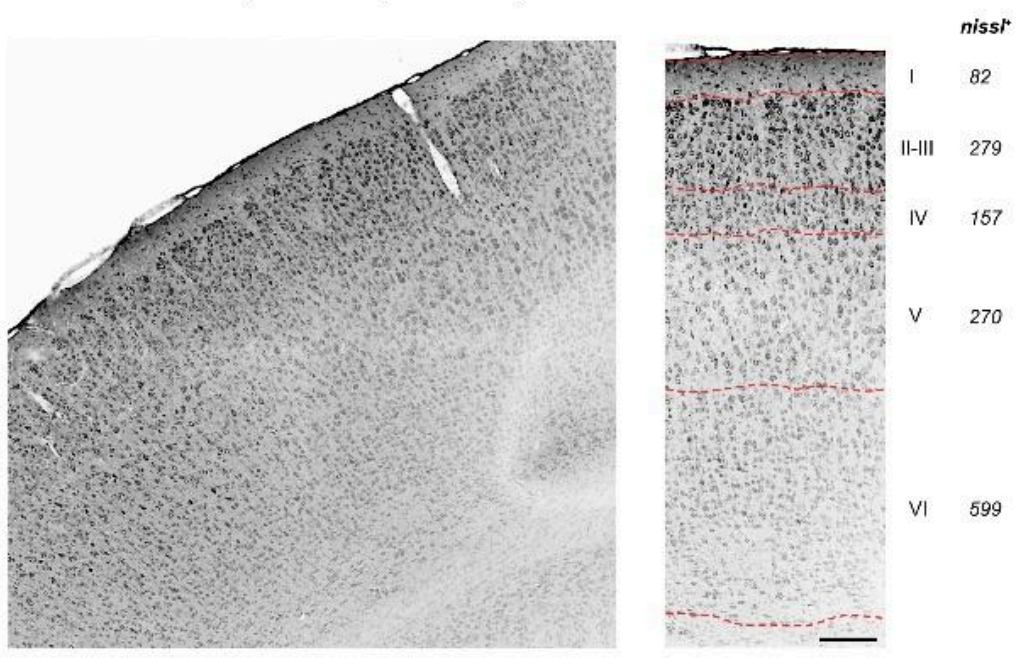

E Ferret primary somatosensory cortex ***nissl staining***

Brain sections were kindly provided by Dr. Jun Hatakeyama (Kumamoto University).

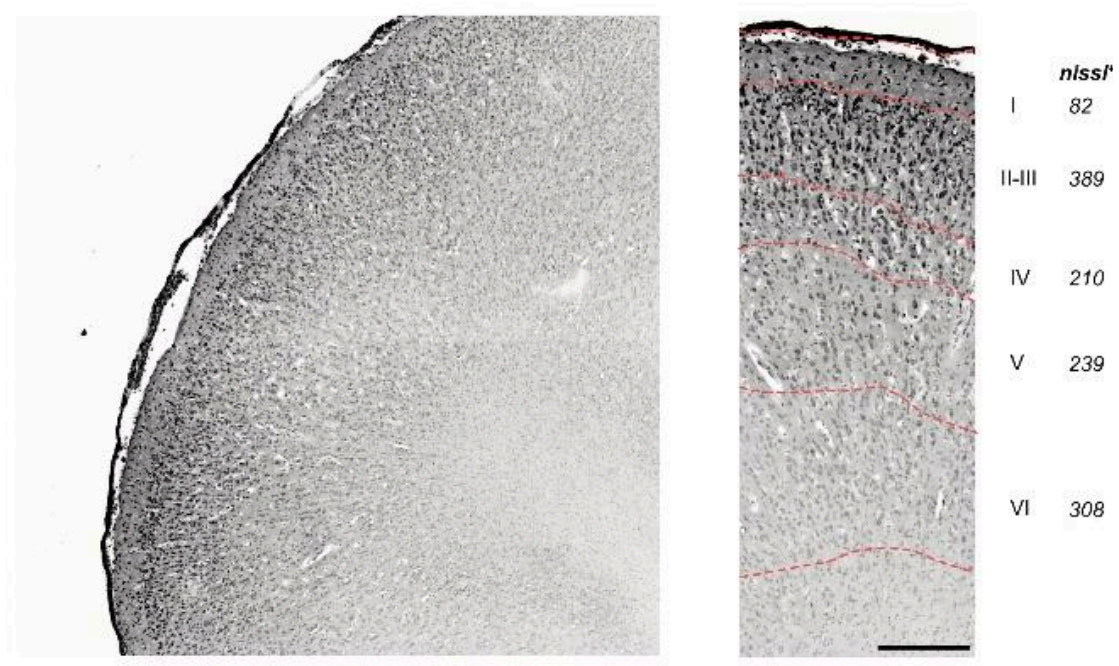

F Large Japanese field mouse primary somatosensory cortex

Brain sections were kindly provided by Dr. Takuma Kumamoto
(Tokyo Metropolitan Institute of Medical Science).

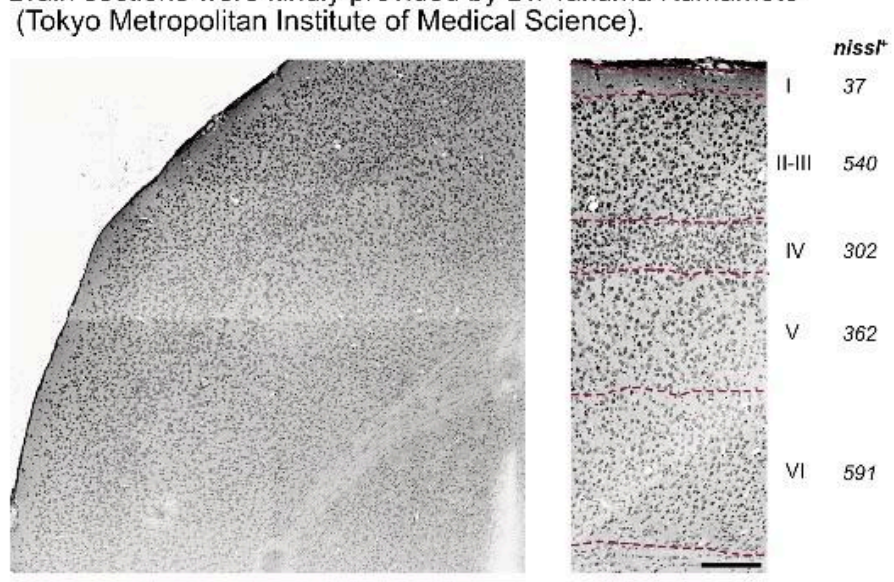

G Rabbit primary somatosensory cortex ***nissl staining***

Brain sections were kindly provided by Dr. Takuma Kumamoto
(Tokyo Metropolitan Institute of Medical Science).

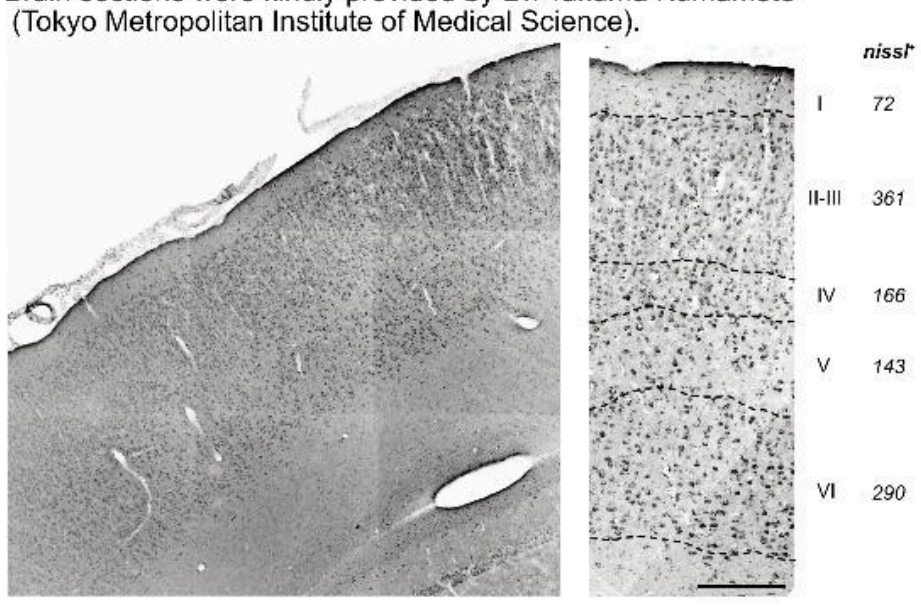

H Guinea pig primary somatosensory cortex ***nissl staining***

Brain sections were kindly provided by Dr. Jun Hatakeyama (Kumamoto University).

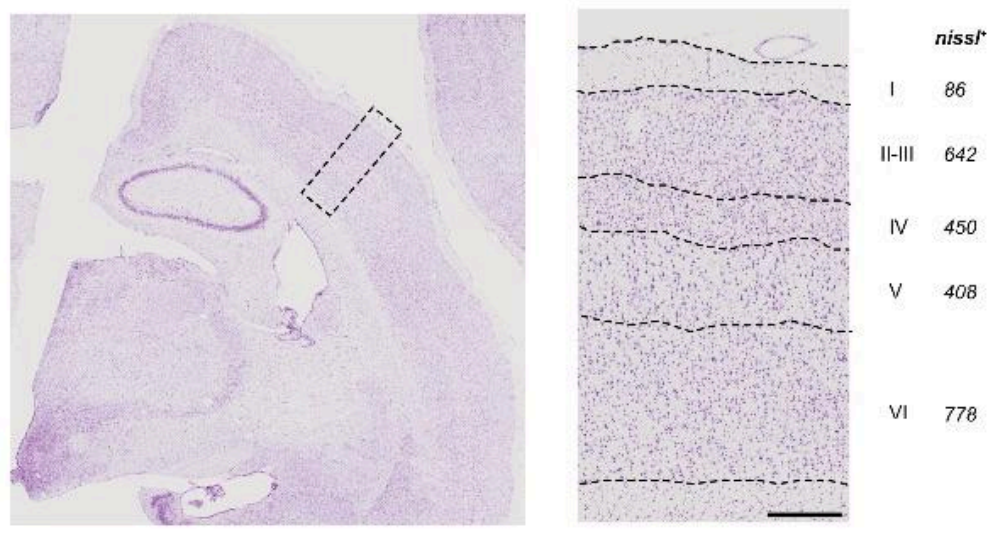

Appendix Figure S1. Comparative anatomy of somatosensory cortices of mammalian species, related to FIgure 1

A-H. Coronal brain sections from adult animals (human, macaque, mouse, rat, ferret, large Japanese field mouse, rabbit, and guinea pig) illustrating the somatosensory cortex are presented in panels **A to H**. In rodents (mouse, rat, large Japanese field mouse, and guinea pig), sections were selected to highlight the barrel fields within the primary somatosensory cortex. For non-rodent species (human, macaque, ferret, and rabbit), where barrel fields are less distinct, regions corresponding to head and neck sensory input in the primary somatosensory cortex were curated as the closest homologous areas. Nissl staining was performed on brain sections from rat, ferret, large Japanese field mouse, rabbit, and guinea pig (see Materials and Methods for details). Human, macaque, and mouse brain sections were sourced from the Allen Brain Institute reference atlas (https://mouse.brain-map.org/static/atlas) to ensure anatomical consistency in the selected regions.

Scale bars: 200µm **(A–H)**

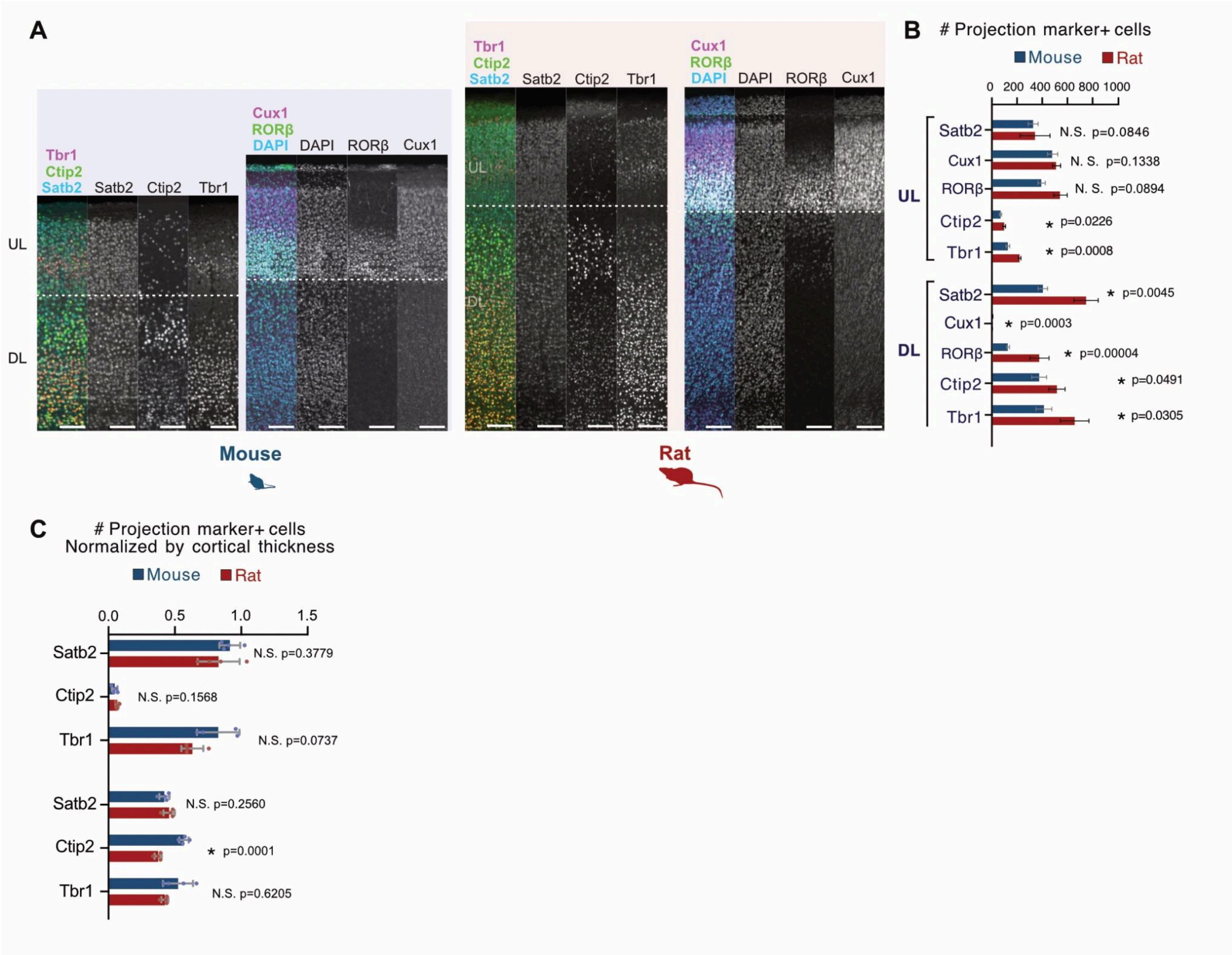

Appendix Figure S2. Projection neuron marker expression and quantification in mouse and rat primary somatosensory cortex at P7, related to Figure 1

A. Immunohistochemical staining for projection neuronal markers (Satb2, Cux1, RORβ, Ctip2 and Tbr1) in the primary somatosensory cortex (S1) of mice and rats at postnatal day 7 (P7). White dotted lines demarcate the boundary between upper layers (UL) and deeper layers (DL). **B.** Quantification of Satb2, Cux1, RORβ, Ctip2 and Tbr1 expressing neurons in the UL and DL of mouse and rat S1 at P7. Data are presented as mean ± SEM (n = 3 samples per group); statistical significance was determined using Student's t-test. **C.** Quantification of projection neuron marker–positive cells expressing Satb2, Ctip2, or Tbr1 in the developing mouse and rat cortex. Cell numbers were normalized to cortical thickness to account for species-specific differences in cortical size. Data are shown as mean ± SEM, with mouse indicated in blue and rat indicated in red. Scale bars: 100μm **(A).**

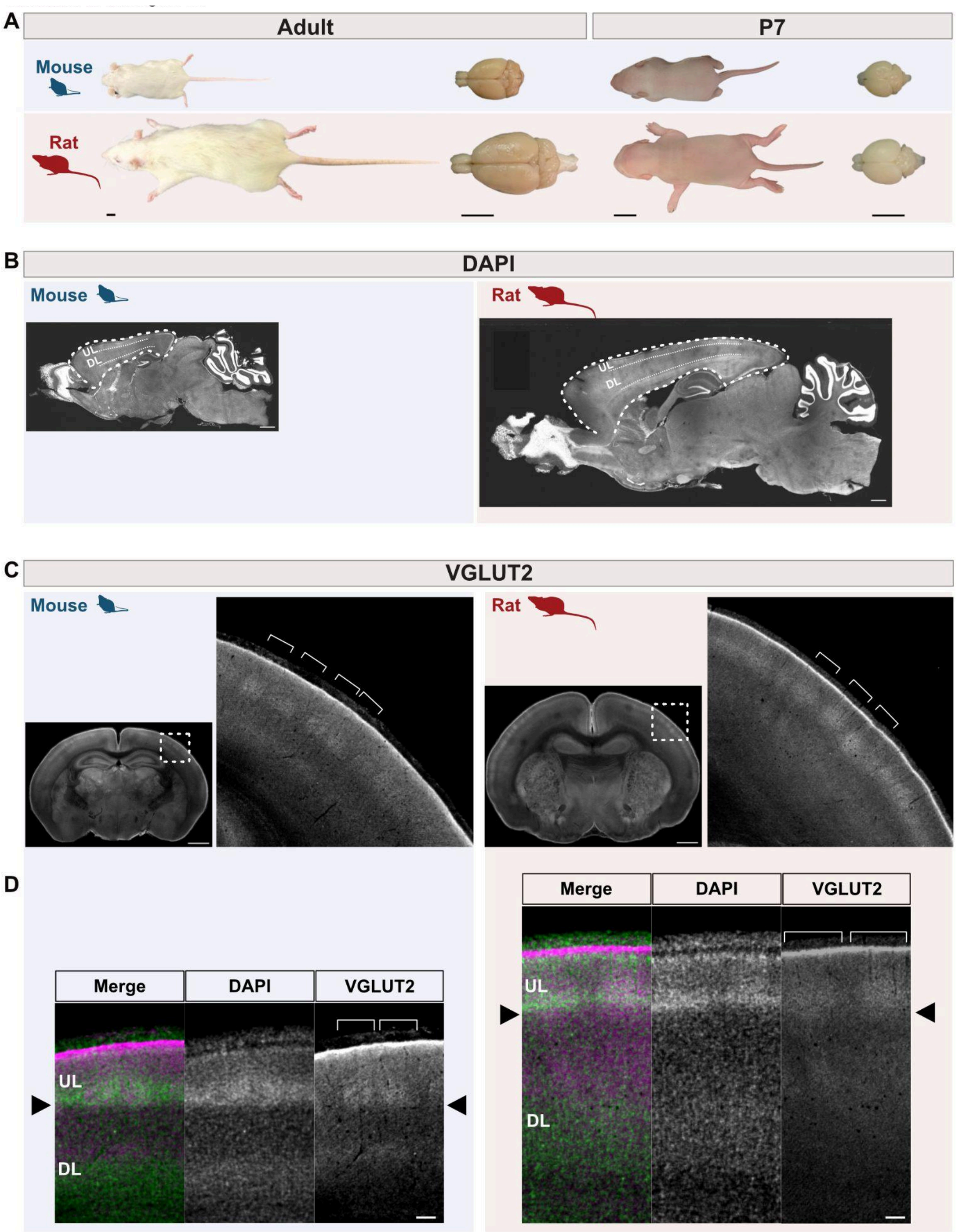

Appendix Figure S3. Morphological and histological comparison of mouse and rat cerebral cortices, related to Figure 1

A. Whole-body and brain size differences between mice and rats. **B**. Sagittal sections of mouse and rat brains at identical magnification. **C**. Analyses were conducted in the primary somatosensory field (S1) of both species, identified by the characteristic barrel structure of thalamic axon terminals visualized by VGLUT2 immunostaining. Magnified images highlight the structure within dotted rectangles. **D**. The border between UL (layer 2-4) and DL (layer 5 and 6) is defined by differences in cell density (DAPI staining) and the lower limit of VGLUT2-positive thalamic fibers (arrowheads). Scale bars: 1 cm (**A**), 1 mm (**B**), 1 mm (**C**), 100 µm (**D**).

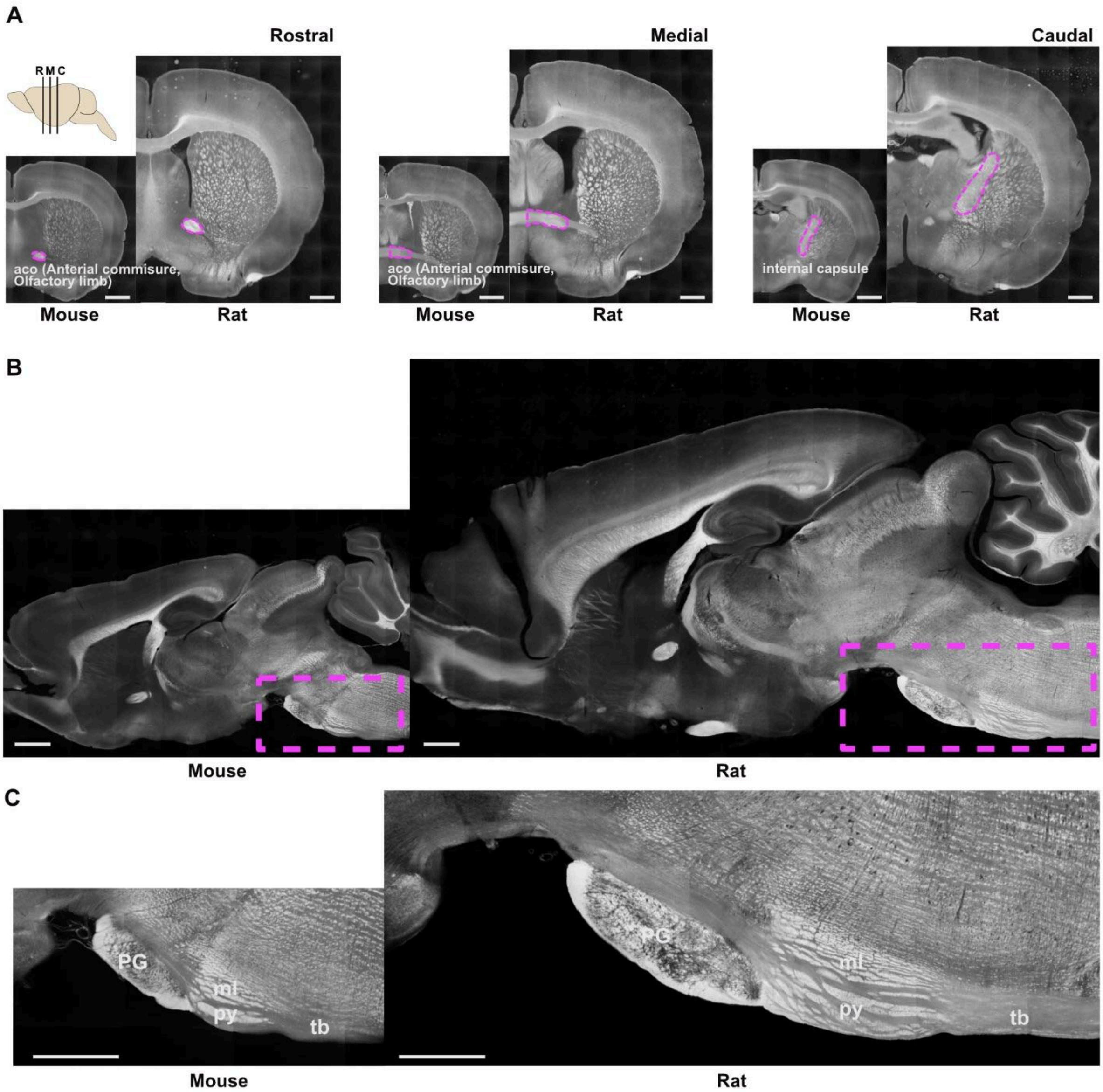

Appendix Figure S4. Intracortical and corticofugal projections as well as projection neuronal markers in mice and rats, related to Figure 1

A. Coronal sections of adult mouse and rat brains at rostral, middle, and caudal levels (R, M, C). Regions outlined with magenta dashed lines indicate the anterior commissure (ACO) and internal capsule (IC) in mice and rats, respectively. Axon-enriched areas appear whitish under transmitted light differential interference contrast (TD) imaging, clearly delineating the corpus callosum, ACO, and IC projections. **B.** Sagittal sections of adult mouse and rat brains visualizing corticospinal projections under TD imaging. Magenta dashed lines in panel **B** highlight corticospinal projections, with panel **C** providing an enlarged view of the corticospinal tract, including the pontine gray/nuclei (PG), medial lemniscus (ML), pyramidal tract (PY), and trapezoid body (TB).

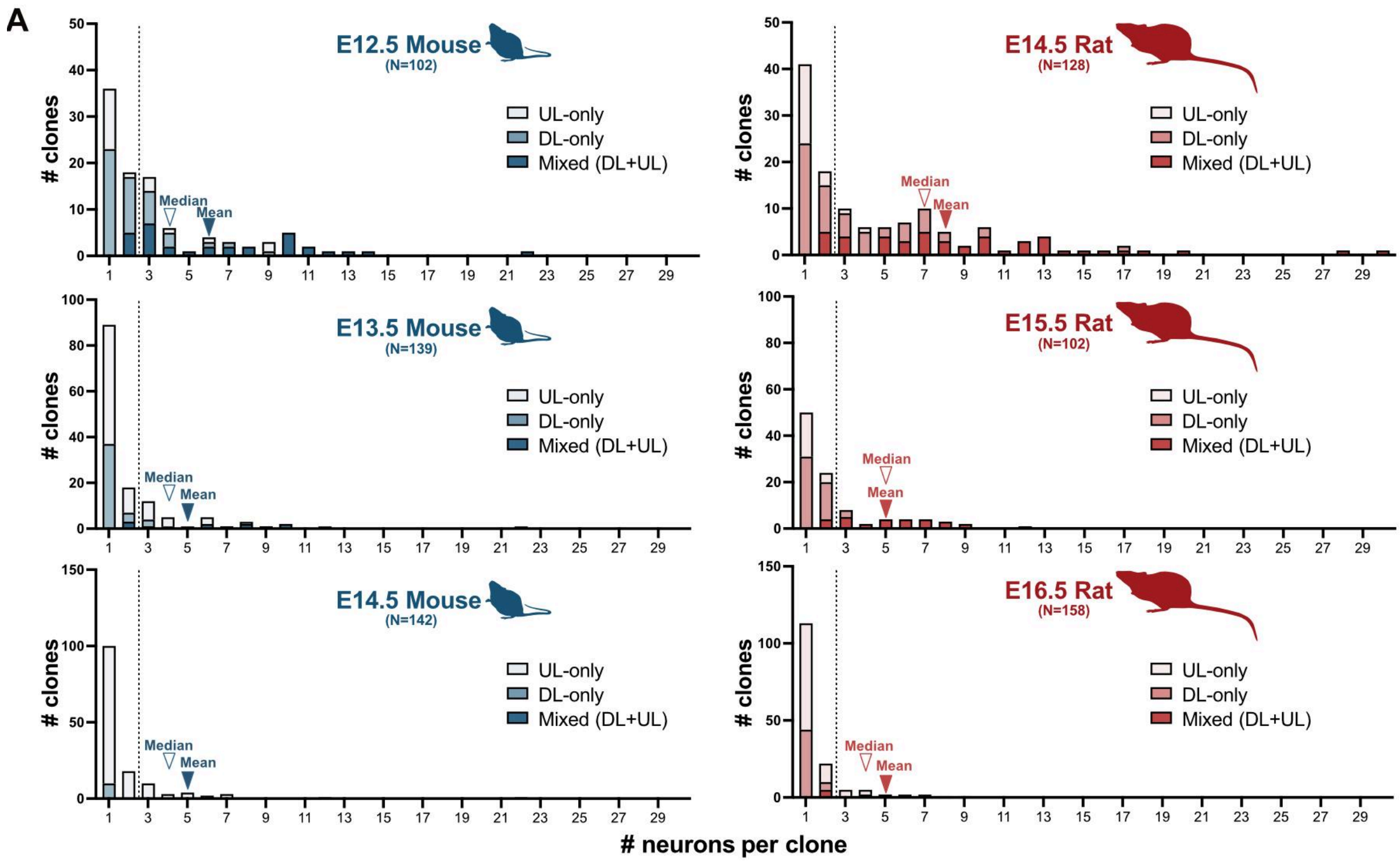

Appendix Figure S5. Clonal analysis with different retroviral injection timings, related to Figure 3

A-F. Distribution of clonal sizes in mice and rats with retroviral injections at different stages. Clone categories are visualized using color intensity.

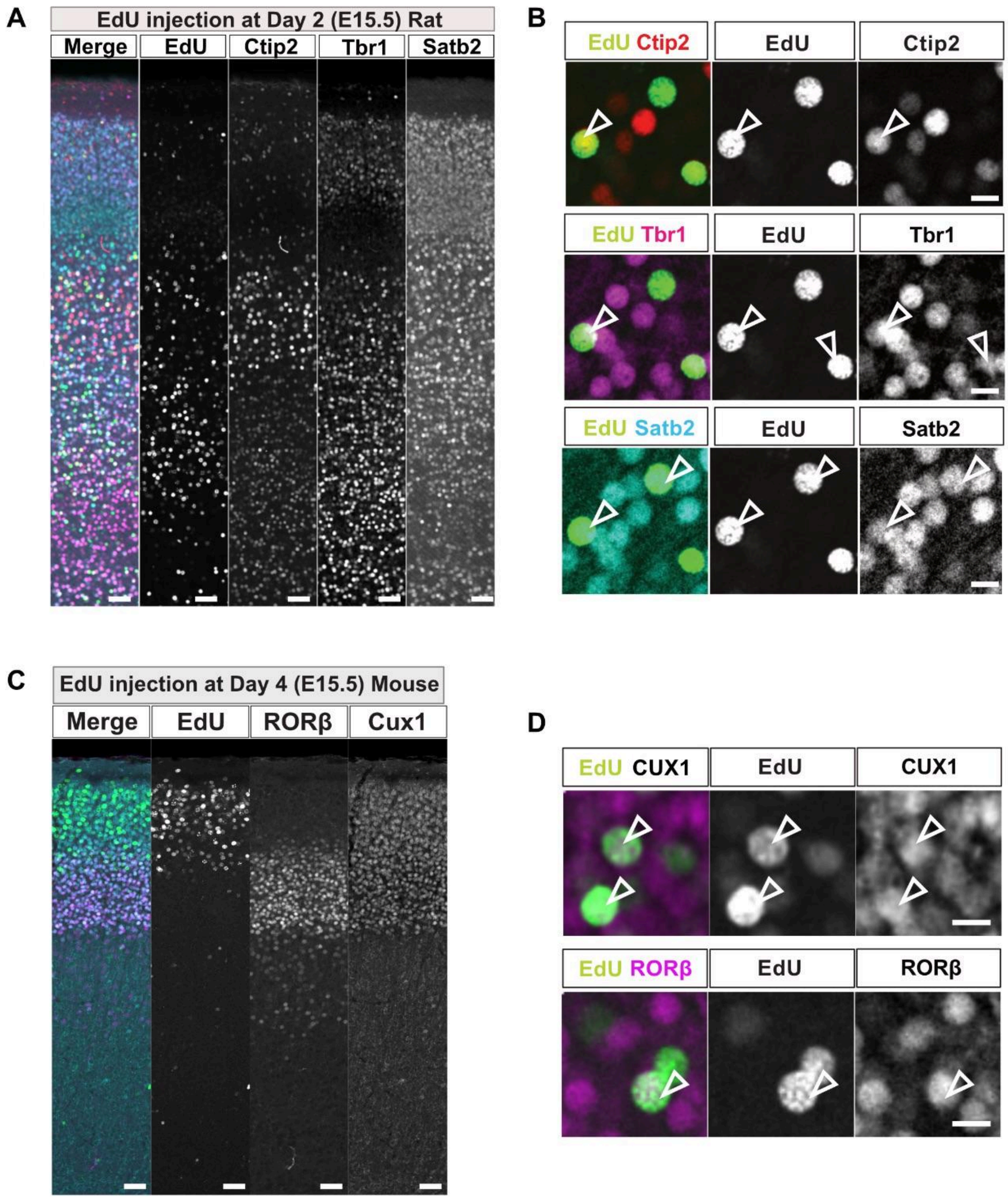

Appendix Figure S6. Marker immunoreactivity of EdU-labeled neurons, related to Figure 4
A-D. Representative images of cortical neurons co-labeled with EdU and subtype-specific markers. Open arrowheads indicate double-positive cells. Scale bars: 50 µm (**A** and **C**), 10 µm (**B** and **D**).

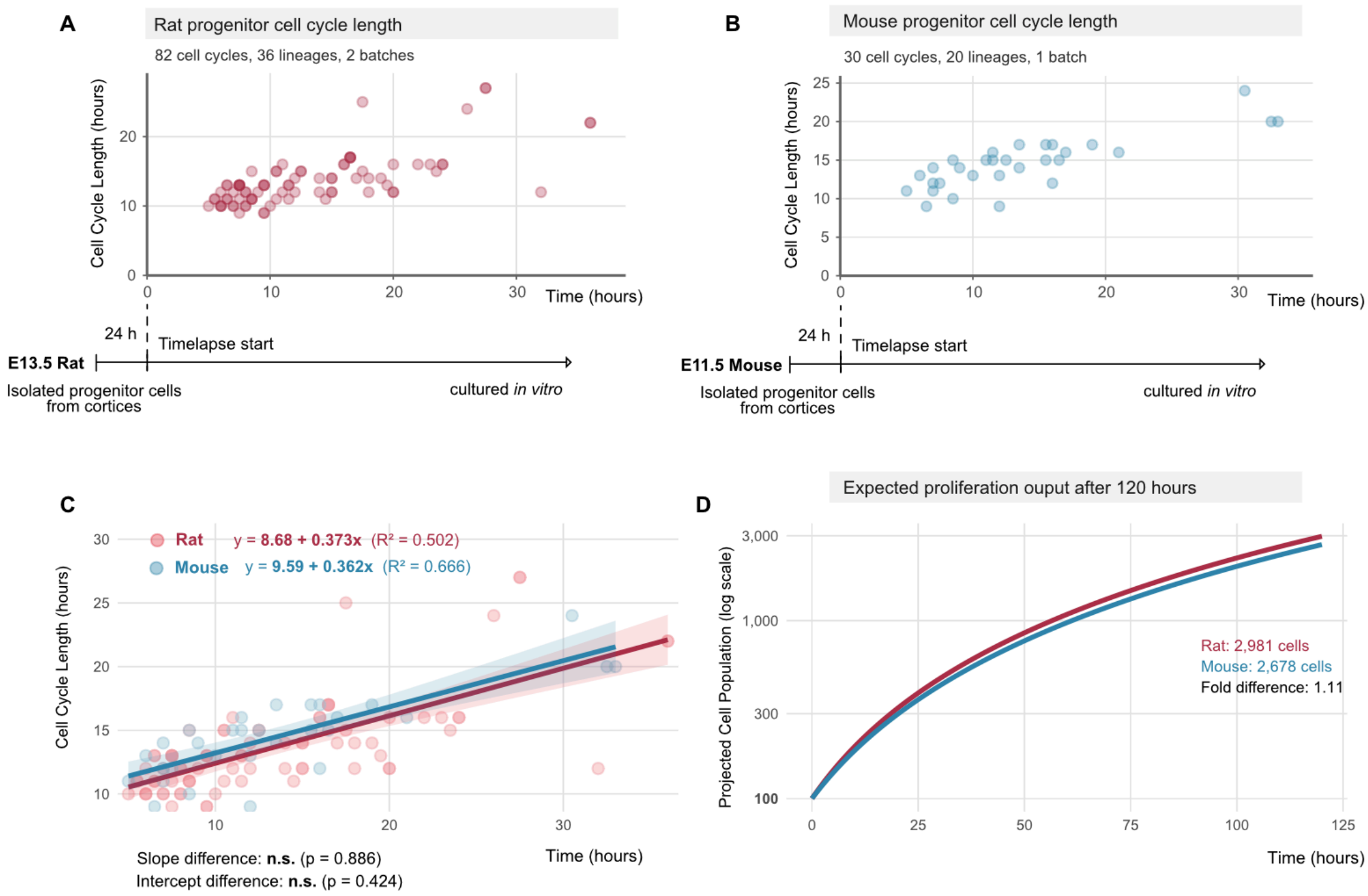

Appendix Figure S7. Cell cycle length of mouse and rat cortical progenitors, related to Figure 5

A-B. Cell cycle lengths of progenitor cells dissociated from rat E13.5 cortices (A) and mouse E11.5 cortices (B). Cells were isolated and labeled with cytosolic GFP for cell tracking. Cells were cultured in DMEM/F-12 supplemented with N2 and B27 (without Vitamin A) and placed in a CO_2 incubator under microscopy. Cell cycle length occurrence time (x-axis) is defined as the midpoint between two consecutive cell division events. Recorded cell cycle number, lineage number (cell division lineage), and batch number are labeled on the plots.

C. Fitting curves of mouse and rat cell cycle length progression. Quadratic and linear fittings were applied, and the model with the higher fit was selected. ANCOVA was used to determine whether slopes or intercepts differed significantly between mouse and rat. Full model as cycle_length ~ time * species was applied. The interaction term (time:species) tests for differences in slopes; the main effect (species) tests for differences in intercepts (after accounting for slope). Slope difference: n.s. (p = 0.886). Intercept difference: n.s. (p = 0.424).

D. Expected proliferation output based on the fitted cell cycle progression curves after 120 hours. Rats are projected to have 1.11-fold more cells than mice in this simple model.

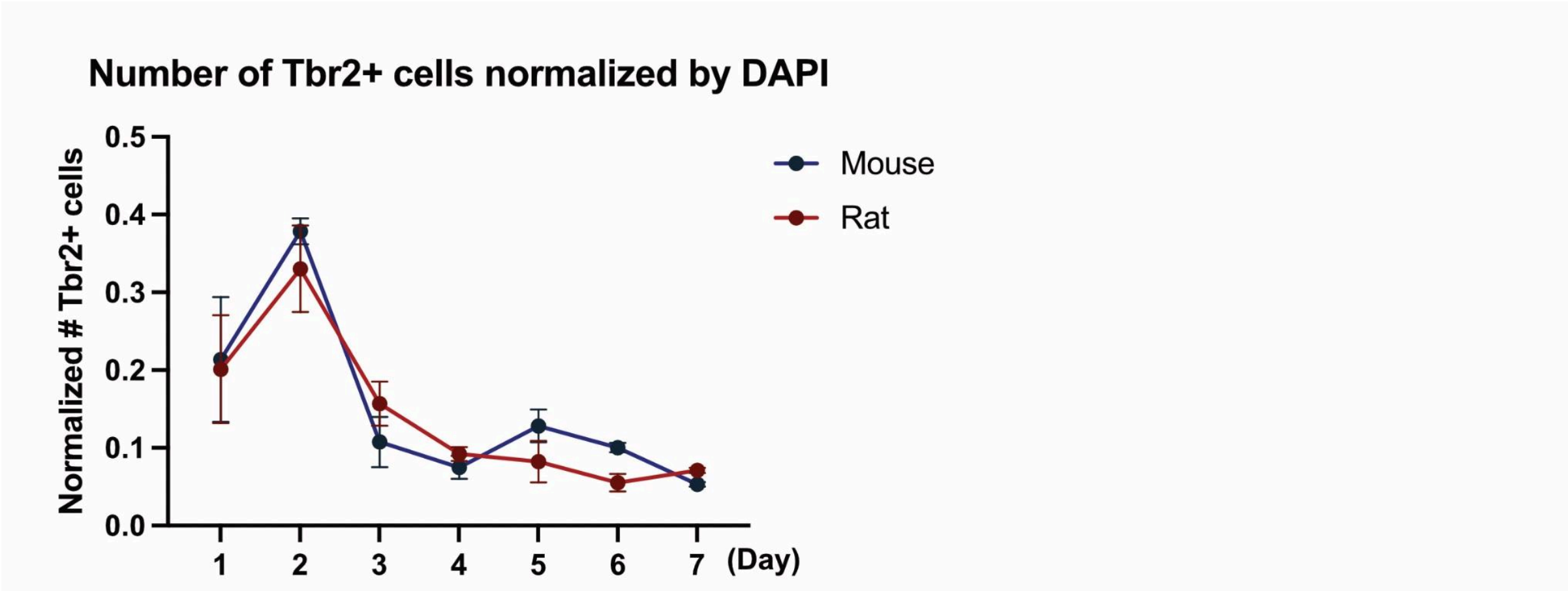

Appendix Figure S8. Temporal changes in the number of Tbr2[+] intermediate progenitor cells in mouse and rat developmental cortex, related to Figure 5

The number of Tbr2[+] cells was quantified daily from Day 1(E12.5 in mice, E14.5 in rats) to Day 7 (E18.5 in mice, E20.5 in rats) . Blue circles indicate mouse samples, and red circles indicate rat samples. Data are presented as mean ± SEM.

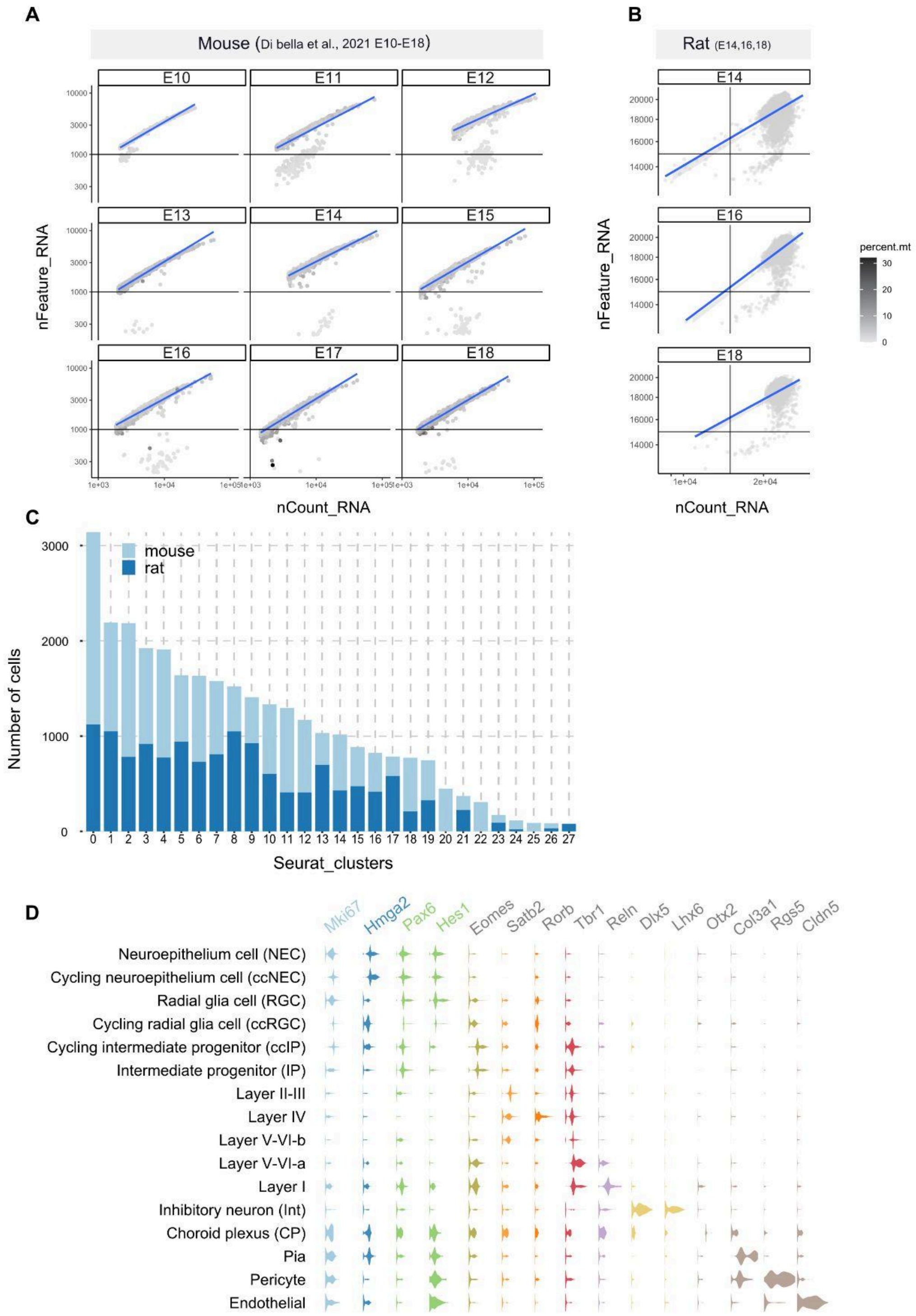

Appendix Figure S9. Single-cell RNA sequencing of mouse and rat cortical cells, related to Figure 6

A-B. Distribution of detected genes, UMI counts, and mitochondrial read percentages in rat (**A**) and mouse (**B**) datasets. **C**. Cell numbers in UMAP clusters for both species. **E**. Expression of canonical markers in annotated cell types.

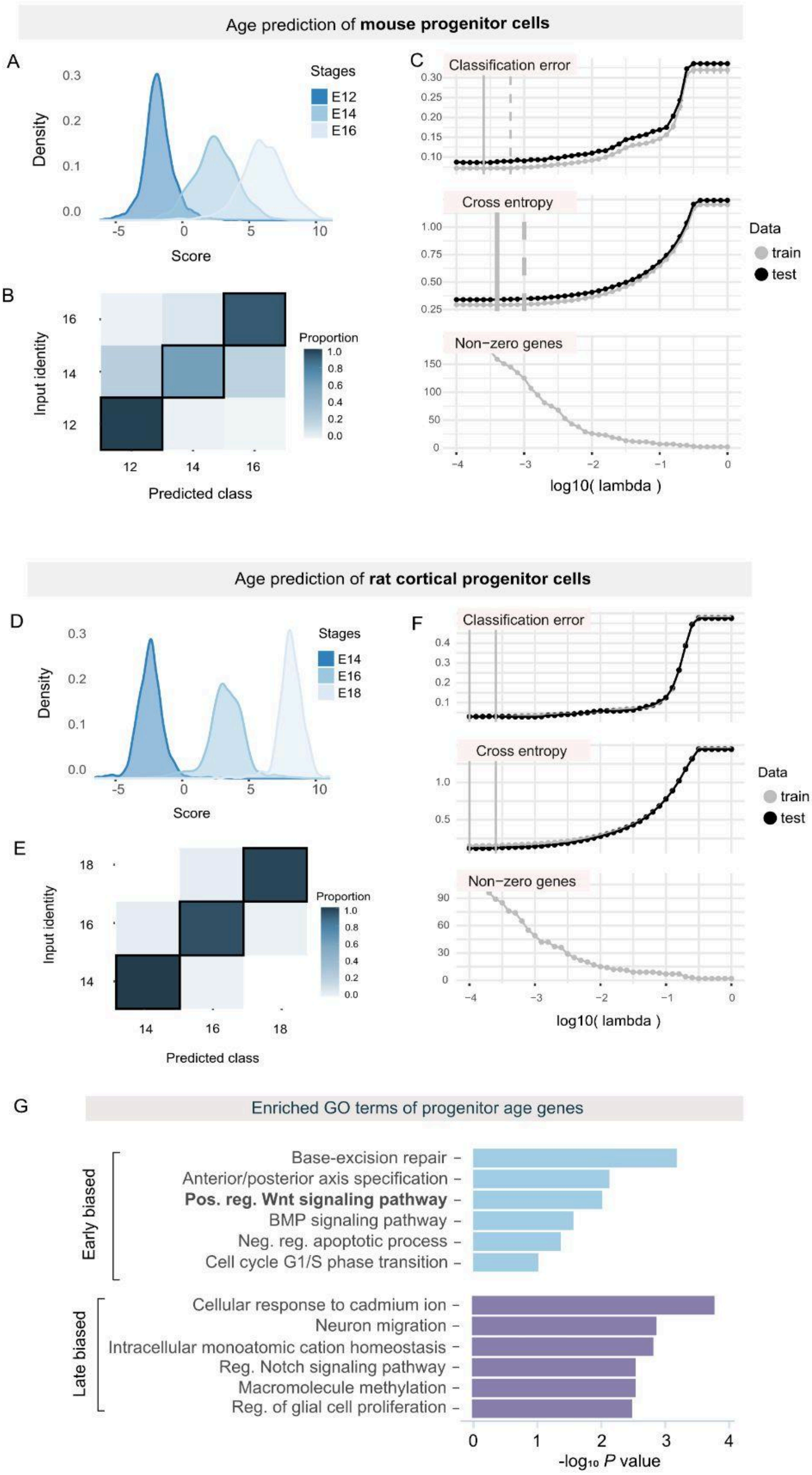

Appendix Figure S10. Identification of progenitor age genes using the ordinal regression model, related to Figure 6

A-F. Expression of progenitor age genes predicts sampling stages of progenitor cells in mice (**A-C**) and rats (**D-F**). **G**. Gene ontology analysis of progenitor temporal (age) genes.

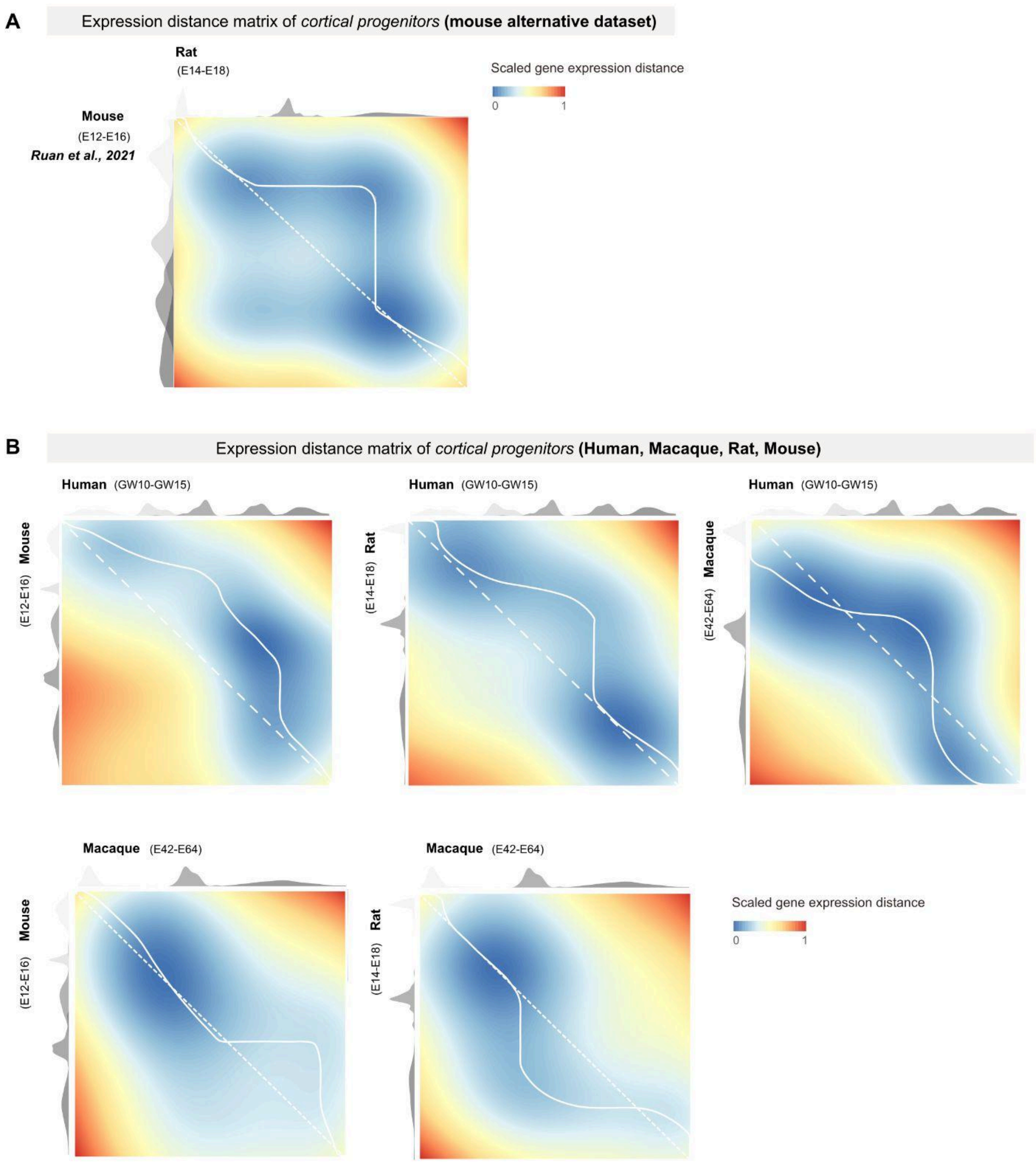

Appendix Figure S11. Gene expression distances in primate and rodent cortical progenitors, related to Figure 6

A. Pairwise gene expression dissimilarity matrix for cortical progenitor cells from rodents (rat and mouse). Mouse cortical cells (E12.5 to E16.5) were sourced from an alternative single-cell dataset (Xiangbin Ruan et al., 2021) to further validate the findings presented in Figure 6. Cells were aligned by pseudotime scores along species-specific trajectories, with the optimal alignment (spanning both trajectories) indicated by the solid white line. Gene expression values were first scaled (0–1) within each trajectory and then compared across species to highlight temporal changes while omitting magnitude differences among datasets. **B.** Pairwise gene expression distances (dissimilarities) among cortical progenitors from human (GW10–GW15), macaque (E42–E64), mouse (E12–E16), ferret (E25–E40), and rat (E14–E18) are shown in the distance heatmap. Distances were calculated using shared highly variable genes (HVGs; n = 826) across all five species, identified via the FindVariableFeatures function with the "vst" method.

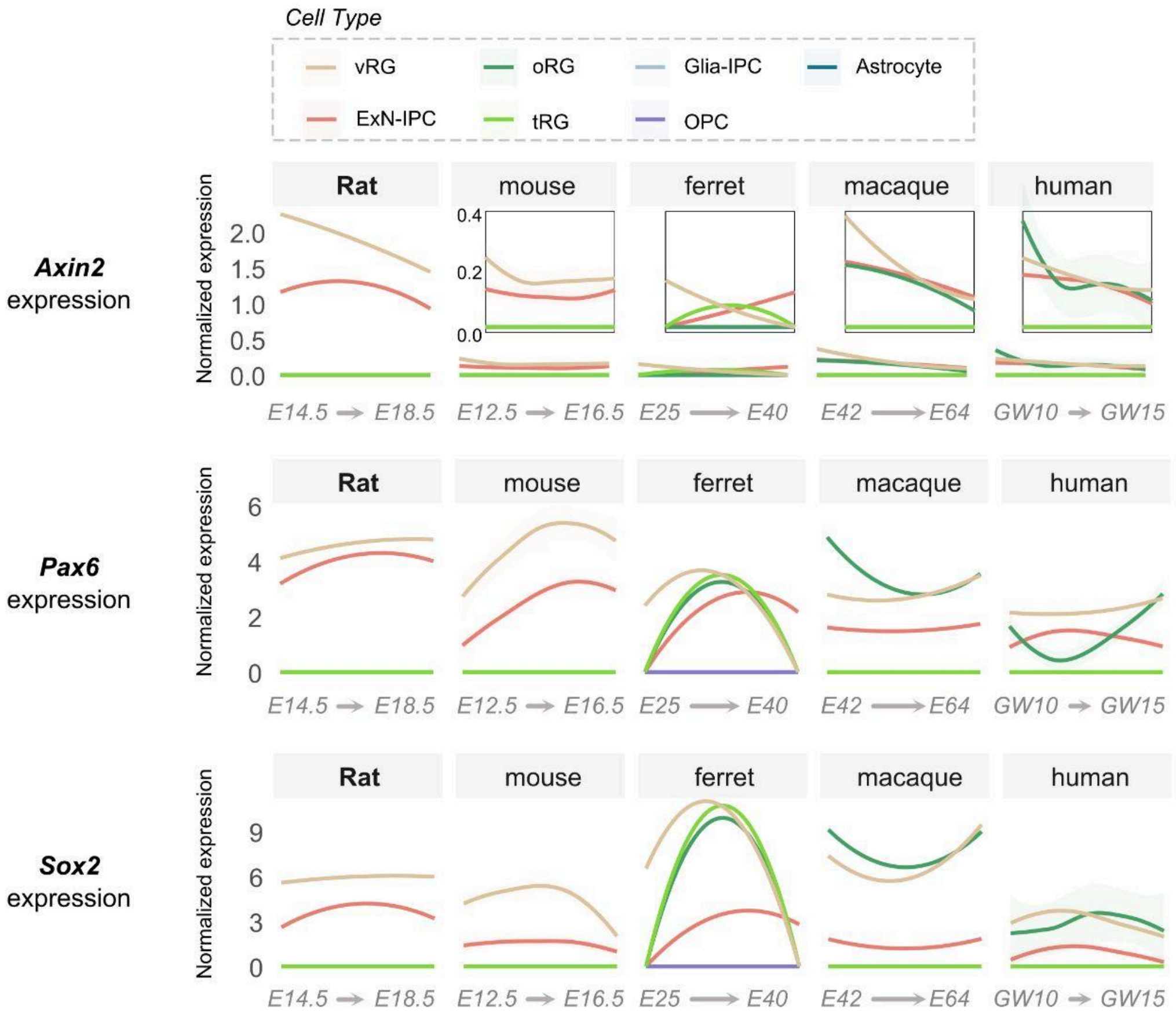

Appendix Figure S12. Significantly elevated expression of *Axin2* in rat RG progenitors compared to other mammals, related to Figure 7

Consistent with the analysis in Figure 7, single-cell RNA sequencing was conducted on rat and other mammalian species (using previously published datasets). Normalized expression values for Axin2, Pax6, and Sox2 across cell types are presented. Expression data were regressed out with sequencing depth and other potential technical variables as described in the Materials and Methods section.

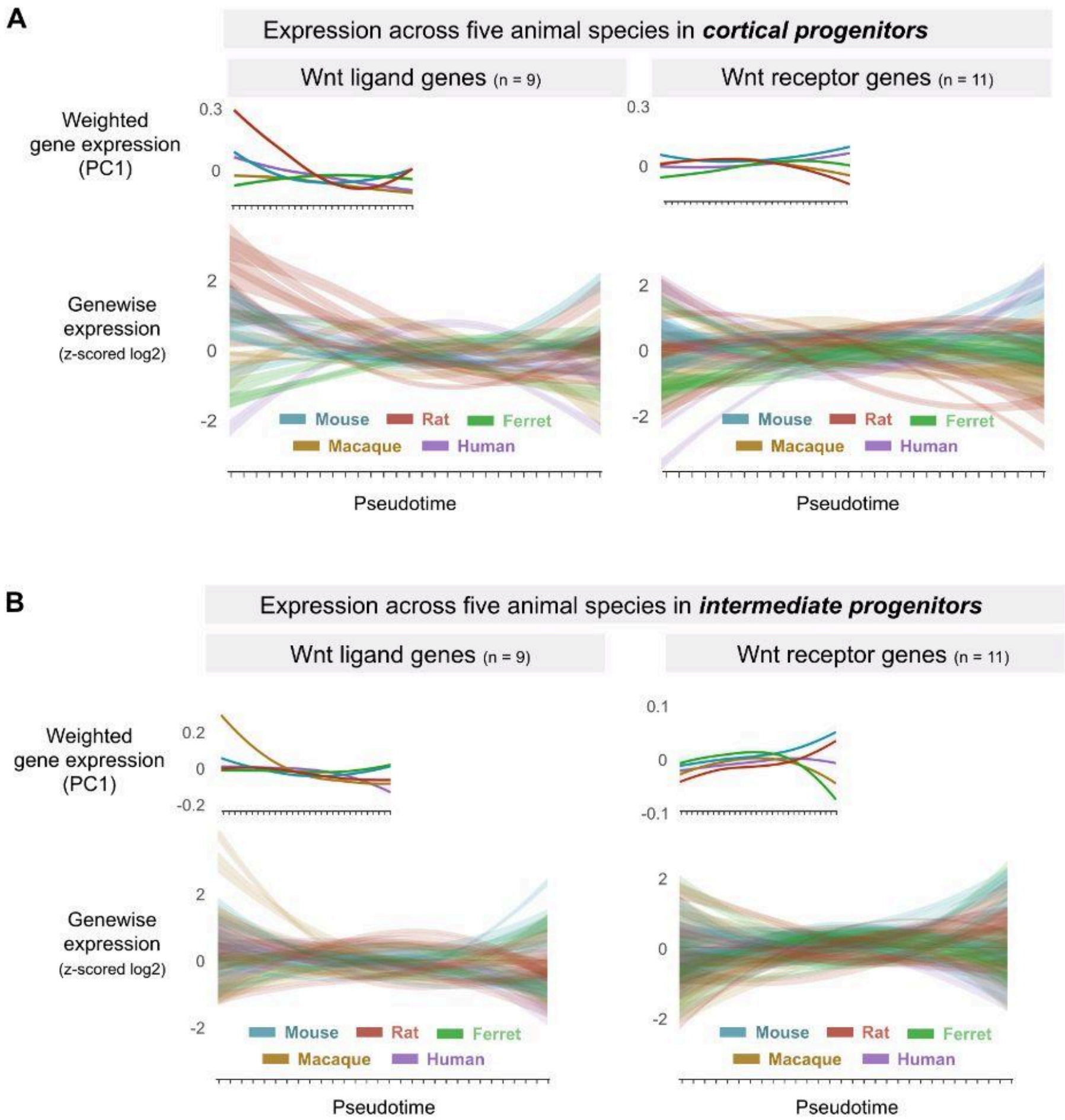

Appendix Figure S13. Wnt ligand and receptor genes among five mammalian species, related to Figure 7

Temporal dynamics and absolute expression of Wnt ligand genes from module 4 (n = 9) and canonical Wnt receptor genes (n = 11) across progenitors in five mammalian species. Gene expression values were log-normalized and z-scored across pseudotime to illustrate temporal dynamics, as described in the Methods section. Module genes weighted summary, representing the first principal component of each gene expression matrix, are displayed in the upper left corner of each plot.

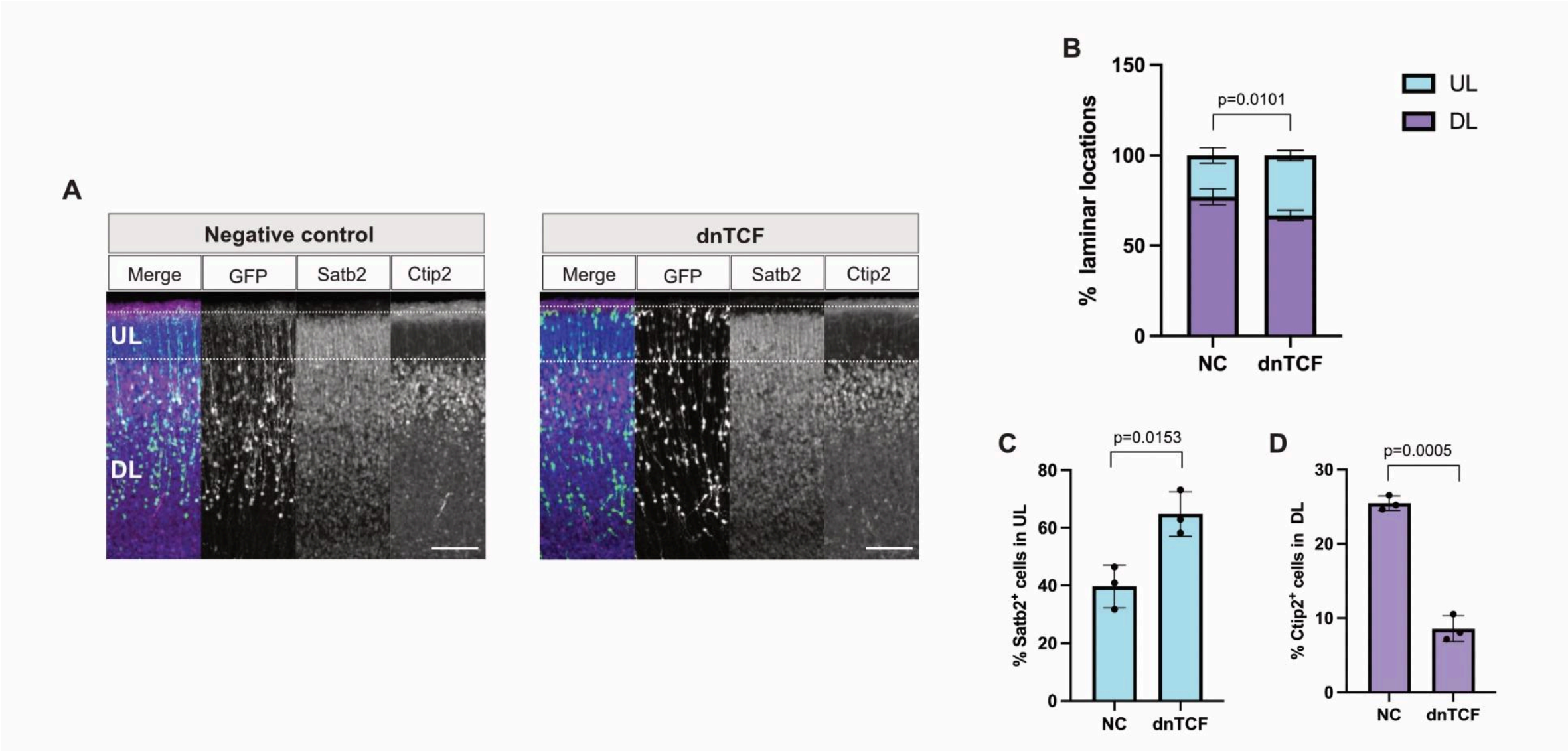

Appendix Figure S14. Inhibition of canonical Wnt signaling by introduction of dnTCF to RG progenitors alters neuronal subtype specification at E20.5, related to Figure 7.

A. Representative coronal cortical sections at E20.5 from negative control (NC) and dnTCF-electroporated at E15.5, immunostained for GFP, Satb2, and Ctip2. Dashed lines indicate the boundaries between UL and DL.

B. Quantification of the laminar distribution of GFP^+ cells in NC and dnTCF conditions. The percentage of cells located in UL and DL is shown.

C-D. Quantification of $Satb2^+$ GFP^+ cells in the UL (C) and $Ctip2^+$ GFP^+ cells in the DL (D) in NC and dnTCF conditions.

Scale bars: 100µm (**A**).
