# Supplementary material for: Interspecific diversity in the neuronal composition of the mammalian cortex arises from heterochrony in neurogenesis
Source: EMBO J. 2026 May 22;45(15):5237–66. doi: 10.1038/s44318-026-00806-z (PMC13434014; doi:10.1038/s44318-026-00806-z)
Supplement: Supplementary file 2 [file 44318_2026_806_MOESM2_ESM.pdf]

THE EMBO JOURNAL

Interspecific diversity in the neuronal composition of the mammalian cortex arises from heterochrony in neurogenesis

Yuki Yamauchi, Xuanhao Sheu, Rafat Tarfder, Takuma Kumamoto, Jun Hatakeyama, Haruka Sato, Pauline Rouillard, Merve Bilgic, Shuto Deguchi, Tomonori Nakamura, Yusuke Kishi, Kazuo Emoto, and Ikuo Suzuki

Corresponding author(s): Ikuo Suzuki (suzuki.ikuo.fbs@osaka-u.ac.jp)

Review Timeline:

Transfer from Review Commons:	31st Jan 26
Editorial Decision:	11th Mar 26
Revision Received:	5th Apr 26
Accepted:	24th Apr 26

Review COMMONS

Editor: Ioannis Papaioannou

Transaction Report: This manuscript was transferred to The EMBO JOURNAL following peer review at Review Commons.

Review #1

1. Evidence, reproducibility and clarity:

Evidence, reproducibility and clarity (Required)

Yamauchi and colleagues explore how species-specific differences in timing of neurogenesis may contribute to cell composition in the mature brain, using rat and mouse cortex as a main model of study. They first estimate and compare among 8 mammalian species the number of cortical neurons corresponding to deep layer (DL) and upper layer (UL) neurons. They find a species-specific relative increase of DL/UL neurons in rats, compared with all other species tested. They then explore the cellular mechanisms underlying these differences in mouse and rat, using retrovirus-based clonal analyses and EdU nuclear labeling, as well as axonal projection retrograde tracing. They conclude that the increased number of DL neurons in the rat is correlated with an increase in the period of DL neuron generation at early stages of corticogenesis. They also report a lack of obvious difference in cell cycle kinetics and indirect vs direct neurogenesis that could explain the DL/UL differences. Finally, they perform comparative scRNAseq analysis in mouse vs rat embryonic cortical cells. This first confirms at the transcriptomic level an apparent prolonged period of early neurogenesis in the rat cortex. Moreover they find among modules of co-expression detectable at these stages an increase in genes corresponding to Wnt signalling, a pathway previously linked to increased self-renewal and delayed differentiation of radial glial progenitors. They thus conclude that the species-differences in neuronal number in the rat is linked to increased Wnt signaling at a critical time of corticogenesis.

Overall this is a thorough and elegant study focused on a timely and interesting topic. The data shown are convincing and carefully interpreted. I have however a couple of comments and questions to make the study fully clear and convincing.

- The authors describe cortical neuronal counts across several mammalian species, which is quite impressive, but the information on the methods of counting is lacking: how representative are the data used / shown; how many individuals / brains / sections were used for each species considered? Much more detailed description of the quantifications should be provicded to judge the validity of this first conclusion.
- The authors use several markers of cortical neuron identity to confirm their neuron number measurements, but from the data shown in Figure 1D,E it seems that only some markers (Satb2) show species-differences while others do not (CTIP2 / Tbr1). How do the authors explain this discrepancy - does this mean that it is mainly Satb2 neurons that are

increased in number? But if so how to explain the relative increase in subcortical projections shown in Figure S7?
- The authors focus their study almost exclusively on somatosensory cortex, but can they comment on other areas (motor, visual for instance)? It would be nice to provide additional comparative data on other areas, at least for some of the parameters examined acros mouse and rat. Alternatively the authors should be more explicit in the abstract and description of the study that it is limited to a single area.
- The authors provide convincing evidence of increased Wnt signaling pathway in the rat. They should show more explicitely how other classical pathways of neurogenic balance / temporal patterning are expressed in their mouse and rat transcriptome data sets. These would include Notch, FGF, BMP, for which all the data should be available to provide meaningful species comparison.
- The alignment of mouse and rat trajectories is very nicely showing a delay at early-mid-corticogenesis. But there is also heterochronic transcriptome at latest stages (end of 5). How can this be interpreted? Does this mean potentially prolonged astrogliogenesis in the rat cortex?
- Figure 7: description implies that module 3 is a subset of module 4, but this is not obvious at all from the panels shown. Please clarify.

2. Significance:

Significance (Required)

The topic of the study if of general interest and original, and the conclusions original and important. The approaches used are state of the art and applied in an elegant fashion to the topic. This study should be of broad interest to developmental neurobiologists, but also developmental biologists interesting in temporal patterning and developmental timing across species.

3. How much time do you estimate the authors will need to complete the suggested revisions:

Estimated time to Complete Revisions (Required)

(Decision Recommendation)

Less than 1 month

4. *Review Commons* values the work of reviewers and encourages them to get credit for their work. Select 'Yes' below to register your reviewing activity at Web of Science Reviewer Recognition Service (formerly Publons); note that the content of your review will not be visible on Web of Science.

Web of Science Reviewer Recognition

No

Review #2

1. Evidence, reproducibility and clarity:

Evidence, reproducibility and clarity (Required)

Summary:

Yamauchi et al. performed a comparative anatomical analysis of the layer architecture in the primary somatosensory cortex across 8 mammalian species. Unlike primates, which show an expansion of upper layers (UL), rodents, especially rats, display a pronounced thickening of deep layers (DL). In this study they focus on comparing rats and mice, given the higher abundance of DL neuron subtypes in rats. Using histological analysis, they showed that rats possess significantly more DL neurons per cortical column than mice, while UL neuron counts remain similar. Clonal lineage tracing showed that rat radial glial (RG) progenitors generate more DL neurons, indicating species-specific differences in progenitor neurogenic activity. Birth dating assays confirmed an extended DL neurogenesis phase in rats, followed by a conserved UL generation phase. Single-cell RNA sequencing further revealed that rats maintain an early progenitor state longer than mice, marked by sustained expression of DL-associated genes. Specifically, rat RG progenitors exhibit prolonged and elevated expression of Wnt signaling genes, particularly Wnt ligands. Comparative analysis of published single-cell RNA-Seq across species highlighted that this extended Wnt-high period in rats is exceptional, suggesting a species-specific extension of a conserved neurogenic program.

Major comments:

This reviewer thinks the topic is exciting, and the experiments elegant, insightful and well described. The paper is well written and follows a very logical flow, the conclusion for each experiment is supported by the data and they are carefully stated. This reviewer really appreciated the summary illustration included as a panel in each figure, they think that this greatly enhanced the clarity and accessibility of the data presented, especially because species comparison can be difficult to follow.

In this reviewer's opinion, there are some aspects of the findings that the authors would need to clarify/address to explain in clarify the phenotype observed and to enhance the overall significance of this very well-made paper:

1. The introduction lacks sufficient background and fails to convey the significance of the study. Specifically, why the research was undertaken, what knowledge gap it addresses, and how the findings could be applied. Addressing these questions already in the introduction would enhance the impact of the work and broaden its readership.

2. In figure 5 the authors conclude that "differences in cell cycle kinetics and indirect neurogenesis are unlikely to be the primary factors driving the species-specific variation in DL neuron production. Instead, the temporal regulation of progenitor neurogenic competence, which determines the duration of the DL production phase, provides a more plausible explanation for the greater number of DL subtypes observed in rats". It is not clear to this reviewer how the authors come to this conclusion. Authors observe a significant proportion of mitotic cells in rat VZ from day 1, and a higher constant proportion of mitotic progenitors in SVZ rats compared to mouse (Figure 5C). This points to an early difference in mitotic progenitors that may also lead to increased IP numbers, and potentially an increased number in DL cells, even before day 1.

In addition, the higher abundance of IPs in the G2/S phase (statistically significant in 4 of the 7 time points) (Figure 5F), would suggest that this difference might play a role in the species-specific variation of DL neuron production. The authors should estimate cell cycle length instead of just measuring proportions to conclude something about cell cycle kinetics. They can then model growth curves to predict the effect caused if there were differences in cell cycle length between equivalent cell types across species.

3. In Figure 6 the authors focus only on the mouse and rat datasets. Given the availability of datasets from primates that the author used already for Figure 7, it would give the reader a broader prospective if also these datasets would be integrated in the analysis done for Figure 6, particularly it would be interesting to integrate them in the pseudotime alignment of cortical progenitor. How do human and/or macaque early and late neurogenic phase would compare to mouse and rat in this model?

4. In Figures 6C and 6D, the authors distinguish between cycling and non-cycling NECs and RGCs. Could the authors clarify the rationale behind making this distinction? Could the authors comment on how they interpret the impact of cycling versus non-cycling states on species-specific non-uniform scaling? Do they consider the observed non-linear correspondences to be driven by differences in cell cycle activity?

5. For the non-uniform scaling in Figure 6F, the authors identify critical inflection points and mention that "the largest delay in rat progenitors occurring where Day 1 and Day 3 progenitors overlapped". It would be good if the authors could discuss what they think all

the inflection points represents. How much can it be explained by the heterogeneity within progenitors per time point? There is a clear higher spread of histograms at days 3 and 5, and the histogram at day 5 almost overlaps with day 1. I wonder if the same conclusion about non-uniform scaling would be detected if the distance matrix was built separately for specific cell types, for example only looking at NECs or RGCs.
6. The authors conclude that the elevated and prolonged expression of Wnt-ligand genes in rat RGs extend the DL neurogenic window and contribute to rat-specific expansion of deep cortical layer. In order to validate this finding it would be good for the authors to perform a perturbation experiment and reduce Wnt signalling/ Axin 2 levels in rats or depleted the Lmx1a and Lhx2 double-positive population.
7. The authors conclude that Wnt signaling is a rat specific effect since they did not observe any clear temporal change in wnt receptors in gyrencephalic species, and only a subset of RG in rats co-express Lmx1a and Lhx2. However, specific Wnt ligands and receptors (Wnt5a, Fzd and Lrp6) seem to be upregulated in human as well (Fig 7G), non RG cells could act as wnt ligand inducers in other species, and it has not been demonstrated that Lmx1a and Lhx2 are the source for Wnt ligand production. I wonder if the authors can completely rule out a role for Wnt in the protracted neurogenesis of other species.

Minor comments:

The RNAscope experiment is currently qualitative. Is it the mRNA copy number per cell equal in both species but more cells are positive in rat, or are there differences in number of mRNA molecules as well? It is not indicated if the RNAscope probes are the same for mouse and rat.

2. Significance:

Significance (Required)

How different species achieve such remarkable differences in brain shape and size remains poorly understood. A critical aspect of this process is the duration of the neurogenic phase: the period during which neural progenitors generate neurons. This phase tends to be extended in species with larger brains and contains multiple neuronal stem cell types in varying proportions. It is thought that this accounts for their increased neuronal numbers. In their search for mechanisms that prolong neurogenesis across species, the authors propose a rat-specific role for Wnt ligands in expanding the neurogenic period in the rat brain. Importantly, they rule out that this mechanism operates in other species, such as primates or ferrets, to achieve similar extensions.

The study is of high quality, incorporating rigorous lineage-tracing experiments in two species and single-cell RNA sequencing. Previous work established a role for Wnt signaling in regulating early neurogenesis in mice. Here, the authors characterize a novel population of radial glial cells (Lmx1a and Lhx2 double-positive) that may explain increased Wnt ligand secretion in rats. However, functional validation of this mechanism is still lacking. To strengthen its evolutionary relevance, it would be important to determine whether similar effects occur during earlier neural stages in other species (such as neuroepithelium thickening), or whether other cell types have co-opted the proposed Lmx1a-Lhx2 regulatory module in other species.

From the perspective of a researcher with a stem cell and developmental background focused on neural evo-devo, this manuscript represents a solid and novel contribution. The proposed model of a rat-specific mechanism for extending the neurogenic phase contrasts with the prevailing concept of convergence in mechanisms underlying species-specific cortical development. This raises intriguing questions about how multiple molecular pathways have been co-opted to achieve similar developmental outcomes. Furthermore, we know very little about what determines the duration of specific developmental processes. This work suggests that extended Wnt signaling may account for prolonged neurogenesis in rats compared to mice. Future studies should aim to validate the proposed rat-specific co-option of an Lmx1a-Wnt ligand cascade in cortical radial glia, potentially through relief of Lhx2-mediated repression of Lmx1a.

3. How much time do you estimate the authors will need to complete the suggested revisions:

Estimated time to Complete Revisions (Required)

(Decision Recommendation)

Between 1 and 3 months

4. *Review Commons* values the work of reviewers and encourages them to get credit for their work. Select 'Yes' below to register your reviewing activity at Web of Science Reviewer Recognition Service (formerly Publons); note that the content of your review will not be visible on Web of Science.

Web of Science Reviewer Recognition

Yes

Review #3

1. Evidence, reproducibility and clarity:

Evidence, reproducibility and clarity (Required)

In this study the authors investigate differences between two closely related species, rats and mice, in terms of cortical development and neuronal composition. They first perform comparative analysis of cortical layers which revealed the density and markers of deep layer neurons of rats is disproportionately larger compared to adult mice. They then use retroviruses for lineage analysis from embryonic stages to P7. They find in general that there are temporal differences in when mice and rats produce upper versus deep layer neurons, with the process being protracted in rats. EdU injections were used to report differences in the timing of cortical neuron generation between species and they note no striking differences in IPs. Sc-sequencing of rat cortices at different stages was then used to measure temporal changes in gene expression and compared to published mouse data. They note that rats have sustained Wnt ligand expression in radial glia highlighting that as a potential mechanism of action.

Major concerns
1. Satb2 is also widely recognized as a deep layer marker. The authors need to perform analysis and quantification in Figs 1 and 4 with other II/III and IV markers such as Cux1 and Rorb.
2. Rats have larger cortices. Therefore, quantification of neurons should also be normalized to cortical thickness in Fig 1E and also represented with individual data points.
3. The clonal analysis in Figs 2 and 3 quantifies GFP and RFP and reports these as neurons. However, without using cell-specific markers, it seems the authors cannot exclude that some progeny are also glia derived from a radial glial progney. I don't expect all experiments to have this but they must have some measures of both populations to address this possibility. This needs to be addressed to build confidence in the conclusion that there is clonal production of neurons.
Related to this, the relationship between position and fate is not always 1 to 1. The data summarized in Fig 2G are based on position and not using subtype markers. They should include assessment of markers as they do in Fig 4.
4. In Fig 5, the authors use PH3 as well as EdU to measure differences in indirect

neurogenesis. Using EdU and Tbr2 they report more dividing IPs. However they need to measure this over the total number of Tbr2 cells as it is not normalized to differences in Tbr2 cells between species. Are there total differences in Tbr2+ cells when normalized to DAPI as well? Moreover, little analyses is performed to measure any impact on radial glia. As no striking differences were observed in IPs this leaves the cellular mechanism a bit unclear and begs the impact on radial glia. Measuring PH3+ cells in VZ and SVZ is not cell specific nor does it yield information to support the prolonged neurogenesis.
5. The sc-seq is done in rat and compared to published mouse data from corresponding stages. They conclude species specific differences in progenitor gene expression. I am unsure how appropriate this is. Are similar sequencing platforms used? Can they find similar results if using multiple dataset? There are other datasets that may be used to validate these findings beyond DiBella et al.
6. Wnt ligand analysis requires validation in situ across developmental stages, to support their conclusions. Ideally they might consider doing some manipulations to provide context to this observation.

Minor concerns
1. Please separate images in Fig 1D it is very strange to have them all on top of each other.
2. Are data in Fig 4E Edu+Tbr1+EdU+? This should be clarified and would be most accurate.
3. Fig 4 graphs only have titles without Y axis. Please adjust location of title or repeat for clarity.
4. Fig 4A implies cumulative incorporation which I don't think is being performed here. They should clarify this in the figure.
5. Fig 5 needs labels for the actual stages assayed, as illustrated in Fig 4A.

2. Significance:

Significance (Required)

Strengths:

The finding that there are differences in cortical composition between rats and mice and that this is linked to prolonged neurogenesis in rats
Use of careful and detailed lineage analysis to define differences in temporal production of neurons
Inclusion of single cell sequencing

Limitations:

Largely descriptive
Requires additional investigation to support some conclusions about neurons
Concerns about inferring too much from single cell sequencing done by the authors but compared to publication

Advance: Finding that there are differences in neurogenesis between closely related species is interesting and provides insight into mechanisms of cortical evolution.

Audience: Evolution, cortical development

Expertise: Cortical development, evolution

3. How much time do you estimate the authors will need to complete the suggested revisions:

Estimated time to Complete Revisions (Required)

(Decision Recommendation)

Between 3 and 6 months

4. *Review Commons* values the work of reviewers and encourages them to get credit for their work. Select 'Yes' below to register your reviewing activity at [Web of Science Reviewer Recognition Service]() (formerly Publons); note that the content of your review will not be visible on Web of Science.

Web of Science Reviewer Recognition

Yes

Full Revision

Manuscript number: RC-2025-03240
Corresponding author(s): Ikuo, Suzuki

[Please use this template only if the submitted manuscript should be considered by the affiliate journal as a full revision in response to the points raised by the reviewers.

If you wish to submit a preliminary revision with a revision plan, please use our "Revision Plan" template. ***It is important to use the appropriate template to clearly inform the editors of your intentions.****]*

1. General Statements [optional]

Overview and Key Findings
We are pleased to submit our revised manuscript, which provides a comprehensive comparative developmental analysis between mice and rats—two species with critical differences in brain architecture. Our study demonstrates that the fine-tuning of brain cell composition is achieved through the precise regulation of developmental speed in neural progenitor cells.

Comprehensive Response to Reviewers
We have taken the feedback from all three reviewers very seriously and have systematically addressed every point raised in their insightful evaluations. This revision is not merely a clarification; it is a profound strengthening of our study. Through extensive additional experiments and data re-analysis, we have:

- **Fully Resolved All Reviewer Concerns:** We provide a point-by-point response demonstrating how each technical and conceptual query has been rigorously addressed.
- **Elucidated Key Molecular Mechanisms:** Specifically, we have now clarified the molecular basis for the rat-specific extension of the early neurogenic period, a point of particular interest to the reviewers.
- **Achieved a New Level of Completeness:** The integration of multi-dimensional analyses has allowed us to transform our initial findings into a definitive, evidence-backed conclusion regarding species-specific developmental timing.

Significance and Impact
As a result of this rigorous revision process, the manuscript is now substantially more robust and complete than the original submission. We are confident that this work provides a major conceptual leap in evolutionary developmental biology and neuroscience.
We firmly believe that our findings will provide exceptional value to the readership of high-impact journals. The clarity of our results and the thoroughness of our responses make this manuscript an ideal candidate for transfer to your premier affiliate journals.

2. Point-by-point description of the revisions

Reviewer #1

Reviewer 1 Point 1- The authors describe cortical neuronal counts across several mammalian species, which is quite impressive, but the information on the methods of counting is lacking: how representative are the data used / shown; how many individuals / brains / sections were used for each species considered? Much more detailed description of the quantifications should be provided to judge the validity of this first conclusion.

Response: We sincerely thank the reviewer for this insightful and constructive suggestion. We agree that the methodological description of our comparative histological analysis, which is the fundamental basis of this study, was insufficient in the original manuscript. Following the reviewer's advice, we have extensively revised the Materials and Methods section entitled "Nissl staining and neuronal cell number count" (**Page 32, Line 15**).

Reviewer 1 Point 2- The authors use several markers of cortical neuron identity to confirm their neuron number measurements, but from the data shown in Figure 1D,E it seems that only some markers (Satb2) show species-differences while others do not (CTIP2 / Tbr1). How do the authors explain this discrepancy - does this mean that it is mainly Satb2 neurons that are increased in number? But if so how to explain the relative increase in subcortical projections shown in Figure S7?

Response: We appreciate the reviewer's insightful comments regarding the marker expression patterns. Upon re-evaluating our data in light of your feedback, we agree that the species differences in deep-layer (DL) markers such as Ctip2 and Tbr1 in the adult stage appear relatively modest compared to the robust differences observed in Satb2 and the projection data shown in **Figure S8**.

To address this point, we have incorporated a comparison between the adult data (**Figure 1**) and our findings from P7 (**Figure S2**). As shown in the revised manuscript, the species differences for all markers are significantly more pronounced at P7 than in the adult. Notably, in the lower layers, rats exhibit a significantly higher number of marker-positive cells across all markers, including those newly added in this revision, compared to mice.

We offer the following interpretation regarding these temporal differences:

1. Developmental Relevance: The marker molecules analyzed are well-established regulators of neuronal subtype fate and projection identity during development. Their critical fate-determining functions are primarily exercised during the migration and maturation phases of nascent neurons.
2. Postnatal Expression Shifts: Whether these molecules maintain functional roles in the fully matured adult brain remains less certain. It is plausible that marker expression may diminish in certain neuronal populations during late postnatal development, leading to the attenuated species differences observed in adults.

Review COMMONS

Consequently, we believe the strong correlation between P7 quantitative data and projection fate provides a biologically sound validation of our hypothesis.
While we have kept the discussion in the main text concise to maintain focus for the general reader, we have provided comprehensive data in **Figure 1** and **Figure S2**. This ensures that the necessary evidence is readily available for specialists interested in these developmental dynamics.

Reviewer 1 Point 3- The authors focus their study almost exclusively on somatosensory cortex, but can they comment on other areas (motor, visual for instance)? It would be nice to provide additional comparative data on other areas, at least for some of the parameters examined across mouse and rat. Alternatively the authors should be more explicit in the abstract and description of the study that it is limited to a single area.

Response: We sincerely appreciate the reviewer's insightful comment. As suggested, we have revised the Abstract to explicitly state that our current analysis is focused on the somatosensory cortex. Furthermore, as demonstrated in **Figure 1B**, we have added a discussion regarding the possibility that the species differences observed in the primary somatosensory cortex may be a general feature shared across the entire cerebral cortex, as follows: "This DL-biased thickening in rats was evident in the primary somatosensory area, but is consistently observed throughout the rostral-caudal cortical regions. (**Page 19, Lines 29-31**)"

Reviewer 1 Point 4- The authors provide convincing evidence of increased Wnt signaling pathway in the rat. They should show more explicitly how other classical pathways of neurogenic balance / temporal patterning are expressed in their mouse and rat transcriptome data sets. These would include Notch, FGF, BMP, for which all the data should be available to provide meaningful species comparison.

Response: We sincerely thank the reviewer for this insightful suggestion. Following your advice, we have newly included comparative data on key signaling pathways essential for cortical development—namely Wnt, FGF, NOTCH, mTOR, SHH, and BMP—across different species. These results are now presented in **Figure S17**. Rat progenitors show comparable patterns to other species for FGF, mTOR, and Notch signaling, but elevated Wnt and BMP expression, especially at early stages. A detailed heatmap of raw Wnt pathway gene expression across species is also included in the same supplementary figure. We believe these additions provide a more comprehensive evolutionary perspective and significantly strengthen our findings.

Reviewer 1 Point 5- The alignment of mouse and rat trajectories is very nicely showing a delay at early-mid-corticogenesis. But there is also heterochronic transcriptome at latest stages (end

of 5). How can this be interpreted? Does this mean potentially prolonged astrogliogenesis in the rat cortex?

Response: We sincerely appreciate the reviewer's insightful comment and the meticulous attention given to our data. Regarding the heterochronic shift observed at Day 5, we agree that this point was not sufficiently addressed in the original manuscript.

We would like to clarify the two primary reasons for this omission, which are inherent to the current study's design:

1. Resolution of Stage Alignment at Temporal Extremes: In our developmental stage alignment analysis, corresponding stages are defined by pairs showing the highest transcriptomic similarity within the sampled range. By definition, the precision of this alignment tends to decrease at the earliest and latest time points of a dataset. Since the "true" biological equivalent might lie outside our sampling window, we must be cautious in interpreting shifts at these temporal boundaries.
2. Difference in Validation Rigor: Our study prioritized the early stages of deep-layer (DL) neuron production. Consequently, we rigorously defined the onset of neurogenesis in rats (Day 1) using multiple independent methods, including clonal analysis, immunohistochemistry, and gene expression. In contrast, Day 5 was defined simply as five days post-initiation of neurogenesis, without equivalent multi-modal validation. Given that our primary focus is the early phase of neurogenesis, the precision of the transition from late neurogenesis to gliogenesis is relatively lower.

For these reasons, we believe that an in-depth discussion of the heterochronic shift at Day 5 might lead to over-interpretation. To reflect this more accurately and avoid misleading the reader, we have revised Figure 6F to de-emphasize the Day 5 shift. In addition, we revised the manuscript as "Importantly, while this analysis identified stage pairs with the highest similarity, the correspondence at the edges of the temporal sampling window is inherently less certain than at the center. Consequently, we focus on the notable reflection point at the center of our dataset. (**Page 13, Lines 37-39**)".

We believe these changes more faithfully represent the biological scope of our data while maintaining the scientific integrity of our primary conclusions.

Reviewer 1 Point 6- Figure 7: description implies that module 3 is a subset of module 4, but this is not obvious at all from the panels shown. Please clarify.

Response: We sincerely appreciate the reviewer's careful reading of our manuscript. As suggested, we have revised **Figure 7** to clarify the hierarchical relationship between Module 3 and Module 4, ensuring that their inclusion is now explicitly presented.

Reviewer #2

Reviewer 2 Point 1. The introduction lacks sufficient background and fails to convey the

significance of the study. Specifically, why the research was undertaken, what knowledge gap it addresses, and how the findings could be applied. Addressing these questions already in the introduction would enhance the impact of the work and broaden its readership.

Response: We sincerely appreciate the reviewer's insightful comment on this point. Our study reports evolutionary insights gained through an unconventional approach: a single-cell level comparison between mice and rats. We agree that clarifying the necessity of this specific approach is crucial for the manuscript. Accordingly, we have added the following two points to the Introduction:

1. At the end of the first paragraph, we emphasized the current lack of research on the evolutionary adaptation of cortical circuits, despite the established functional importance of evolutionarily conserved circuits. (**Page 3, Lines 7-10)**; "Paradoxically, despite the importance of these variations, research has predominantly focused on the conserved aspects of cortical architecture. Consequently, the degree of evolutionary plasticity inherent in these circuits and the cell-intrinsic mechanisms driving their modification remain profoundly enigmatic.")
2. At the end of the third paragraph, we revised and added text (**Page3, Lines 26-27**; "This lack of comparative insight represents a significant gap in our understanding of how conserved developmental programs give rise to species-specific brain architectures.").

Reviewer 2 Point 2. In figure 5 the authors conclude that "differences in cell cycle kinetics and indirect neurogenesis are unlikely to be the primary factors driving the species-specific variation in DL neuron production. Instead, the temporal regulation of progenitor neurogenic competence, which determines the duration of the DL production phase, provides a more plausible explanation for the greater number of DL subtypes observed in rats". It is not clear to this reviewer how the authors come to this conclusion. Authors observe a significant proportion of mitotic cells in rat VZ from day 1, and a higher constant proportion of mitotic progenitors in SVZ rats compared to mouse (Figure 5C). This points to an early difference in mitotic progenitors that may also lead to increased IP numbers, and potentially an increased number in DL cells, even before day 1.
In addition, the higher abundance of IPs in the G2/S phase (statistically significant in 4 of the 7 time points) (Figure 5F), would suggest that this difference might play a role in the species-specific variation of DL neuron production. The authors should estimate cell cycle length instead of just measuring proportions to conclude something about cell cycle kinetics. They can then model growth curves to predict the effect caused if there were differences in cell cycle length between equivalent cell types across species.

Response: We sincerely thank the reviewer for their careful reading of our manuscript and for pointing out the overstatements in our original descriptions. We agree that a more nuanced interpretation of the data was necessary. In response to these constructive suggestions, we have made the following revisions:

1. Refinement of Descriptions: We have revised the text to more accurately reflect our findings, specifically noting that the increase in RG division on Day 1 and IP proliferation throughout the neurogenic period showed a significant trend. These features are now described more fairly and cautiously in the revised manuscript. (**Page 11, Lines 42-46**; “Remarkably, while the temporal dynamics of mitotic density were strikingly conserved between the two species, subtle yet discernible species-specific signatures emerged. Specifically, rats exhibited a higher ratio of mitotic cells in the VZ at the onset of neurogenesis, the precise period when DL subtypes are generated in both species. Further assessment of G2/S-phase cells via pulse-EdU labeling (**Figure 5D, E**) “)
2. Inclusion of Time-lapse Imaging Data: The reviewer is correct that measuring the proportions of M and G2/S phases provides only a limited snapshot of cell cycle dynamics. To gain a more precise insight, we performed primary cultures of neural progenitor cells (NPCs) from Day 1 and conducted live-cell time-lapse imaging. This allowed us to directly quantify the cell cycle duration of mouse and rat NPCs (**Figure S9A-C**).
3. Comparative Analysis and Mathematical Modeling: Our new data revealed that the cell cycle lengths of the two species are remarkably similar, with no significant differences observed under these culture conditions. Furthermore, to validate the impact of these findings on overall brain development, we developed a mathematical model based on our experimental data. This model predicts the total number of cells produced over the five-day neurogenic period, providing a more robust theoretical framework for our conclusions (**Figure S9D**).

We believe these additions significantly strengthen the manuscript and address the reviewer's concerns regarding the physiological relevance of our observations.

Reviewer 2 Point 3. In Figure 6 the authors focus only on the mouse and rat datasets. Given the availability of datasets from primates that the author used already for Figure 7, it would give the reader a broader prospective if also these datasets would be integrated in the analysis done for Figure 6, particularly it would be interesting to integrate them in the pseudotime alignment of cortical progenitor. How do human and/or macaque early and late neurogenic phase would compare to mouse and rat in this model?

Response: We sincerely appreciate the reviewer’s insightful suggestion. In accordance with this comment, we have now incorporated pseudotime alignments of cortical progenitors between primates (human, macaque) and rodents (mouse, rat), presented as pairwise gene expression distance matrices with dynamic time warping in **Figure S13**. These heatmaps illustrate temporal compression or stretching in progenitor gene expression progression across species. Notably, macaque progenitors show no definitive deviations from rodents, whereas human progenitors exhibit distinct protraction relative to rats and even more so to mice. These additions provide a more comprehensive cross-species perspective without altering the study's core conclusions.

Reviewer 2 Point 4. In Figures 6C and 6D, the authors distinguish between cycling and non-cycling NECs and RGCs. Could the authors clarify the rationale behind making this distinction? Could the authors comment on how they interpret the impact of cycling versus non-cycling states on species-specific non-uniform scaling? Do they consider the observed non-linear correspondences to be driven by differences in cell cycle activity?

Response: We are grateful to the reviewer for their insightful observation. We agree that our initial classification of neural progenitor cell (NPC) populations based on proliferation marker expression levels followed a convention used in other studies but was, in the context of this work, unnecessary and potentially misleading. To avoid further confusion and focus on the core biological question, we have re-organized the data by pooling these populations into a single group. Regarding the concern about species differences in cell cycle kinetics, we believe there is no significant divergence between mice and rats that could explain the observed developmental patterns in temporal progression of neurogenesis. This is supported by two lines of evidence:

1. Quantitative analysis of pH3-positive cells (**Figure 5**).
2. New time-lapse imaging data of primary cultured NPCs, which shows no substantial difference in cell cycle length between the two species (**Figure S9**).

These results indicate that the species-specific differences in deep-layer (DL) neuron production are not driven by cell division kinetics. Consequently, we conclude that the non-linear developmental progression of NPCs occurs independently of cell cycle regulation.

Reviewer 2 Point 5. For the non-uniform scaling in Figure 6F, the authors identify critical inflection points and mention that "the largest delay in rat progenitors occurring where Day 1 and Day 3 progenitors overlapped". It would be good if the authors could discuss what they think all the inflection points represents. How much can it be explained by the heterogeneity within progenitors per time point? There is a clear higher spread of histograms at days 3 and 5, and the histogram at day 5 almost overlaps with day 1. I wonder if the same conclusion about non-uniform scaling would be detected if the distance matrix was built separately for specific cell types, for example only looking at NECs or RGCs.

Response: We sincerely appreciate the reviewer's insightful perspective on this point. In alignment with the suggestions from both this reviewer and Reviewer 1 (Point 5), we have updated the manuscript to discuss all identified inflection points. Specifically, we have clarified why our discussion focuses on the correspondence between Mouse D1 and Rat Day 3.
A recognized limitation of our current analytical approach is that it identifies the closest matching expression profiles within the specific timeframes sampled for each species. For stages at the beginning or end of our sampling window, the "true" corresponding stage in the other species may lie outside our sampled range, which naturally limits the strength of any conclusions regarding those boundary points. Consequently, while we can confidently confirm the correspondence between Mouse Day 1 and Rat Day 3—both of which sit centrally within our

sampled window—we have intentionally avoided over-interpreting data near the temporal boundaries.

Regarding the cell types analyzed, this specific analysis was conducted exclusively on NECs and RGs (now shown in **Figure 6F**). Extensive prior research (Susan McConnell lab, Sally Temple lab, Fumio Matsuzaki lab, Dennis Jabaudon lab, and more) has established that the time-dependent mechanisms governing the fate determination of cortical excitatory neuron subtypes are encoded within RGs. Therefore, we focused our investigation on these lineages and did not include other cell types in this study. We believe this focused approach maintains the highest degree of biological relevance for our conclusions.

Reviewer 2 Point 6. The authors conclude that the elevated and prolonged expression of Wnt-ligand genes in rat RGs extend the DL neurogenic window and contribute to rat-specific expansion of deep cortical layer. In order to validate this finding it would be good for the authors to perform a perturbation experiment and reduce Wnt signalling/ Axin 2 levels in rats or depleted the Lmx1a and Lhx2 double-positive population.

Response: We thank the reviewer for this insightful suggestion. We agree that providing direct experimental evidence is crucial to demonstrating that elevated Wnt signaling in RG progenitors drives the production of DL subtype neurons in rats. To address this, we performed a functional intervention on Day 3, a stage when Wnt signaling (indicated by *Axin2* expression) is significantly higher in rats than in mice (**Figure 7C, D**). By introducing a dominant-negative form of TCF7L2 (dnTCF7L2) to inhibit Wnt signaling specifically in RG progenitors, we tracked the fate of the resulting neurons (**Figure 7I, J**). Our results showed a clear reduction in the proportion of DL neurons, accompanied by a reciprocal increase in upper-layer (UL) neurons. These findings demonstrate that maintained high levels of Wnt signaling are essential for the prolonged neurogenic capacity for DL neurons in rats. This new data has been incorporated into **Figure 7**.

Reviewer 2 Point 7. The authors conclude that Wnt signaling is a rat specific effect since they did not observe any clear temporal change in wnt receptors in gyrencephalic species, and only a subset of RG in rats co-express Lmx1a and Lhx2. However, specific Wntligands and receptors (Wnt5a, Fzd and Lrp6) seem to be upregulated in human as well (Fig 7G), non RG cells could act as wnt ligand inducers in other species, and it has not been demonstrated that Lmx1a and Lhx2 are the source for Wntligand production. I wonder if the authors can completely rule out a role for Wnt in the protracted neurogenesis of other species.

Response: We sincerely appreciate the reviewer's insightful and broad perspective regarding Wnt signaling dynamics across diverse species. In this study, our primary focus was to elucidate the specific mechanisms underlying the differences between mice and rats. Consequently, we did not initially explore Wnt dynamics in other species or their roles in developmental timing in great depth in the original manuscript. We fully acknowledge that lineage-specific adaptations

occur at the individual gene level; for instance, Silver and colleagues have reported that human-specific upregulation of Wnt receptor gene FZD8 modulates neural progenitor behavior (Boyd et al., Current Biology 2008, Liu et al., Nature 2025). However, our comparative analysis of five mammalian species—carefully aligned by developmental stage—reveals a distinct global trend. While individual gene variations exist like human FZD8, the expression levels of multiple Wnt-related genes, particularly ligands, are markedly higher in rats than in the other four species.

Following the reviewer's insightful suggestion, we examined the potential role of *Lmx1a* in activating Wnt ligand transcription in rat cortical progenitors by analyzing their expression correlation at the single-cell level. Our analysis revealed that several Wnt ligand genes are co-expressed with *Lmx1a* with a remarkably strong positive correlation. While we have not yet experimentally demonstrated the direct transcriptional activation of Wnt ligands by *Lmx1a* in these cells, this robust correlation at single-cell resolution strongly suggests that *Lmx1a* regulates Wnt ligand expression. These new findings are now included in **Figure 7** and **Figure S16**, and the corresponding results section (**Page 15, Lines 42-44**) has been revised accordingly.

Reviewer 2 Point 8 Minor comments:
The RNAscope experiment is currently qualitative. Is it the mRNA copy number per cell equal in both species but more cells are positive in rat, or are there differences in number of mRNA molecules as well? It is not indicated if the RNAscopeprobes are the same for mouse and rat.

Response: We sincerely thank the reviewer for this insightful suggestion. Following the comment, we performed RNAscope analysis for *Axin2* in both mice and rats and quantified the results (now included in **Figure 7D**). The new data successfully validate the species differences initially observed in our scRNAseq analysis: specifically, the period of high-level *Axin2* expression is significantly extended in rats compared to mice. These findings provide histological evidence that reinforces our conclusions regarding the distinct temporal dynamics between the two species.

Regarding probe design, the *Axin2* RNAscope probes target conserved and corresponding sequences between mouse and rat, with species-specific probes optimized for each organism to ensure maximal specificity and sensitivity. We have updated the Methods section ("Fluorescent in situ hybridization with RNAscope") to include these details.

Reviewer #3

Reviewer 3 Point 1. Satb2 is also widely recognized as a deep layer marker. The authors need to perform analysis and quantification in Figs 1 and 4 with other II/III and IV markers such as Cux1 and Rorb.

Response: We thank the reviewer for their insightful comments regarding the marker specificity. We fully agree that while Satb2 is a robust marker for callosal projection identity, its broad

distribution across both deep and upper layers limits its utility as a layer-specific marker. As the reviewer suggested, Cux1 (Layers 2/3) and Rorb (Layer 4) are indeed superior markers for defining laminar identity.

To address this, we have incorporated new immunohistochemical data for these markers in both the quantification of somatosensory cortical neurons (**Figure S2**) and the birth-dating analysis (**Figure 4**).

Our new findings are as follows:

1. Layer Quantification (**Figure S2**): By utilizing Cux1 and Rorb as more specific upper-layer (UL) markers, we confirmed that there are no significant differences in the number of these neurons between mice and rats.
2. Birth-dating Analysis (**Figure 4**): These markers allowed us to more precisely define the timing of Cux1/Rorb-positive cell generation, revealing subtle but important differences between the two species.

While these additions do not alter the fundamental narrative of the original manuscript, they have significantly enhanced the precision and rigor of our analysis. We are grateful to the reviewer for guiding us toward this more robust validation.

Reviewer 3 Point 2. Rats have larger cortices. Therefore, quantification of neurons should also be normalized to cortical thickness in Fig 1E and also represented with individual data points.

Response: We sincerely appreciate the reviewer's constructive suggestion. We agree that normalizing the number of cortical neurons by thickness provides a more rigorous comparison. Accordingly, we have calculated the neuronal density (cell count per unit thickness) for Tbr1- and Ctip2-positive cells and included these data in **Figure S2C**. Our analysis confirms that these populations are distributed at a significantly higher density in mice compared to rats. Furthermore, we have updated the visualization in **Figure 1E** to display individual data points, ensuring full transparency of the underlying distribution. We believe these revisions, prompted by the reviewer's insight, have substantially strengthened the clarity and persuasiveness of our manuscript.

Reviewer 3 Point 3. The clonal analysis in Figs 2 and 3 quantifies GFP and RFP and reports these as neurons. However, without using cell-specific markers, it seems the authors cannot exclude that some progeny are also glia derived from a radial glial progeny. I don't expect all experiments to have this but they must have some measures of both populations to address this possibility. This needs to be addressed to build confidence in the conclusion that there is clonal production of neurons.

Related to this, the relationship between position and fate is not always 1 to 1. The data summarized in Fig 2G are based on position and not using subtype markers. They should include assessment of markers as they do in Fig 4.

Response: We sincerely thank the reviewer for this insightful comment. We agree that a clear definition of cell types is essential for the accuracy of clonal analysis.

In this study, we primarily identified neurons based on their distinct morphological characteristics and performed measurements specifically on these cells. To validate this approach, we confirmed that the vast majority of cells identified as neurons were positive for NeuN and cortical excitatory neuron markers, while remaining negative for glial markers such as Olig2 and SOX9. (Notably, at postnatal day 7, most cells in the glial lineage exist as undifferentiated Olig2-positive progenitors). These observations support our conclusion that the cells analyzed based on morphology are indeed cortical excitatory neurons.

As the reviewer rightly pointed out, evaluating cell composition using fate-specific marker expression is the ideal approach. However, our current experimental setup required multiple fluorescence channels for DAPI staining (to assess tissue architecture) and immunostaining for GFP and RFP (to identify labeled clones). Due to these technical constraints regarding available detection channels and host species compatibility, we relied on morphological criteria for the primary analysis.

To address this concern and ensure the reliability of our findings, we performed additional analyses using a subset of samples. By co-staining retrovirally labeled neurons with cell-fate markers, we obtained results consistent with our other data (**Figures 1 and 4**) regarding laminar position and marker expression. Based on this consistency, we are confident that our classification based on morphology and laminar position does not alter the fundamental conclusions of this study.

Reviewer 3 Point 4. In Fig 5, the authors use PH3 as well as EdU to measure differences in indirect neurogenesis. Using EdU and Tbr2 they report more dividing IPs. However they need to measure this over the total number of Tbr2 cells as it is not normalized to differences in Tbr2 cells between species. Are there total differences in Tbr2+ cells when normalized to DAPI as well? Moreover, little analyses is performed to measure any impact on radial glia. As no striking differences were observed in IPs this leaves the cellular mechanism a bit unclear and begs the impact on radial glia. Measuring PH3+ cells in VZ and SVZ is not cell specific nor does it yield information to support the prolonged neurogenesis.

Response: We sincerely thank the reviewer for this insightful suggestion. We agree that quantifying Tbr2+/EdU+ double-positive cells alone was insufficient to fully capture the IP dynamics. Following the reviewer's advice, we have now quantified the total population of Tbr2+ cells, normalized to the number of DAPI-stained nuclei. This new analysis reveals that mice and rats exhibit nearly indistinguishable temporal dynamics (**Figure S10**). When integrated with the original Tbr2+/EdU+ data in **Figure 5**, these findings suggest that rats maintain a slightly higher IP pool throughout the neurogenic period. This implies that the increased neuronal production in rats is not restricted to a specific phase, but rather occurs consistently across all developmental stages. We believe these additional data significantly strengthen our conclusions.

Reviewer 3 Point 5. The sc-seq is done in rat and compared to published mouse data from corresponding stages. They conclude species specific differences in progenitor gene expression. I am unsure how appropriate this is. Are similar sequencing platforms used? Can they find similar results if using multiple dataset? There are other datasets that may be used to validate these findings beyond DiBella et al.

Response: We sincerely thank the reviewer for this insightful comment. We agree that establishing the validity of our analytical approach is crucial for the reader's confidence in our findings. To address this, we have explicitly stated in the revised manuscript that both our rat scRNAseq data and the publicly available datasets were generated using consistent experimental platforms. This ensures that the integration process is technically sound.

Revised text (**Page 13, Lines 16-18**): "After quality control, we integrated these profiles with previously published mouse cortical cell data from corresponding neurogenic stages, which is prepared using the consistent platform with ours (35) (**Figure S11**)."

Furthermore, to ensure the robustness of our comparative analysis, we have incorporated an additional independent dataset (Ruan et al., PNAS 2021) in addition to the Di Bella et al. Nature 2021 data used in the original manuscript. We confirmed that the results obtained using this second dataset are highly consistent with our initial findings, further validating our conclusions across different studies (**Figure S13A**).

Reviewer 3 Point 6. Wnt ligand analysis requires validation in situ across developmental stages, to support their conclusions. Ideally they might consider doing some manipulations to provide context to this observation.

Response: We sincerely thank the reviewer for these insightful suggestions. We agree that validating the spatial expression patterns of Wnt ligands and confirming their expression in rat-specific RG, as suggested by our scRNAseq data, is crucial for strengthening our conclusions.

Regarding the expression of Wnt3a, a key ligand in cortical development: although immunohistochemical analysis clearly identified Wnt3a expression in the cortical hem, the expression levels in RG within the cortical area were substantially lower than those in the hem, making definitive visualization challenging. To complement these findings and provide more robust evidence, we performed the following additional experiments:

1. Validation of Wnt signaling levels: Using RNAscope-based *in situ* hybridization for *Axin2*, we successfully confirmed the elevated Wnt signaling levels in rat-specific RG (**Figure 7C, D**), consistent with our scRNAseq findings.
2. Elucidating strikingly high correlated expressions of *Lmx1a* and Wnt ligand genes in the rat cortical progenitors in our scRNAseq dataset (**Figure S16B**).
3. Functional analysis: To test the functional significance of this signaling, we inhibited Wnt signaling by electroporating dominant-negative TCF7L2 into rat RG at E15.5. This manipulation resulted in a subtype shift of the generated neurons toward an upper-layer identity (**Figure 7I, J**).

These new results demonstrate that the rat-specific extension of high Wnt signaling levels serves as a fundamental mechanism for the prolonged production of deep-layer (DL) neurons. We are grateful to the reviewer for these suggestions; these additional data have significantly strengthened our core argument that the heterochronic regulation of Wnt signaling states drives the evolution of cortical neuronal composition.

Reviewer 3 Point 7 Minor concerns-1
Please separate images in Fig 1D it is very strange to have them all on top of each other.

Response: We sincerely thank the reviewer for this suggestion. As requested, we have provided individual channel images alongside the merged multicolor panels. We agree that this modification significantly enhances the clarity of our data and makes the results much easier to interpret.

Reviewer 3 Point 8 Minor concerns-2
Are data in Fig 4E Edu+Tbr1+EdU+? This should be clarified and would be most accurate.

Response: We appreciate the reviewer's suggestion. We added the label of Y axes of the plots in **Figure 4E-K**. The procedure of cell count in these analyses are documented in the caption of **Figure 4E-K**, "Normalized counts of neurons colabeled for EdU and projection-specific markers, relative to the peak of EdU+ and marker+ cells.".

Reviewer 3 Point 9 Minor concerns-3
Fig 4 graphs only have titles without Y axis. Please adjust location of title or repeat for clarity.

Response: We thank the reviewer for this helpful suggestion. To clarify the definition of the Y-axis, we have now added a descriptive label to the axis in the revised figure.

Reviewer 3 Point 10 Minor concerns-4
Fig 4A implies cumulative incorporation which I don't think is being performed here. They should clarify this in the figure.

Response: We appreciate the reviewer's insightful comment. To avoid any potential misunderstanding regarding the additivity of the effect, we have revised the illustration in **Figure 4A** for greater clarity.

Reviewer 3 Point 11 Minor concerns-5
Fig 5 needs labels for the actual stages assayed, as illustrated in Fig 4A.

Response: We thank the reviewer for this helpful suggestion. Following your comment, we have added the developmental stage information (expressed as embryonic days) for both mice and rats in the revised manuscript.

1st Editorial Decision 11th Mar 2026

Dear Dr. Suzuki,

Thank you for the transfer of your revised manuscript (EMBOJ-2026-123747-T) to The EMBO Journal from Review Commons, where the original version of your work had previously been peer-reviewed. Your revised manuscript and your point-by-point response to the referee comments have now been seen by the three original referees, and we have received their reports, which you can find appended below.

As you will see, all three referees are very satisfied with the revision, pointing out the high novelty and significance of the work, and explaining that their initially raised concerns have adequately and sufficiently been addressed. They all recommend publication of the work in The EMBO Journal, with only two minor remaining points raised by referee #3. We kindly request that you address these two points in a final revision, as well as in a point-by-point response to the referees' comments. In particular:
- please include the day numbers in Figure 4A as per the referees' suggestion
- please also address the second point of referee #3; no more experimental analyses will be required for the publication of your work in The EMBO Journal, but if you decide not to present more conclusive data, please take the suggestion of the referee into account and tone down the conclusions to more accurately interpret the currently available data in a balanced manner.

From the editorial side, there are also a few changes we need you to make in the final version of your manuscript, before we can move forward with its publication in The EMBO Journal:

- Please note that an institutional e-mail address must be provided for the corresponding author, both in the Author's profile in our manuscript handling system and on the title page of the revised manuscript.

- Please remove the Figures from the main manuscript file and upload the main and EV Figures (please see below for more information) separately, as individual, production-quality Figure files. Their legends should remain in the main manuscript file, following the list of References.

- Please note that in EMBO Press journal, Supplementary Figures have been replaced by Expanded View (EV) Figures and/or Appendix Figures. Expanded View (EV) files are collapsible/expandable online. A maximum of typically 5 EV Figures can be typeset. EV Figures should be cited as "Figure EV1, Figure EV2" etc. in the text, and their respective legends should be included in the manuscript file after the legends of regular figures. See detailed instructions regarding Expanded View files here: https://link.springer.com/journal/44318/submission-guidelines#cms-Expanded-View-data.

- For the figures that you do NOT wish to display as Expanded View Figures, they should be bundled together with their legends in a single PDF file called "Appendix", which should start with the heading "Appendix for:", followed by the manuscript's title and a Table of Contents (including page numbers) on its title page. Appendix Figures should be referred to in the main text as: "Appendix Figure S1, Appendix Figure S2" etc. Please find more information in our Author Guidelines: https://link.springer.com/journal/44318/submission-guidelines#cms-Expanded-View-data.

- "Supplementary" should not be used in the revised manuscript or its related files.

- Please change heading "Data, Code and Materials availability" to "Data Availability" and make sure that the deposited data to NCBI GEO database will be publicly available at the time of publication. Please also add in this statement the specific URL to this dataset. If new code was generated to analyse the data in this study, we kindly request that it also be made publicly available, and that the access information be included in this same Data availability section.

- Please rename heading "Competing interests" to "Disclosure and competing interests statement".

- The author contributions statement should be removed from the manuscript file. Instead, we use CRediT to specify the contributions of each author in the journal submission system. Please feel free to use the free text box to provide more detailed descriptions during submission. See also our guide to authors for more information: https://link.springer.com/partners/embo-press/editorial-policies#Authorship.

- Please note that the References format needs to be updated to the style used by The EMBO Journal. In particular, the list of references should be alphabetical (not numerical), and "et al." must be used after the names of the first 10 co-authors of each citation. Please check our author guidelines for more information on our format: https://link.springer.com/journal/44318/submission-guidelines#cms-Reference-guidelines.

- Please upload along with your revised manuscript a complete Author checklist, which you can download from our author guidelines (https://www.embopress.org/page/journal/14602075/authorguide; section "Revised submissions"). Please note that the checklist will also be part of the Review Process File that will be published online in conjunction with the article.

- Please move the funding information in the manuscript to the Acknowledgements section; no separate "Funding" section is

necessary.

- We noticed that there is a "Supplementary Tables" section in the manuscript containing URLs to Google sheets for a Key Resource Table and three more Supplementary Tables. This section should be removed from the manuscript, and each item needs to be uploaded as a separate file in our online system. Please use our template in Word format (which can be downloaded from our Author guidelines: https://link.springer.com/journal/44318/submission-guidelines#cms-Manuscript-organisation-and-formatting) for the "Reagents and Tools Table", containing information on key reagents, oligos, antibodies, experimental models, software, equipment etc., and upload your completed Table as a "Reagents Table" file in our system. The other tables must be uploaded as individual "Dataset EV#" files, each one accompanied by its legend in a separate tab of the same Excel file, and the nomenclature should be updated accordingly throughout the manuscript.

- Please note that EMBO press papers are accompanied online by:
A) a short (2 sentences) summary of the findings and their significance,
B) 2-5 short bullet points highlighting the key results, and
C) a synopsis image in .jpg or .png format that is exactly 550 pixels wide and 300-600 pixels high (the height is variable). Please note that all text needs to be legible at the final size.
Please upload this information along with your revised manuscript (the text for A and B should be provided in a separate Word file).

- At EMBO Press we ask authors to provide source data for the main manuscript Figures. You will receive a separate email with instructions for providing source data with your revised manuscript, including how to upload and organize the files.

- Please remove section "Teaser" from the revised manuscript file.

- The manuscript sections need to be named and ordered as follows: Title page - Abstract - Introduction - Results - Discussion - Methods - Data Availability - Acknowledgements - Disclosure and Competing Interests Statement - References - Figure Legends - main Tables (if there are any) - Expanded View Figure Legends.

- During our standard Figure integrity checks, our team detected possible reuse between Appendix Figure S1A and a Figure of the following published manuscript: Figure 2C Cureus. 2022 Jun 23;14(6):e26247, DOI: 10.7759/cureus.26247. Please check these Figures carefully, replace Appendix Figure S1A if necessary, and clarify and explain any changes in your cover letter or your point-by-point response.

- Our data editors have checked your Figures and their legends, and raised the following queries. Please address all points below completely in your revised manuscript (all changes should be highlighted or "tracked"):
- Please define the annotated p values ****/***/**/* as well as provide the exact p-values for the same in the legends of Figure 4E, G, H, I, J, K; as appropriate.
- Please provide the exact p-values in the legends of Figures 3B, 4C, D; 5B, C, E, F.
- Please note that the box plots need to be defined in terms of minima, maxima, centre, bounds of box and whiskers, and percentile in the legends of Figures 2E, F.
- Please note that information related to "n" is missing in the legends of Figures 2E, F; 3B, 4C-K; 5B, C, E, F.
- Please note that the error bars must be defined in the legends of Figures 4C-K; 5B, C, E, F; 7D, J.

- Please also note that as part of the EMBO Press transparent editorial process, The EMBO Journal publishes online a Peer Review File along with each accepted manuscript. This File will be published in conjunction with your paper and will include the referee reports, your point-by-point responses and all pertinent correspondence relating to the manuscript. Your Author's Checklist will also be published at the end of the Peer Review File. Please let us know in case you want to remove any data or figures from your point-by-point responses before they are published as part of the Peer Review File. Retaining unpublished data in the Peer Review File means that these count as published and that the Peer Review File would need to be referenced in future publications. Please let the editorial office know in case you want to remove any data from this file (contact@embojournal.org).

We look forward to seeing a final version of your manuscript as soon as possible. Please let us know if you have any questions and use this link to submit your revision: https://emboj.msubmit.net/cgi-bin/main.plex.

Best regards,

Ioannis

Ioannis Papaioannou, PhD
Editor, The EMBO Journal

i.papaioannou@embojournal.org

Referee #1:

The revised manuscript is highly improved and the authors have address all important comments in full. This is a very thorough and creative study that brings novel perspective on mammalian brain development and evolution.

Referee #2:

In their revised manuscript, the authors have undertaken substantial additional work to address the concerns raised during peer review by both this reviewer and the others. The feedback from the reviewers where addressing similar points and we find that the authors provide a detailed response and have incorporated new experiments and analyses that directly resolve the technical and conceptual issues previously identified.

A major conceptual advance of the study is the authors' exploration of how different species achieve striking variation in brain size and architecture. Using rigorous lineage-tracing experiments in two species and single-cell RNA sequencing they highlight the duration of the neurogenic period as a critical determinant of neuronal output, given that this window tends to be extended in species with larger brains and encompasses several progenitor subtypes in species-specific proportions. By investigating mechanisms that prolong neurogenesis, the authors propose a rat-specific role for Wnt ligands in extending the neurogenic period. Their identification of a previously uncharacterized radial glial population co-expressing Lmx1a and Lhx2 provides a plausible cellular source for enhanced Wnt ligand secretion in rats.

The revision strengthens the comparative developmental analysis between mice and rats by clarifying the mechanisms underlying species specific differences in neurogenic timing, including a more explicit molecular explanation for the extended early neurogenic period in rats. First, the time lapse imaging experiments and the mathematical modelling, showing that mouse and rat cell cycle duration is similar, strengthen the point that species-specific differences in deep-layer (DL) neuron production are not driven by cell division kinetics. Second, the validation of elevated Wnt signaling levels in rat-specific RG, the correlation of Lmx1a and Wnt ligand genes expression in the rat cortical progenitors and the perturbation experiments of Wnt signalling, by introducing a dominant-negative form of TCF7L2, elegantly and mechanistically show how high level of Wnt are required to have a prolonged neurogenic capacity for DL neurons in rats.

Moreover, the manuscript now integrates multiple complementary datasets, from five different species, resulting in a more comprehensive and coherent account of how developmental speed in neural progenitor cells contributes to divergent brain cell composition across species. This comparison showed that sustained expression of Axin2 and WNT ligands is observed only in the rats, suggesting a unique evolutionary shift for the rat lineage.

This reviewer is satisfied with the revisions and supports the manuscript's publication.

Referee #3:

The authors have been very responsive to my and other reviewers' concerns. They include several additional experiments, clarified text and modified figures. Overall I think this is a nice body of work that addresses an interesting gap in our understanding of evolutionary differences between rodent corticogenesis. I have just a few remaining concerns:

It would be helpful in Figure 4A to put day #s above each arrow to interpret panels C and D.

One suggestion from the prior review was to clarify the impact on radial glia. They now include live imaging of early progenitors (presumed radial glia) to measure cell cycle durations, which they use to model impacts on cell progeny. The authors show there are differences in PH3+ cells in the VZ at early stages (likely measuring radial glia as is the PH3+, though this is not formally shown with any marker). The authors conclude from these and other data assessing Tbr2 cells, that rats have amplifed indirect neurogenesis. However they cannot exclude direct neurogenesis because they don't directly measure radial glia number nor directly quantify cell progeny live from either radial glia or intermediate progenitors. Indeed, their new data and analysis of WNT signaling suggest a clear role of Radial glia and data in Figure 2 imply increased neuron production (which may be direct). I think the authors either should include more direct cell biological analysis of radial glia or simply modify conclusions related to

Figure 4 which suggest that indirect neurogenesis is the main alteration in rats.

***

Rev_Com_number: RC-2025-03240
New_manu_number: EMBOJ-2026-123747-T
Corr_author: Suzuki
Title: Heterochronic scaling of neurogenesis for species-specific dosing of cortical excitatory subtypes

Point-by-point description of the revisions

We kindly request that you address these two points in a final revision, as well as in a point-by-point response to the referees' comments. In particular:
- please include the day numbers in Figure 4A as per the referees' suggestion

-> We added day numbers in Figure 4A as the referees' suggestion. Revised Figure 4 was uploaded in the system.

- please also address the second point of referee #3; no more experimental analyses will be required for the publication of your work in The EMBO Journal, but if you decide not to present more conclusive data, please take the suggestion of the referee into account and tone down the conclusions to more accurately interpret the currently available data in a balanced manner.

-> We appreciate this constructive suggestion. To ensure a more balanced interpretation of the currently available data, we have revised the text and conclusions in the chapter titled "Conserved progenitor kinetics and a modulated contribution of indirect neurogenesis" (P.6 line 40 - P.7 line 14). We revised the chapter title and modified the concluding sentence to read: "Together, these results demonstrate that in addition to the extended duration of the DL production window (Figure 3 and Figure 4), subtle modulations in cell cycle kinetics and the amplification of indirect neurogenesis collaboratively contribute to the species-specific dosage of DL neurons."

1st Revision - Editorial Decision 24th Apr 2026

Dear Dr. Suzuki,

Congratulations on an excellent manuscript! I am very pleased to inform you that it has been accepted for publication in The EMBO Journal. Thank you for comprehensively addressing the initially raised referee concerns and all editorial requests for corrections and changes.

Your manuscript will be processed for publication by EMBO Press. It will be copy edited and you will receive page proofs prior to publication. Please note that you will be contacted by Springer Nature Author Services to complete licensing and payment information.

You may qualify for financial assistance for your publication charges - either via a Springer Nature fully open access agreement or an EMBO initiative. Check your eligibility: https://link.springer.com/journal/44318/how-to-publish-with-us

Should you be planning a Press Release on your article, please get in contact with embo_production@springernature.com as early as possible in order to coordinate publication and release dates.

If you have any questions, please do not hesitate to contact the Editorial Office. Thank you for your contribution to The EMBO Journal. Working with you has been a pleasure.

Best regards,

Ioannis

Ioannis Papaioannou, PhD
Editor, The EMBO Journal
i.papaioannou@embojournal.org

*****
Please note that it is The EMBO Journal policy for the transcript of the editorial process (containing referee reports and your response letters) to be published as an online supplement to each paper. If you should prefer removal of any referee-only figures included in the point-by-point response(s), e.g. because they may still be used for future publication or because they have been reproduced from published work by others, please do let us know immediately via response email.
More information is available here: https://link.springer.com/partners/embo-press/editorial-policies#Peer%20review
*****

***
Rev_Com_number: RC-2025-03240
New_manu_number: EMBOJ-2026-123747R
Corr_author: Suzuki
Title: Heterochronic scaling of neurogenesis for species-specific dosing of cortical excitatory subtypes

EMBO Press Author Checklist

Corresponding Author Name: Ikuo K. Suzuki	
Journal Submitted to: The EMBO Journal	
Manuscript Number: EMBOJ-2026-123747-T	

USEFUL LINKS FOR COMPLETING THIS FORM
The EMBO Journal - Author Guidelines
EMBO Reports - Author Guidelines
Molecular Systems Biology - Author Guidelines
EMBO Molecular Medicine - Author Guidelines

Reporting Checklist for Life Science Articles (updated January
This checklist is adapted from Materials Design Analysis Reporting (MDAR) Checklist for Authors. MDAR establishes a minimum set of requirements in transparent reporting in the life sciences (see Statement of Task: 10.31222/osf.io/9sm4x). Please follow the journal's guidelines in preparing your
Please note that a copy of this checklist will be published alongside your article.

Abridged guidelines for figures
1. Data
The data shown in figures should satisfy the following conditions:
- the data were obtained and processed according to the field's best practice and are presented to reflect the results of the experiments in an accurate and unbiased manner.
- ideally, figure panels should include only measurements that are directly comparable to each other and obtained with the same assay.
- plots include clearly labeled error bars for independent experiments and sample sizes. Unless justified, error bars should not be shown for technical
- if n<5, the individual data points from each experiment should be plotted. Any statistical test employed should be justified.
- Source Data should be included to report the data underlying figures according to the guidelines set out in the authorship guidelines on Data

2. Captions
Each figure caption should contain the following information, for each panel where they are relevant:
- a specification of the experimental system investigated (eg cell line, species name).
- the assay(s) and method(s) used to carry out the reported observations and measurements.
- an explicit mention of the biological and chemical entity(ies) that are being measured.
- an explicit mention of the biological and chemical entity(ies) that are altered/varied/perturbed in a controlled manner.
- the exact sample size (n) for each experimental group/condition, given as a number, not a range;
- a description of the sample collection allowing the reader to understand whether the samples represent technical or biological replicates (including how many animals, litters, cultures, etc.).
- a statement of how many times the experiment shown was independently replicated in the laboratory.
- definitions of statistical methods and measures:
  - common tests, such as t-test (please specify whether paired vs. unpaired), simple χ2 tests, Wilcoxon and Mann-Whitney tests, can be unambiguously identified by name only, but more complex techniques should be described in the methods section;
  - are tests one-sided or two-sided?
  - are there adjustments for multiple comparisons?
  - exact statistical test results, e.g., P values = x but not P values < x;
  - definition of 'center values' as median or average;
  - definition of error bars as s.d. or s.e.m.

Please complete ALL of the questions below.
Select "Not Applicable" only when the requested information is not relevant for your study.

Materials

Newly Created Materials	Information included in the manuscript?	In which section is the information available? (Reagents and Tools Table, Materials and Methods, Figures, Data Availability Section)
New materials and reagents need to be available; do any restrictions apply?	Yes	Plasmids: Methods, "DNA constructs" (P.14 line22) and Structured Methods - Reagents and Tools Table

Antibodies	Information included in the manuscript?	In which section is the information available? (Reagents and Tools Table, Materials and Methods, Figures, Data Availability Section)
For **antibodies** provide the following information:<br>- Commercial antibodies: RRID (if possible) or supplier name, catalogue number and or/clone number<br>- Non-commercial: RRID or citation	Yes	Structured Methods - Reagents and Tools Table

DNA and RNA sequences	Information included in the manuscript?	In which section is the information available? (Reagents and Tools Table, Materials and Methods, Figures, Data Availability Section)
**Short novel DNA or RNA including primers, probes**: provide the sequences.	Not Applicable	

Cell materials	Information included in the manuscript?	In which section is the information available? (Reagents and Tools Table, Materials and Methods, Figures, Data Availability Section)
**Cell lines:** Provide species information, strain. Provide accession number in repository **OR** supplier name, catalog number, clone number, and/**OR** RRID.	Not Applicable	
**Primary cultures:** Provide species, strain, sex of origin, genetic modification status.	Yes	Methods; "Mouse and rat progenitor cell cycle length" (P.15 line18)
Report if the cell lines were recently **authenticated** (e.g., by STR profiling) and tested for mycoplasma contamination.	Not Applicable	

Experimental animals	Information included in the manuscript?	In which section is the information available? (Reagents and Tools Table, Materials and Methods, Figures, Data Availability Section)
**Laboratory animals or Model organisms:** Provide species, strain, sex, age, genetic modification status. Provide accession number in repository **OR** supplier name, catalog number, clone number, **OR** RRID.	Yes	Methods; "Animals" (P.13 line3)
**Animal observed in or captured from the field:** Provide species, sex, and age where possible.	Not Applicable	
Please detail **housing and husbandry conditions**.	Yes	Methods; "Animals" (P.13 line3)

Plants and microbes	Information included in the manuscript?	In which section is the information available? (Reagents and Tools Table, Materials and Methods, Figures, Data Availability Section)
**Plants:** provide species and strain, ecotype and cultivar where relevant, unique accession number if available, and source (including location for collected wild specimens).	Not Applicable	
**Microbes:** provide species and strain, unique accession number if available, and source.	Not Applicable	

Human research participants	Information included in the manuscript?	In which section is the information available? (Reagents and Tools Table, Materials and Methods, Figures, Data Availability Section)
If collected and within the bounds of privacy constraints report on age, sex and gender or ethnicity for all study participants.	Not Applicable	

Core facilities	Information included in the manuscript?	In which section is the information available? (Reagents and Tools Table, Materials and Methods, Figures, Data Availability Section)
If your work benefited from core facilities, was their service mentioned in the acknowledgments section?	Yes	Acknowledgements (P.18 line1)

Design

Study protocol	Information included in the manuscript?	In which section is the information available? (Reagents and Tools Table, Materials and Methods, Figures, Data Availability Section)
If study protocol has been **pre-registered, provide DOI in the manuscript**. For clinical trials, provide the trial registration number **OR** cite DOI.	Not Applicable	
Report the **clinical trial registration number** (at ClinicalTrials.gov or equivalent), where applicable.	Not Applicable	

Laboratory protocol	Information included in the manuscript?	In which section is the information available? (Reagents and Tools Table, Materials and Methods, Figures, Data Availability Section)
Provide DOI OR other citation details if **external detailed step-by-step protocols** are available.	Yes	For single cell transcriptomics, Methods; "Data availability" https://github.com/XD-Sheu/Rodents-temporal-scaling_2026/

Experimental study design and statistics	Information included in the manuscript?	In which section is the information available? (Reagents and Tools Table, Materials and Methods, Figures, Data Availability Section)
Include a statement about **sample size** estimate even if no statistical methods were used.	Yes	All figure legends
Were any steps taken to minimize the effects of subjective bias when allocating animals/samples to treatment (e.g. **randomization procedure**)? If yes, have they been described?	Yes	All figure legends and Methods "Quantification and statistical analysis" (P.17 line27)
Include a statement about **blinding** even if no blinding was done.	Yes	All figure legends and Methods "Quantification and statistical analysis" (P.17 line27)
Describe **inclusion/exclusion criteria** if samples or animals were excluded from the analysis. Were the criteria pre-established? If sample or data points were omitted from analysis, report if this was due to attrition or intentional exclusion and provide justification.	Yes	For clonal analysis, Methods "Clonal analysis" (P.14 line39) For single cell transcriptomics, Methods "Single cell transcriptomics" > "scRNAseq analysis" (P.16 line5)
For every figure, are **statistical tests** justified as appropriate? Do the data meet the assumptions of the tests (e.g., normal distribution)? Describe any methods used to assess it. Is there an estimate of variation within each group of data? Is the variance similar between the groups that are being statistically compared?	Yes	All figure legends and Methods "Quantification and statistical analysis" (P.17 line27)

Sample definition and in-laboratory replication	Information included in the manuscript?	In which section is the information available? (Reagents and Tools Table, Materials and Methods, Figures, Data Availability Section)
In the figure legends: state number of times the experiment was **replicated** in laboratory.	Yes	All figure legends
In the figure legends: define whether data describe **technical or biological replicates**.	Yes	All figure legends

Ethics

Ethics	Information included in the manuscript?	In which section is the information available? (Reagents and Tools Table, Materials and Methods, Figures, Data Availability Section)
Studies involving **human participants**: State details of **authority granting ethics approval** (IRB or equivalent committee(s), provide reference number for approval.	Not Applicable	
Studies involving **human participants**: Include a statement confirming that **informed consent** was obtained from all subjects and that the experiments conformed to the principles set out in the WMA Declaration of Helsinki and the Department of Health and Human Services Belmont Report.	Not Applicable	
Studies involving **human participants:** For publication of **patient photos**, include a statement confirming that consent to publish was obtained.	Not Applicable	
Studies involving experimental **animals**: State details of **authority granting ethics approval** (IRB or equivalent committee(s), provide reference number for approval. Include a statement of compliance with ethical regulations.	Yes	Methods; "Animals" (P.13 line3)
Studies involving **specimen and field samples:** State if relevant **permits** obtained, provide details of authority approving study; if none were required, explain why.	Not Applicable	

Dual Use Research of Concern (DURC)	Information included in the manuscript?	In which section is the information available? (Reagents and Tools Table, Materials and Methods, Figures, Data Availability Section)
Could your study fall under dual use research restrictions? Please check biosecurity documents and list of **select agents and toxins** (CDC): https://www.selectagents.gov/sat/list.htm	Not Applicable	
If you used a select agent, is the security level of the lab appropriate and reported in the manuscript?	Not Applicable	
If a study is subject to dual use research of concern regulations, is the name of the **authority granting approval and reference number** for the regulatory approval provided in the manuscript?	Not Applicable	

Reporting

The MDAR framework recommends adoption of discipline-specific guidelines, established and endorsed through community initiatives. Journals have their own policy about requiring specific guidelines and recommendations to complement MDAR.

Adherence to community standards	Information included in the manuscript?	In which section is the information available? (Reagents and Tools Table, Materials and Methods, Figures, Data Availability Section)
State if relevant guidelines or checklists (e.g., **ICMJE, MIBBI, ARRIVE, PRISMA**) have been followed or provided.	Not Applicable	
For **tumor marker prognostic studies,** we recommend that you follow the **REMARK** reporting guidelines (see link list at top right). See author guidelines, under 'Reporting Guidelines'. Please confirm you have followed these guidelines.	Not Applicable	
For **phase II and III randomized controlled trials**, please refer to the **CONSORT** flow diagram (see link list at top right) and submit the CONSORT checklist (see link list at top right) with your submission. See author guidelines, under 'Reporting Guidelines'. Please confirm you have submitted this list.	Not Applicable	

Data Availability

Data availability	Information included in the manuscript?	In which section is the information available? (Reagents and Tools Table, Materials and Methods, Figures, Data Availability Section)
Have **primary datasets** been deposited according to the journal's guidelines (see 'Data Deposition' section) and the respective accession numbers provided in the Data Availability Section?	Yes	**Data Availability**
Were **human clinical and genomic datasets** deposited in a public access-controlled repository in accordance to ethical obligations to the patients and to the applicable consent agreement?	Not Applicable	
Are **computational models** that are central and integral to a study available without restrictions in a machine-readable form? Were the relevant accession numbers or links provided?	Yes	Data Availability (P.17 line38)
If publicly available data were reused, provide the respective **data citations in the reference list**.	Yes	Methods "Single cell transcriptomics" > "Gene Expression Dynamics Across Five Mammalian Species" (P.17 line5)
