# Supplementary material for: Interspecific diversity in the neuronal composition of the mammalian cortex arises from heterochrony in neurogenesis
Source: EMBO J. 2026 May 22;45(15):5237–66. doi: 10.1038/s44318-026-00806-z (PMC13434014; doi:10.1038/s44318-026-00806-z)
Supplement: Supplementary file 3 [file 44318_2026_806_MOESM5_ESM.pdf]

Expanded View Figures

Figure EV1. Clonal analysis of cortical RG progenitors in mice and rats from the onset to the end of excitatory neurogenesis, related to Fig. 2.

(**A**) Schematic representation of hemi-lineage labeling via retroviral gene transfer, illustrating three scenarios of progenitor labeling. Cortical progenitors initially divide symmetrically to expand their population before transitioning to neurogenesis. Radial glial (RG) progenitors begin producing neurons by switching from symmetric proliferative divisions to asymmetric neurogenic divisions. Eventually, RG progenitors terminate producing neurons to begin gliogenesis. A clonal "unit" of neurons is defined as sister excitatory neurons that share a common progenitor origin and are produced during the first and subsequent neurogenic divisions of the mother progenitor (rounded boxes). Case 1: The retroviral vector infects a progenitor undergoing symmetric proliferative divisions. This results in multiple clonal units being labeled within the same cell cluster. Case 2: The retroviral vector infects an RG progenitor at its last proliferative division before initiating neurogenesis. In this ideal case, the entire clonal unit of sister neurons is labeled. Case 3: The retroviral vector infects an RG progenitor that has already started neurogenic divisions. In this scenario, some members of the clonal unit are missing from the labeled cohort, resulting in an incomplete clone. Alternatively, only one neuron (direct neurogenesis) or two neurons (indirect neurogenesis via intermediate progenitors) may be labeled. Given the heterogeneity in cortical progenitor division statuses, actual experimental data represent a mixture of these three cases. In Case 3, where neurogenic progenitors dominate, half of the clones are expected to consist of one or two neurons. Therefore, twice the proportion of one- or two-neuron clones serves as an estimate of the ratio of neurogenic progenitors at the time of retroviral injection. (**B**) Representative coronal section showing three labeled clones (RFP in magenta, GFP in green) with spatial separation and no overlap. (**C**) Representative images of neurons and glial cells. NeuN was used as a neuronal marker, whereas Olig2 and Sox9 were used to identify glial cells. Arrowheads denote GFP-positive cells co-expressing the indicated markers. (**D**) Representative clonal analysis following retroviral injection at E12.5 and tissue collection at P7. Cortical sections were immunostained for GFP together with the projection neuron markers Satb2, Ctip2, and Tbr1. Panels show the cortical location of GFP-labeled cells and their corresponding marker expression. (**E**) EdU labeling of postnatal (P1, P3, P6) mice and rats reveals co-labeling with Olig2 (glial progenitors) but not NeuN (neurons), suggesting no neurogenesis in the postnatal period in both species. (**F**) Neuron-progenitor ratio defined by Pax6 (green) and beta III tubulin (magenta) expression demonstrates developmental stage correspondence between species with a 2-day difference. E12.5 in mice and E14.5 in rats are the corresponding timing of onset of neurogenesis for the majority of cortical progenitors (yellow background). Scale bars: 1 mm (low magnification, (**B**), 100 μm (magnified images, (**B**), 50 μm (**C**), 200 μm (low magnification, (**D**), 20 μm (magnified images, (**D**), 50 μm (**E**), 200 μm (**F**).

 © The Author(s)

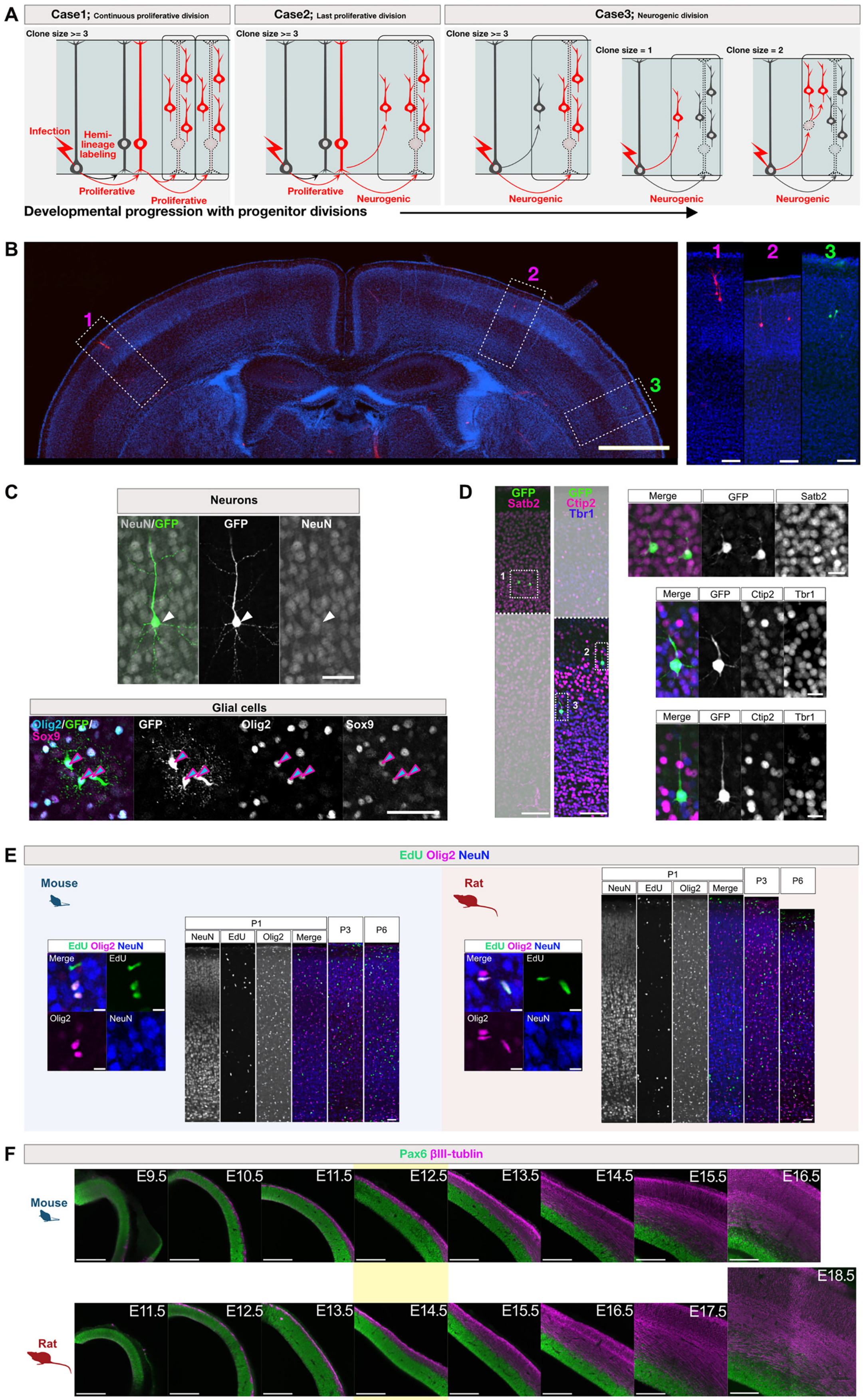
A
Case1; Continuous proliferative division
Case2; Last proliferative division
Case3; Neurogenic division
Clone size >= 3
Clone size = 1
Clone size = 2
Infection
Hemi-lineage labeling
Proliferative
Neurogenic
Developmental progression with progenitor divisions
B
1
2
3
C
Neurons
NeuN/GFP
GFP
NeuN
Glial cells
Olig2/GFP/Sox9
Olig2
Sox9
D
GFP
Satb2
Ctip2
Tbr1
Merge
E
EdU Olig2 NeuN
Mouse
Rat
Merge
EdU
Olig2
NeuN
P1
P3
P6
F
Pax6 βIII-tublin
E9.5
E10.5
E11.5
E12.5
E13.5
E14.5
E15.5
E16.5
E17.5
E18.5

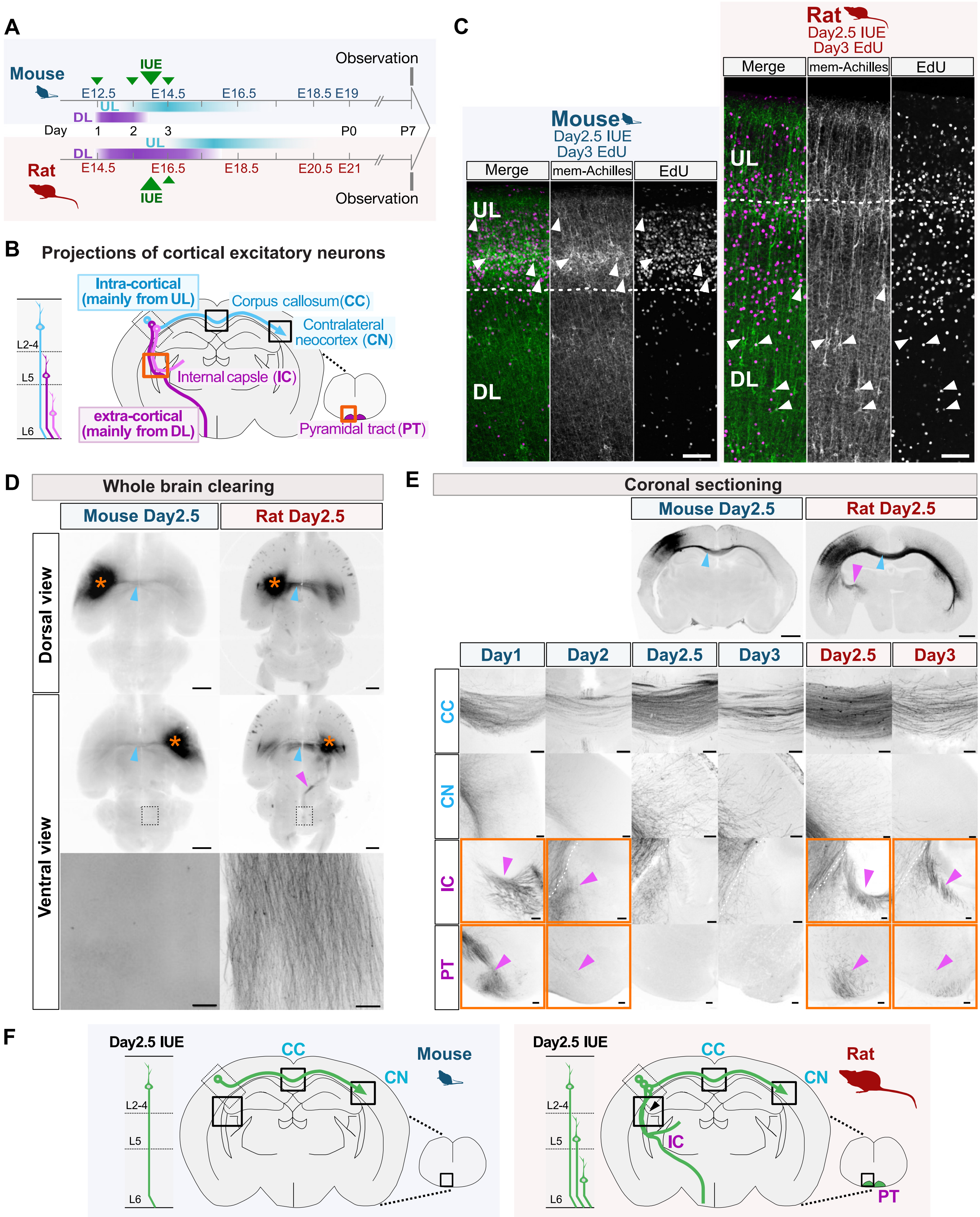
A
Mouse
IUE
Observation
E12.5
E14.5
E16.5
E18.5 E19
UL
DL
Day
1
2
3
P0
P7
UL
DL
Rat
E14.5
E16.5
E18.5
E20.5 E21
IUE
Observation
B
Projections of cortical excitatory neurons
Intra-cortical (mainly from UL)
Corpus callosum (CC)
Contralateral neocortex (CN)
L2-4
L5
L6
Internal capsle (IC)
extra-cortical (mainly from DL)
Pyramidal tract (PT)
C
Rat
Day2.5 IUE
Day3 EdU
Merge
mem-Achilles
EdU
Mouse
Day2.5 IUE
Day3 EdU
Merge
mem-Achilles
EdU
UL
DL
UL
DL
D
Whole brain clearing
Mouse Day2.5
Rat Day2.5
Dorsal view
Ventral view
E
Coronal sectioning
Mouse Day2.5
Rat Day2.5
Day1
Day2
Day2.5
Day3
Day2.5
Day3
CC
CN
IC
PT
F
Day2.5 IUE
CC
Mouse
CN
L2-4
L5
L6
Day2.5 IUE
CC
Rat
CN
L2-4
L5
L6
IC
PT

 © The Author(s)

Figure EV2. Projection identity of neurons generated at Day 2.5–3 of neurogenesis in mice and rats, related to Fig. 4.

(**A**) Schematic of IUE timing and sacrifice for axon projection analysis. (**B**) Major projection targets of cortical excitatory neurons: corpus callosum (CC), contralateral neocortex (CN), internal capsule (IC), and pyramidal tract (PT). (**C**) The neurons electroporated at Day 2.5 are co-labeled with EdU injected at Day 3 in both species. (**D**) Tissue-cleared whole-brain images (dorsal and ventral views) show distinct projection patterns. In rats, prominent ipsilateral projections to the brainstem (magenta arrowheads) are unique. (**E**) Coronal sections confirm unique descending projections in rats and their absence in mice after Day 3 IUE. (**F**) Summary illustration of projection differences between mouse and rat neurons generated at Days 2.5–3. Scale bars: 1 mm (low magnification, (**C**, **D**), 100 µm (magnified images, (**D**, **E**).

© The Author(s)

Figure EV3. Gene co-expression patterns in cortical progenitors across five mammalian species, related to Fig. 7.

(**A**) Co-expression patterns of *Emx2* and *Lhx2* in cortical progenitors across five mammalian species (rat, mouse, ferret, macaque, and human). Expression values were retrieved from the SCT assay in each regressed object. (**B**) Co-expression patterns of *Lmx1a* and *Lhx2* in cortical progenitors across five mammalian species (rat, mouse, ferret, macaque, and human). Expression values were retrieved from the SCT assay in each regressed object. The right bottom panel is the double-positive ratio of *Lhx2* and *Lmx1a* in cortical progenitors across five mammalian species. Cells with gene expression values above 0.1 for a given gene were defined as positive for that gene. (**C**) Co-expression of Wnt ligand genes ($n = 11$) with Lmx1a in rat cortical progenitors. Gene expression data retrieved from SCT assay.

© The Author(s)

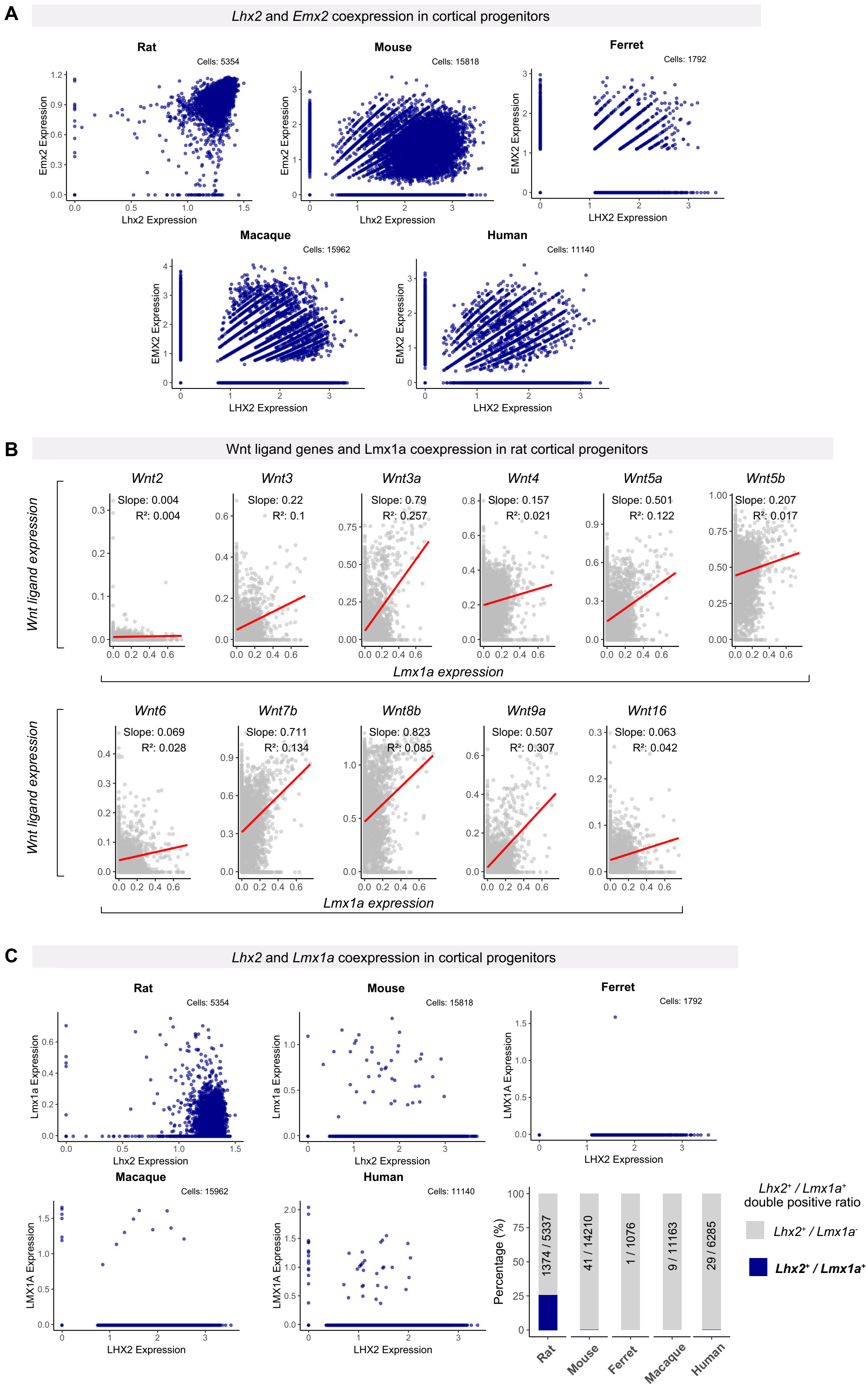
A
Lhx2 and Emx2 coexpression in cortical progenitors
Rat
Cells: 5354
Emx2 Expression
Lhx2 Expression
Mouse
Cells: 15818
Emx2 Expression
Lhx2 Expression
Ferret
Cells: 1792
EMX2 Expression
LHX2 Expression
Macaque
Cells: 15962
EMX2 Expression
LHX2 Expression
Human
Cells: 11140
EMX2 Expression
LHX2 Expression
B
Wnt ligand genes and Lmx1a coexpression in rat cortical progenitors
Wnt ligand expression
Wnt2
Slope: 0.004
R²: 0.004
Wnt3
Slope: 0.22
R²: 0.1
Wnt3a
Slope: 0.79
R²: 0.257
Wnt4
Slope: 0.157
R²: 0.021
Wnt5a
Slope: 0.501
R²: 0.122
Wnt5b
Slope: 0.207
R²: 0.017
Lmx1a expression
Wnt ligand expression
Wnt6
Slope: 0.069
R²: 0.028
Wnt7b
Slope: 0.711
R²: 0.134
Wnt8b
Slope: 0.823
R²: 0.085
Wnt9a
Slope: 0.507
R²: 0.307
Wnt16
Slope: 0.063
R²: 0.042
Lmx1a expression
C
Lhx2 and Lmx1a coexpression in cortical progenitors
Rat
Cells: 5354
Lmx1a Expression
Lhx2 Expression
Mouse
Cells: 15818
Lmx1a Expression
Lhx2 Expression
Ferret
Cells: 1792
LMX1A Expression
LHX2 Expression
Macaque
Cells: 15962
LMX1A Expression
LHX2 Expression
Human
Cells: 11140
LMX1A Expression
LHX2 Expression
Percentage (%)
1374 / 5337
41 / 14210
1 / 1076
9 / 11163
29 / 6285
Rat
Mouse
Ferret
Macaque
Human
Lhx2⁺ / Lmx1a⁺ double positive ratio
Lhx2⁺ / Lmx1a⁻
Lhx2⁺ / Lmx1a⁺

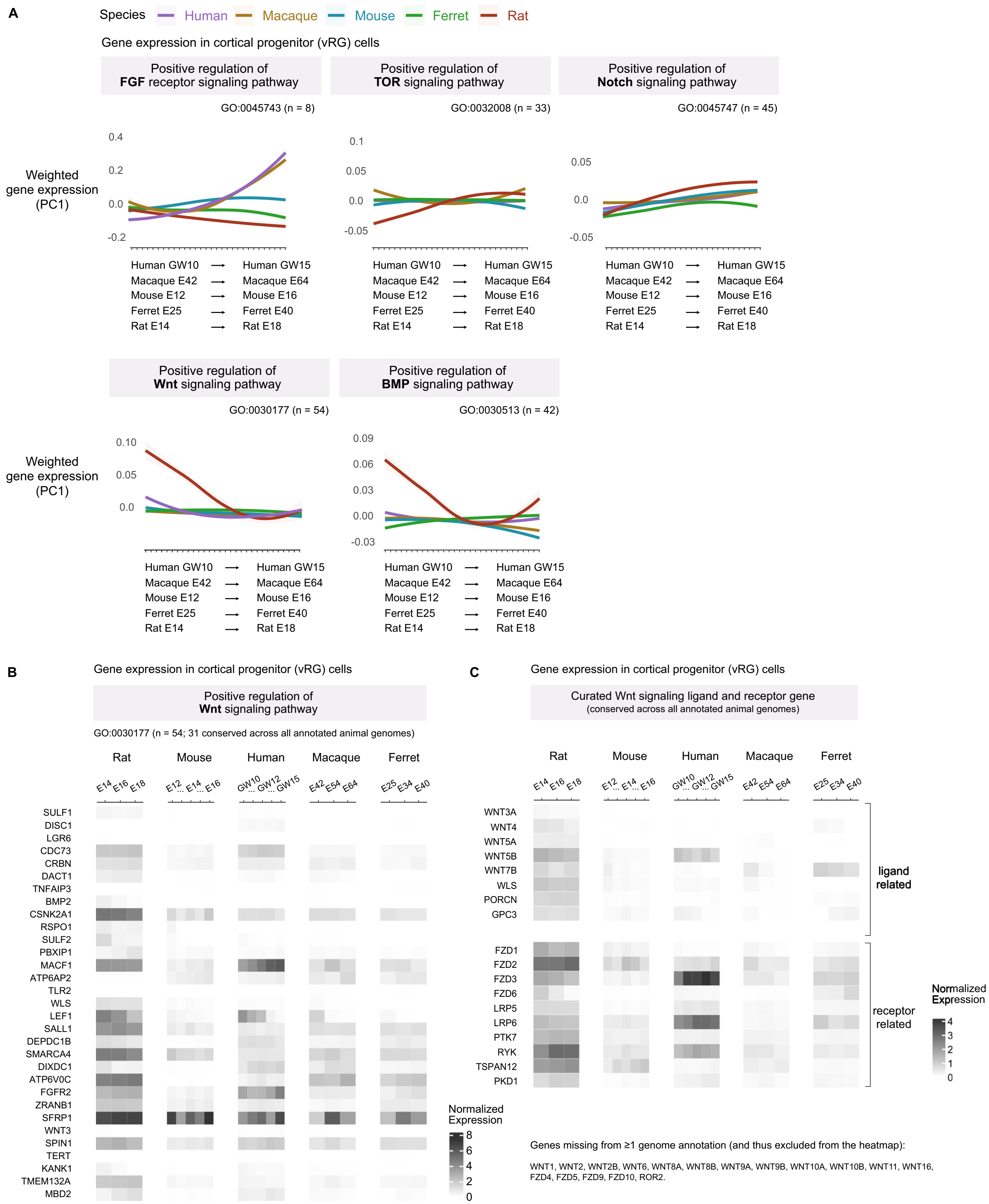
A
Species Human Macaque Mouse Ferret Rat
Gene expression in cortical progenitor (vRG) cells
Positive regulation of FGF receptor signaling pathway
GO:0045743 (n = 8)
Positive regulation of TOR signaling pathway
GO:0032008 (n = 33)
Positive regulation of Notch signaling pathway
GO:0045747 (n = 45)
Positive regulation of Wnt signaling pathway
GO:0030177 (n = 54)
Positive regulation of BMP signaling pathway
GO:0030513 (n = 42)
Weighted gene expression (PC1)
Human GW10 → Human GW15
Macaque E42 → Macaque E64
Mouse E12 → Mouse E16
Ferret E25 → Ferret E40
Rat E14 → Rat E18
B
Gene expression in cortical progenitor (vRG) cells
Positive regulation of Wnt signaling pathway
GO:0030177 (n = 54; 31 conserved across all annotated animal genomes)
Rat Mouse Human Macaque Ferret
SULF1
DISC1
LGR6
CDC73
CRBN
DACT1
TNFAIP3
BMP2
CSNK2A1
RSPO1
SULF2
PBXIP1
MACF1
ATP6AP2
TLR2
WLS
LEF1
SALL1
DEPDC1B
SMARCA4
DIXDC1
ATP6V0C
FGFR2
ZRANB1
SFRP1
WNT3
SPIN1
TERT
KANK1
TMEM132A
MBD2
Normalized Expression
C
Gene expression in cortical progenitor (vRG) cells
Curated Wnt signaling ligand and receptor gene
(conserved across all annotated animal genomes)
WNT3A
WNT4
WNT5A
WNT5B
WNT7B
WLS
PORCN
GPC3
ligand related
FZD1
FZD2
FZD3
FZD6
LRP5
LRP6
PTK7
RYK
TSPAN12
PKD1
receptor related
Normalized Expression
Genes missing from ≥1 genome annotation (and thus excluded from the heatmap):
WNT1, WNT2, WNT2B, WNT6, WNT8A, WNT8B, WNT9A, WNT9B, WNT10A, WNT10B, WNT11, WNT16, FZD4, FZD5, FZD9, FZD10, ROR2.

 © The Author(s)

Figure EV4. Major signaling pathways regulating brain development in cortical progenitors of five mammalian species, related to Fig. 7.

(**A**) Weighted summary (PC1) of gene expression for involved genes, retrieved from the corresponding Gene Ontology term (positive regulation only). Ventricular radial glia (vRG) from equivalent developmental stages in human (GW10–GW15), macaque (E42–E64), mouse (E12–E16), ferret (E25–E40), and rat (E14–E18) were analyzed. (**B**) Heatmap of gene expression in cortical progenitors, highlighting genes from "Positive regulation of Wnt signaling pathway" (GO:0030177). (**C**) Heatmap of gene expression in cortical progenitors of curated Wnt signaling-related ligand and receptor genes. Genes not conserved across all annotated animal genomes were excluded from the heatmap and are listed below it.

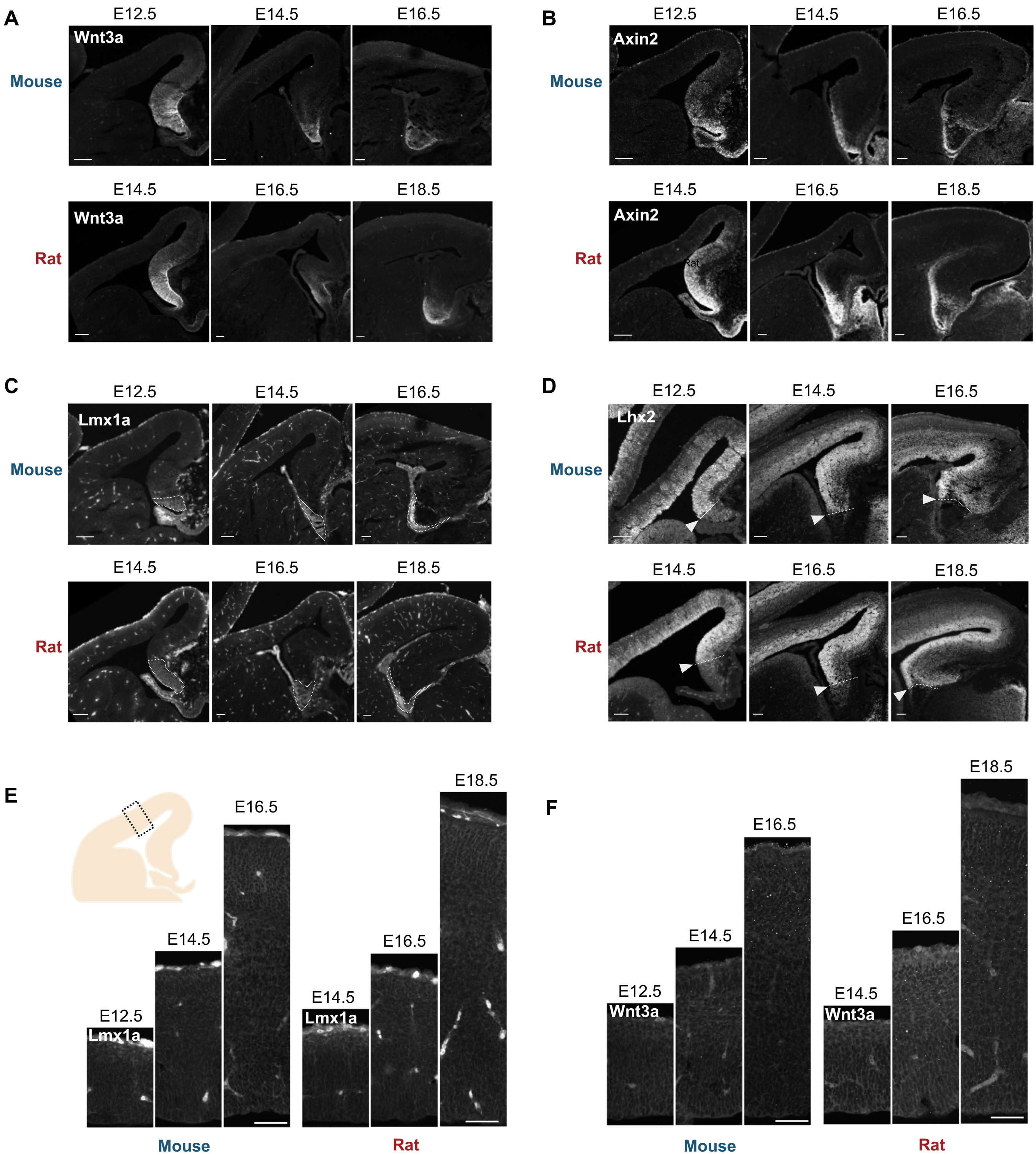

Figure EV5. Species-specific temporal dynamics of cortical hem-associated Wnt signaling during corticogenesis, related to Fig. 7.

(**A**) Immunostaining for Wnt3a in coronal sections of developing mouse and rat cortices at the indicated embryonic stages. (**B**) In situ hybridization of Axin2, a canonical Wnt signaling target gene, in mouse and rat cortices across developmental stages. (**C**) In situ hybridization of Lmx1a, a cortical hem marker, in mouse and rat embryos at corresponding stages. Outlined regions indicate the cortical hem. (**D**) Immunostaining for Lhx2, a transcription factor expressed in cortical neuroepithelium and excluded from the cortical hem. Arrowheads indicate the hem–cortex boundary. (**E**, **F**) Representative images of Lmx1a (**E**) and Wnt3a (**F**) expression in the dorsomedial cortex of mouse and rat embryos at the indicated developmental stages. Comparable cortical regions were analyzed. Scale bars: 100 µm (**A–D**), 50 µm (**E**, **F**).

© The Author(s)
